# Quantitative Fitness Analysis Shows That NMD Proteins and Many Other Protein Complexes Suppress or Enhance Distinct Telomere Cap Defects

**DOI:** 10.1371/journal.pgen.1001362

**Published:** 2011-04-07

**Authors:** Stephen Gregory Addinall, Eva-Maria Holstein, Conor Lawless, Min Yu, Kaye Chapman, A. Peter Banks, Hien-Ping Ngo, Laura Maringele, Morgan Taschuk, Alexander Young, Adam Ciesiolka, Allyson Lurena Lister, Anil Wipat, Darren James Wilkinson, David Lydall

**Affiliations:** 1 Institute for Cell and Molecular Biosciences, Newcastle University Medical School, Newcastle upon Tyne, United Kingdom; 2 Centre for Integrated Systems Biology of Ageing and Nutrition, Institute for Ageing and Health, Newcastle University Campus for Ageing and Vitality, Newcastle upon Tyne, United Kingdom; 3 Crucible Laboratory, Institute for Ageing and Health, Newcastle University Centre for Life, Newcastle upon Tyne, United Kingdom; 4 School of Computing Science, Newcastle University, Newcastle Upon Tyne, United Kingdom; 5 School of Mathematics and Statistics, Newcastle University, Newcastle upon Tyne, United Kingdom; Fred Hutchinson Cancer Research Center, United States of America

## Abstract

To better understand telomere biology in budding yeast, we have performed systematic suppressor/enhancer analyses on yeast strains containing a point mutation in the essential telomere capping gene *CDC13* (*cdc13-1*) or containing a null mutation in the DNA damage response and telomere capping gene *YKU70* (*yku70*Δ). We performed Quantitative Fitness Analysis (QFA) on thousands of yeast strains containing mutations affecting telomere-capping proteins in combination with a library of systematic gene deletion mutations. To perform QFA, we typically inoculate 384 separate cultures onto solid agar plates and monitor growth of each culture by photography over time. The data are fitted to a logistic population growth model; and growth parameters, such as maximum growth rate and maximum doubling potential, are deduced. QFA reveals that as many as 5% of systematic gene deletions, affecting numerous functional classes, strongly interact with telomere capping defects. We show that, while Cdc13 and Yku70 perform complementary roles in telomere capping, their genetic interaction profiles differ significantly. At least 19 different classes of functionally or physically related proteins can be identified as interacting with *cdc13-1*, *yku70*Δ, or both. Each specific genetic interaction informs the roles of individual gene products in telomere biology. One striking example is with genes of the nonsense-mediated RNA decay (NMD) pathway which, when disabled, suppress the conditional *cdc13-1* mutation but enhance the null *yku70*Δ mutation. We show that the suppressing/enhancing role of the NMD pathway at uncapped telomeres is mediated through the levels of Stn1, an essential telomere capping protein, which interacts with Cdc13 and recruitment of telomerase to telomeres. We show that increased Stn1 levels affect growth of cells with telomere capping defects due to *cdc13-1* and *yku70*Δ. QFA is a sensitive, high-throughput method that will also be useful to understand other aspects of microbial cell biology.

## Author Summary

Telomeres, specialized structures at the end of linear chromosomes, ensure that chromosome ends are not mistakenly treated as DNA double-strand breaks. Defects in the telomere cap contribute to ageing and cancer. In yeast, defects in telomere capping proteins can cause telomeres to behave like double-strand breaks. To better understand the telomere and responses to capping failure, we have combined a systematic yeast gene deletion library with mutations affecting important yeast telomere capping proteins, Cdc13 or Yku70. Quantitative Fitness Analysis (QFA) was used to accurately measure the fitness of thousands of different yeast strains containing telomere capping defects and additional deletion mutations. Interestingly, we find that many gene deletions suppress one type of telomere capping defect while enhancing another. Through QFA, we can begin to define the roles of different gene products in contributing to different aspects of the telomere cap. Strikingly, mutations in nonsense-mediated mRNA decay pathways, which degrade many RNA molecules, suppress the *cdc13-1* defect while enhancing the *yku70*Δ defect. QFA is widely applicable and will be useful for understanding other aspects of yeast cell biology.

## Introduction

Linear chromosome ends must be protected from the DNA damage response machinery and from shortening of chromosome ends during DNA replication [1], [2]. Chromosome ends therefore adopt specialized structures called telomeres, distinct from double-stranded DNA breaks elsewhere in the genome. Telomeric DNA is protected, or capped and replicated by a large number of different DNA-binding proteins in all eukaryotic cell types [2], [3].

In budding yeast, numerous proteins contribute to telomere capping and amongst these are two critical protein complexes, the Yku70/Yku80 (Ku) heterodimer and the Cdc13/Stn1/Ten1 (CST) heterotrimeric complex [4]. Orthologous protein complexes play roles at telomeres in other eukaryotic cell types suggesting that understanding the function of the Ku and CST protein complexes in budding yeast will be generally informative about key aspects of eukaryotic telomere structure and function.

In budding yeast Yku70 is a non-essential protein that has multiple roles in DNA repair and at telomeres, being involved in the non-homologous end-joining (NHEJ) DNA repair pathway, in the protection of telomeres and the recruitment of telomerase. The mammalian orthologue, Ku70, has similar properties [5]. In budding yeast, deletion of the *YKU70* gene (*yku70*Δ) results in short telomeres and temperature sensitivity [6]. At high temperatures, cells lacking Yku70 accumulate ssDNA at telomeres, which activates the DNA damage response and leads to cell-cycle arrest [7], [8], [9].

Cdc13 is a constituent of the essential budding yeast Cdc13-Stn1-Ten1 (CST) protein complex which is analogous to the CST complex found recently in mammalian, plant and fission yeast cells [10], [11]. Cdc13 binds to ssDNA overhangs at telomeres and functions in telomerase recruitment and telomere capping [12], [13], [14]. Acute inactivation of Cdc13 by the temperature sensitive *cdc13-1* allele induces ssDNA generation at telomeres and rapid, potent checkpoint-dependent cell cycle arrest [14].

*cdc13-1* or *yku70*Δ mutations each cause temperature dependent disruption of telomere capping that is accompanied by ssDNA production, cell-cycle arrest and cell death [7], [15]. Interestingly, the poor growth imparted by each mutation can be suppressed by deletion of *EXO1*, removing the Exo1 nuclease that contributes to ssDNA production when either Cdc13 or Yku70 is defective [7]. However, *cdc13-1* and *yku70*Δ mutations show a synthetic poor growth interaction [8] and different checkpoint pathways are activated by each mutation [7]. These latter observations, along with numerous others, show that CST and Ku complexes perform distinct roles capping budding yeast telomeres and that further clarification of their functions at the telomere is important to help understand how eukaryotic telomeres function.

Many insights into the telomere cap and the DNA damage responses induced when capping is defective were first identified as genetic interactions. For example all DNA damage checkpoint mutations suppress the temperature sensitive growth of *cdc13-1* mutants [16], but only a subset of these suppress the temperature sensitive growth of *yku70*Δ mutants [7]. We reasoned that the roles of Cdc13 and Yku70 at telomeres could be further understood by quantitative, systematic analysis of genetic interactions between telomere capping mutations and a genome-wide collection of gene deletions.

We used standard synthetic genetic array (SGA) approaches to combine the systematic gene deletion collection with *cdc13-1* and *yku70*Δ mutations [17], [18]. After this, strain fitnesses were measured at a number of temperatures by quantitative fitness analysis (QFA). For QFA, liquid cultures were spotted onto solid agar plates and culture growth was followed by time course photography. Images were processed and fitted to a logistic growth model to allow an accurate estimation of growth parameters, such as doubling time. In other high-throughput experiments such as SGA or EMAP approaches, culture fitness is determined from colony size [17], [18], [19]. In QFA, analysis of growth curves of cultures grown on solid agar plates allows us to measure fitness more precisely.

Through QFA we identify hundreds of gene deletions, in numerous different classes, showing genetic interactions with *cdc13-1*, *yku70*Δ or both. One particularly striking example of the type of genetic interactions we measured by QFA is between deletions affecting nonsense mediated RNA decay pathways (*upf1*Δ, *upf2*Δ, *upf3*Δ), *cdc13-1* and *yku70*Δ. Additional experiments show that disabling nonsense mediated mRNA decay pathways, using *upf2*Δ as an example, suppresses the *cdc13-1* defect but enhances the *yku70*Δ defect by increasing the levels of the telomere capping protein Stn1. QFA is generally applicable and will be useful for understanding other aspects of yeast cell biology or studying other microorganisms.

## Results

### QFA identifies gene deletions that interact with *cdc13-1* and *yku70*Δ

To systematically examine genetic interactions between a genome-wide collection of gene deletion strains (*yfg*Δ, **y**our **f**avorite **g**ene **d**eletion, to indicate any of ∼4200 viable systematic gene deletions) and mutations causing telomere capping defects we crossed the knockout library to *cdc13-1* or *yku70*Δ mutations, each affecting the telomere, or to a neutral control query mutation (*ura3*Δ) using SGA methodology [17], [18]. Since both *cdc13-1* and *yku70*Δ mutations cause temperature sensitive defects, we generated all double mutants at low, permissive temperatures before measuring the growth of double mutants at a number of semi-permissive or non-permissive temperatures. We cultured *yku70*Δ *yfg*Δ strains at 23°C, 30°C, 37°C and 37.5°C, *cdc13-1 yfg*Δ strains at 20°C, 27°C and 36°C and *ura3*Δ *yfg*Δ strains at 20°C, 27°C and 37°C and measured fitness.

**Figure 1:**
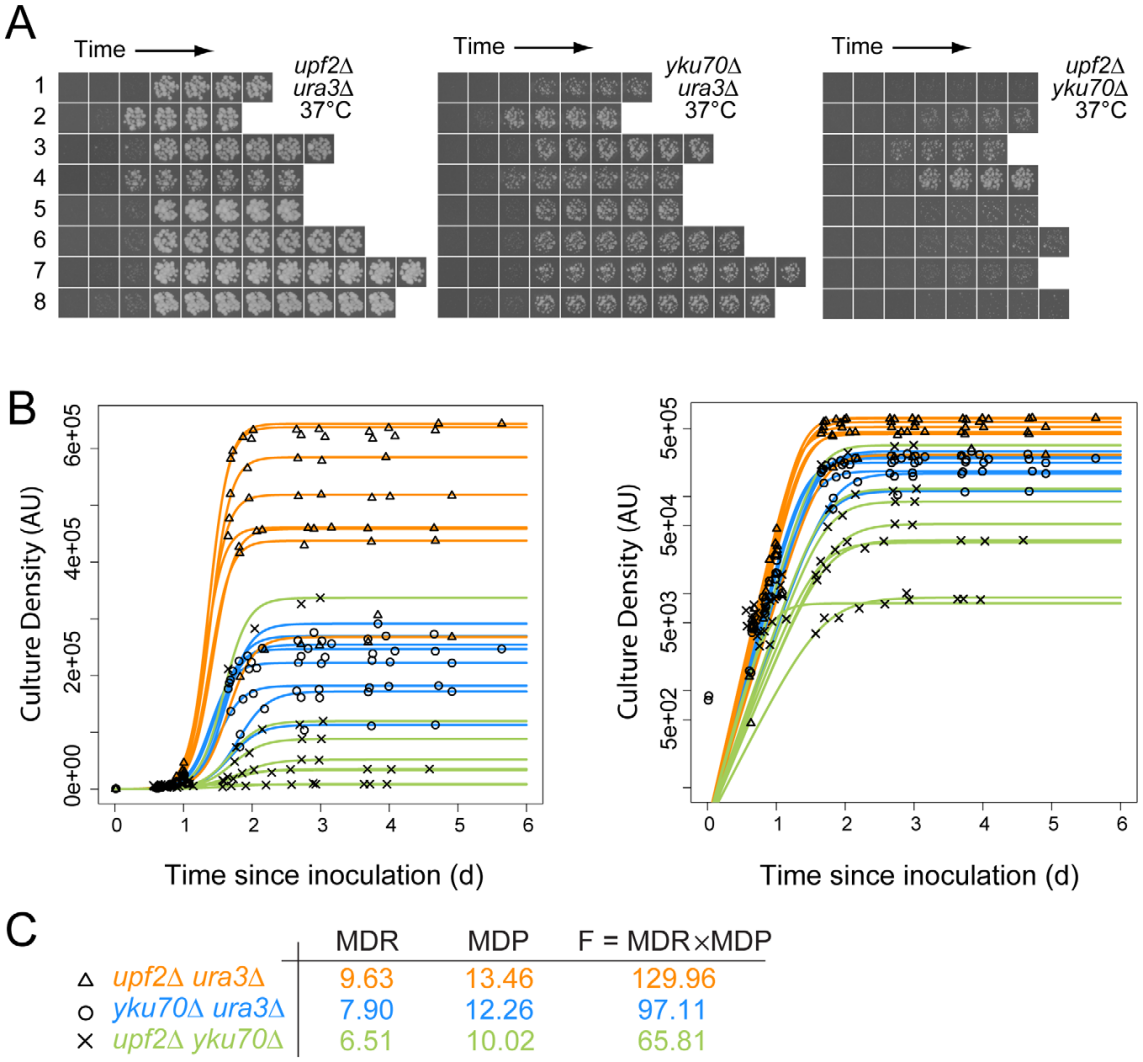
Cell fitness determination from growth on agar plates for quantitative fitness analysis (QFA). A) Time course images of eight independent *upf2*Δ *ura3*Δ, *yku70*Δ *ura3*Δ and *upf2*Δ *yku70*Δ strains at the indicated temperatures; B) Cell density of individual replicate cultures was determined after image-analysis. The logistic growth model is fitted to each culture density time-series. The same data are plotted on linear or logarithmic scales on left and right respectively. C) Average values for Maximum Doubling Rate, Maximum Doubling Potential and Fitness (MDR, MDP and F respectively; see Text S1, experimental procedures), determined from the fitted curves. Data for *yku70*Δ *ura3*Δ is presented here to illustrate epistasis between *yku70*Δ and *upf3*Δ, however this is not how epistasis was calculated (see Figure 2 and Text S1, experimental procedures).

Double mutant fitness was measured after spotting of dilute liquid cultures onto solid agar. We estimate approximately 100 separate cells were placed in each of 384 spots on each agar plate. Fitness of thousands of individual cultures, each derived from spotted cells, was deduced by time course photography of agar plates followed by image processing, data analysis, fitting of growth measurements to a logistic model and determination of quantitative growth parameters (Figure 1) [20], [21], [22]. We fitted logistic growth model parameters to growth curves allowing us to estimate maximum doubling rate (MDR, population doublings/day) and maximum doubling potential (MDP, population doublings) of approximately 12,000 different yeast genotypes (e.g. *cdc13-1 yfg1*Δ, *yku70*Δ *yfg1*Δ, etc.) at several temperatures. At least eight independent biological replicates for each strain at each temperature were cultured and repeatedly photographed, capturing more than 4 million images in total. To rank fitness we assigned equal importance to maximum doubling rate and maximum doubling potential and defined strain fitness as the product of the MDR and MDP values (Fitness, F, population doublings^2^/day). Other measures of fitness can be derived from the sets of logistic parameters available from Text S1.

Figure 1A shows approximately 170 example images, corresponding to eight independent time courses for each of three pair-wise combinations of *yku70*Δ, *ura3*Δ and *upf2*Δ mutations. These example images clearly show, qualitatively, that *upf2*Δ *yku70*Δ strains grow less well than *yku70*Δ *ura3*Δ strains, which in turn grow less well than *upf2*Δ *ura3*Δ strains at 37°C. These fitness measures are consistent with numerous earlier studies, showing that *yku70*Δ mutants do not grow well at high temperatures, but also demonstrate a novel observation, that the *upf2*Δ mutation enhances the *yku70*Δ defect and this is further investigated below. Images like those in Figure 1A were processed, quantified, plotted and logistic growth curves fitted to the data (Figure 1B). We applied QFA to all genotypes at each temperature, as the three examples in Figure 1C illustrate.

### QFA of telomere capping mutants

**Figure 2:**
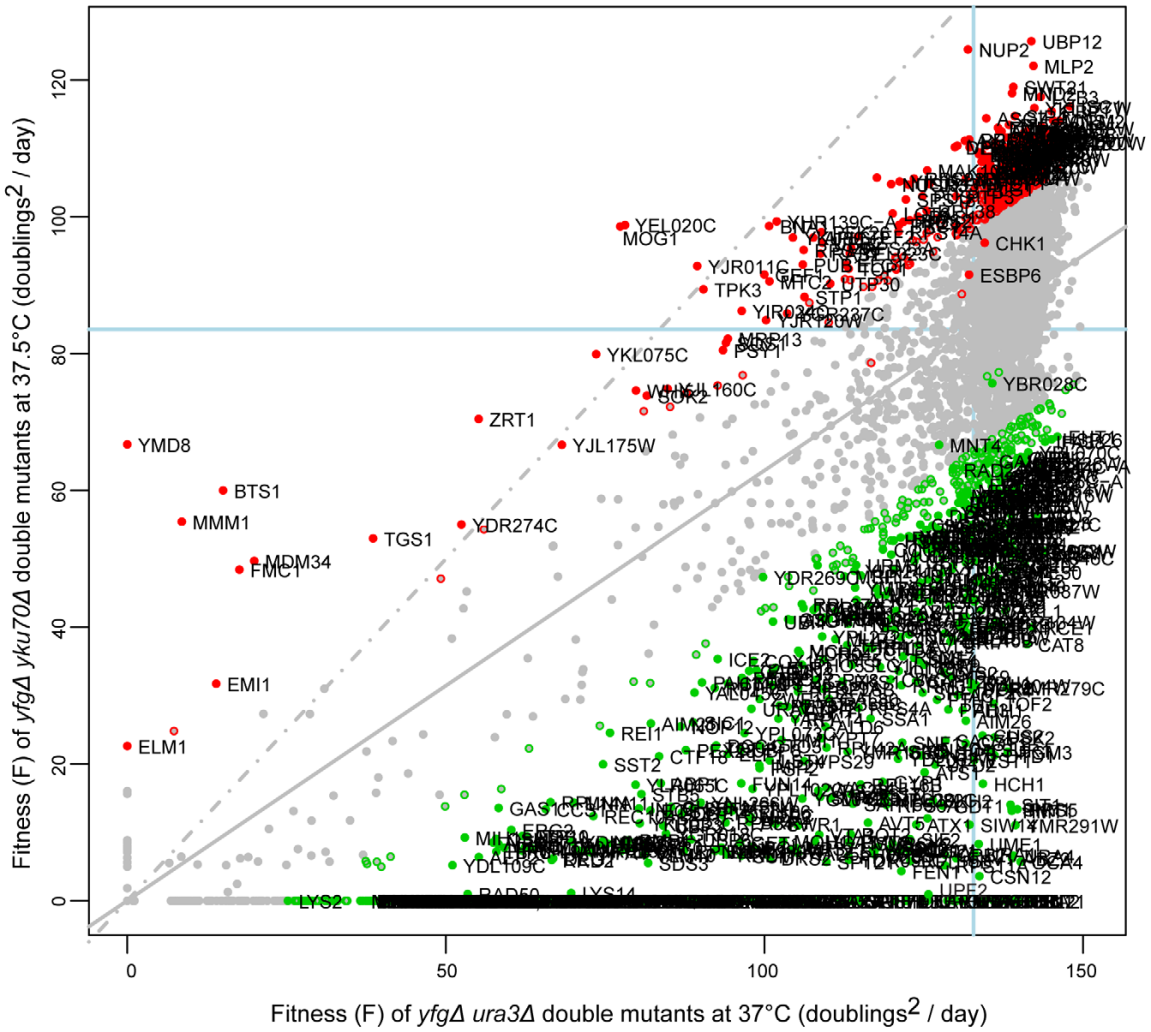
Fitness of *yku70*Δ strains at high temperature. The yeast genome knock out collection was crossed to the *yku70*Δ mutation, or as a control to the *ura3*Δ mutation. 8 replicate crosses were performed and for each, the fitness of all double mutant cultures measured as in Figure 1. Growth of *yku70*Δ *yfg*Δ (“*your favourite gene deletion*”) double mutants was measured at 37.5°C and *ura3*Δ *yfg*Δ strains at 37°C. Gene deletions that significantly enhance (green) or suppress (red) the *yku70*Δ defect, in comparison with the *ura3*Δ mutation are indicated. Those marked by open circles have p-values <0.05 and those filled circles have FDR corrected p-values (q-values) <0.05. The line of equal growth (dashed grey) and a population model of expected fitness (solid grey) are indicated. The average position of *his3*Δ strains are indicated by solid light-blue lines on each axis, as proxy for “wild-type” growth.

QFA of *cdc13-1 yfg*Δ, *yku70*Δ *yfg*Δ and *ura3*Δ *yfg*Δ double mutant libraries was performed at a number of temperatures and therefore a variety of informative comparisons were possible. For example to help identify gene deletions that suppress or enhance the *yku70*Δ temperature dependent growth defect it is useful to compare the fitness of *yku70*Δ *yfg*Δ cells incubated at 37.5°C, with that of control, *ura3*Δ *yfg*Δ, cells incubated at 37°C. In Figure 2, genes which, when deleted, suppress the *yku70*Δ phenotype at 37.5°C will be positioned above the linear regression line and enhancers of *yku70*Δ defects below the line. *yfg*Δ mutations that result in low fitness when combined with the neutral *ura3*Δ mutation will be found on the left and those with high fitness on the right of the x-axis.

The location of each gene in Figure 2 indicates the effect of each deletion on fitness of *yku70*Δ strains versus the effect of the deletion on fitness of *ura3*Δ strains. The regression line drawn through all data points (solid gray line) indicates the expected *yku70*Δ *yfg*Δ fitness given the fitness of the corresponding *ura3*Δ *yfg*Δ mutant. The line of equal growth (dashed gray line) shows the expected positions of *yku70*Δ *yfg*Δ strains if they grew similarly to *ura3*Δ *yfg*Δ strains. Comparing the linear regression with the line of equal growth, it is clear that *yku70*Δ mutants grow poorly relative to control *ura3*Δ mutants, as expected due to the temperature dependent telomere uncapping observed in *yku70*Δ mutants. Figure 2 also highlights large numbers of *yku70*Δ *yfg*Δ strains growing significantly better than expected, given the fitness of the equivalent *ura3*Δ *yfg*Δ mutation at 37°C (red data points, Figure 2) and these *yfg*Δ genes can be classified as *yku70*Δ suppressors. There are also large numbers of *yku70*Δ *yfg*Δ strains that grow worse than expected and these are classified as *yku70*Δ enhancers (green data points, Figure 2). Three further example plots comparing growth of *yfg*Δ *cdc13-1* versus *yfg*Δ *ura3*Δ at 20°C; *yfg*Δ *cdc13-1* versus *yfg*Δ *ura3*Δ at 27°C and *yfg*Δ *ura3*Δ at 37°C versus *yfg*Δ *ura3*Δ at 20°C are shown in Figure S1 and others can be found on our supporting information data files website.

We estimated genetic interaction strength (GIS) as the vertical displacement of each *yku70*Δ *yfg*Δ normalised mutant fitness from the expected normalised fitness, with expected fitness given by a linear regression model (see Text S1, experimental procedures). GIS is dimensionless. This method is equivalent to defining GIS as the deviation of observed fitness from that expected if a multiplicative model of genetic interaction were correct. In all, more than 30,000 genetic interaction strengths, together with their statistical significances, were calculated (Tables S1, S2, S3, S4, S5, S6). Table 1 summarizes the numbers of statistically significant genetic interactions observed under the different conditions of telomere capping. Table 1 clearly illustrates that many more genetic interactions are observed under conditions of mild telomere uncapping (*cdc13-1* strains at 27°C and *yku70*Δ strains at 37.5°C) and that at these temperatures around 5% of gene deletions can show strong suppressing or enhancing interactions (GIS >0.5).

**Table 1:** Percentage of deletions suppressing or enhancing query mutation fitness defects in specific QFA screens.

	Suppressors (%)		Enhancers (%)	
	GIS≥0	GIS≥0.5	GIS≤0	GIS≤−0.5
*cdc13-1* 20°C	1.65	0.07	2.06	0.22
*cdc13-1* 27°C	10.11	4.85	7.15	2.60
*cdc13-1* 36°C	0.53	0.53	0.00	0.00
*yku70Δ* 23°C	1.46	0.07	3.76	0.61
*yku70Δ* 30°C	0.61	0.05	4.07	0.73
*yku70Δ* 37°C	0.92	0.12	7.93	2.52
*yku70Δ* 37.5°C	3.42	0.12	13.19	5.14

We examined the effects of 4,120 gene deletions, ignoring deletions that were technically problematic (e.g. displayed linkage with query mutation, affected uracil, leucine or histidine biosynthesis). The table above shows percentages classified as significant suppressors (FDR corrected q-value <0.05, +ve GIS) or significant enhancers (FDR corrected q-value <0.05, -ve GIS) and with strong interactions (|GIS| ≥0.5).

### Comparing genetic interactions between *cdc13-1* and *yku70*Δ

**Figure 3:**
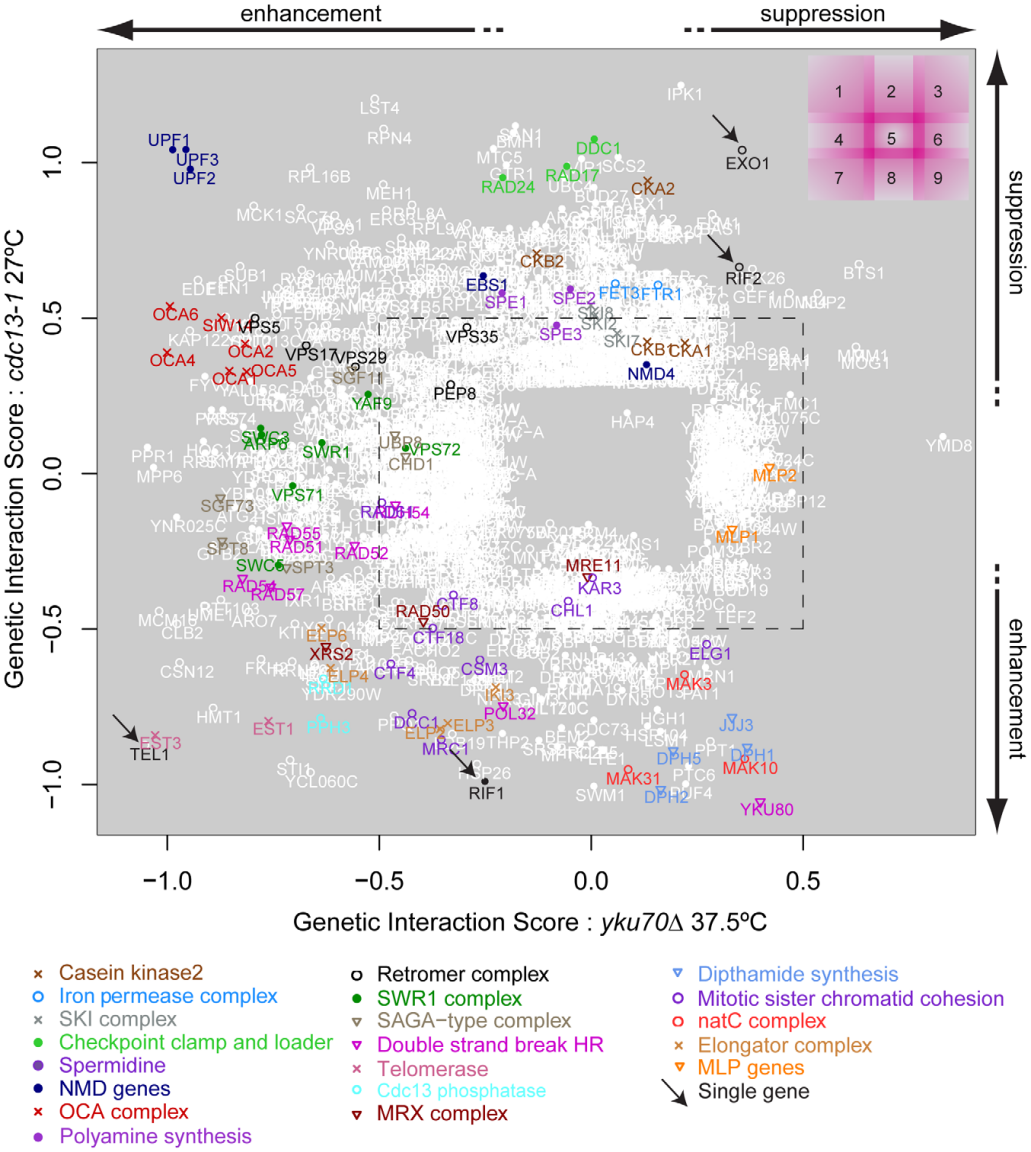
Genetic interaction strength (GIS) comparison between *cdc13-1* and *yku70*Δ. Genes that significantly interacted with *cdc13-1* or *yku70*Δ are shown, most genes did not interact and would be placed in the centre of the plot. Genes encoding components of selected protein complexes (or proteins which work closely together towards the same function) are indicated by colour-co-ordinated text and symbols. Genes that interact with both *cdc13-1* and *yku70*Δ are open white circles, those that interact with just one mutation are filled white circles. Different regions of the plot are indicated on the top right and borders between regions are intentionally blurred/overlapping as there are not precise cut-offs. An arbitrary GIS cutoff of +/−0.5 is indicated by the black dashed rectangle. Also see Figure S3 for further analysis of these data.

In order to compare the effects of gene deletions on cell fitness when combined with *cdc13-1* or *yku70*Δ induced telomere cap defects, it was particularly useful to compare the GIS of each gene with respect to *cdc13-1* or *yku70*Δ after induced telomere uncapping. Figure 3 summarises how different gene deletions interact with the two types of telomere capping defect, suppressing, enhancing or showing no strong interaction with each telomere cap defect. For example, genes that when deleted significantly suppress temperature sensitivity of both *cdc13-1* and *yku70*Δ mutants appear in the top right of this plot (Figure 3, region 3). *EXO1* is in this area as expected because Exo1 generates ssDNA at telomeres in both types of telomere capping mutants (Figure 3, region 2/3, arrow) [7]. Deleting components of the checkpoint sliding clamp (9-1-1 complex) and its clamp loader, suppress *cdc13-1* but have minor effects on growth of *yku70*Δ mutants [7]. *DDC1*, *RAD17* and *RAD24* are in region 2, as expected. *MEC3*, encoding the third component of the sliding clamp was missing from our knock out library and was not tested. Gene deletions that disrupt the telomerase enzyme directly (*est1*Δ, *est3*Δ) enhance the temperature sensitivity of both mutations and so appear in region 7. Genes that, when deleted, suppress *cdc13-1* yet enhance the *yku70*Δ temperature sensitivity (Figure 3, region 1) represent a novel telomere-related phenotype and interestingly include three major components of the nonsense mediated RNA decay pathways (*UPF1, UPF2, UPF3*). It is reassuring that the *UPF* genes cluster so closely in Figure 3 because this strongly suggests that positioning of genes on this plot is an accurate measure of the function of the corresponding gene products in telomere biology.

The position of *YKU80* in the bottom right corner of region 8 is informative. The negative interaction of *yku80*Δ with *cdc13-1* is expected since it is known that *yku80*Δ (and *yku70*Δ) mutations reduce fitness of *cdc13-1* mutants, even at permissive temperatures [8]. However, the positive effect of *yku80*Δ on the growth of *yku70*Δ mutants appears, at first, surprising. The positive epistatic effect simply reflects the fact that *yku70*Δ, *yku80*Δ and *yku70*Δ *yku80*Δ double mutants are all similarly unfit at high temperatures. We have confirmed that in the different W303 genetic background that *yku70*Δ, *yku80*Δ and *yku70*Δ *yku80*Δ double mutants are all similarly unfit at high temperatures. According to the multiplicative model of epistasis the fitness of the *yku70*Δ *yku80*Δ double mutants is significantly higher than expected based on the fitness of the single mutants. Thus, by this criterion, *yku80*Δ suppresses the *yku70*Δ fitness defect. These data can be explained if neither single sub-unit of the Ku comlex retains a telomere capping function in the absence of the other.

It is reasonable to hypothesize, based partly on the behaviour of *UPF1, UPF2* and *UPF3* genes, that genes having similar genetic interactions with *cdc13-1* and *yku70*Δ under particular conditions which are proximal in Figure 3 share similar functions in telomere biology. For example, genes that function similarly to *EXO1* and for example, regulate ssDNA at uncapped telomeres might appear close to *EXO1* in Figure 3. Similarly, genes with strong effects on telomerase function might appear in region 7. Consistent with this hypothesis, it is clear from Figure 3 that many genes encoding members of the same protein complex, or proteins which work together to perform a particular function, often have similar genetic interaction profiles and are located in similar positions on this plot. Examples in Figure 3 include: NMD pathway (*UPF1*, *UPF2*, *UPF3*, region 1); OCA complex (regions 1 & 4); clamp-loader and clamp-like complex (*RAD24*, *DDC1*, *RAD17*, region 2); telomerase (*EST1*, *EST3*, region 7) and dipthamide biosynthesis (*JJJ3*, *DPH1*, *DPH2*, *DPH5*, regions 8 & 9) genes, as well as the numerous other complexes highlighted by the key at the bottom of Figure 3. Table 2 shows the number of genes found in each section of Figure 3. Table 3 lists 19 different sets of genes that are functionally or physically related and that cluster in Figure 3 as well as the single genes *EXO1, RIF1*, *RIF2* and *TEL1* also found in interesting positions. *EXO1* is in its expected position but it is interesting that *RIF1* and *RIF2* are found in different positions in Figure 3, suggesting they have different functions in telomere biology. Further experiments in the W303 genetic background confirm the different interactions of *RIF1* and *RIF2* with *cdc13-1* (Xue, Rushton and Maringele, submitted). *TEL1* encodes the ATM orthologue and is required for telomere maintenance and it clusters very near components of telomerase, in region 7.

**Table 2:** Number and proportion of deletions in each of the nine regions shown in Figure 3.

Region	Number of deletions	% deletions
1	32	0.78
2	34	0.83
3	2	0.05
4	47	1.14
5	70	1.70
6	2	0.05
7	22	0.53
8	25	0.61
9	0	0.00

The number and percentage of gene deletions showing strong genetic interactions (|GIS|≥0.5) in each of the outer regions shown in Figure 3.

**Table 3:** Genes interacting with the telomere cap.

Gene	Comments		Region
*EXO1*		5′ to 3′ exonuclease, degrades uncapped telomeres in *cdc13-1* and *yku70Δ* mutants.	3
*CKA1, CKA2, CKB1, CKB2*		Casein kinase 2, a Ser/Thr protein kinase with roles in cell growth and proliferation; holoenzyme contains Cka1, Cka2, Ckb1 and Ckb2	2
*FET3, FTR1*		Iron permease	2
*SKI2, SKI7, SKI8*		Ski complex component and putative RNA helicase, mediates 3′-5′ RNA degradation by the cytoplasmic exosome;	2
*RAD17, RAD24, DDC1 (MEC3)*		Checkpoint sliding clamp and clamp loader. Active in *cdc13-1* mutants, not in *yku70Δ* mutants, affects nuclease activity	2
*SPE1, SPE2, SPE3*		Spermidine Biosynthesis.	2
*UPF1, UPF2, UPF3* *[EBS1, NMD4]*		ATP-dependent RNA helicase involved in nonsense mediated mRNA decay; required for efficient translation termination at nonsense codons; involved in telomere maintenance	1
*OCA1, OCA2, OCA3, OCA4, OCA5, SIW14*		Putative protein tyrosine phosphatase, required for cell cycle arrest in response to oxidative damage of DNA	1/4
*PEP8, VPS5, VPS17, VPS29, VPS35*		Components of the retromer membrane coat complex, essential for endosome-to-Golgi retrograde protein transport	1/4
*ARP6, SWC3, SWR1, VPS71, VPS72, YAF9*		Components of Swr1 chromatin remodeling complex	4
*CHD1, SGF11, SGF73, SPT3, SPT8, UBP8*		SAGA nucleosome remodeling complex	4
*RAD51, RAD52, RAD54, RAD55, RAD57*		Proteins involved in the repair of double-strand breaks in DNA during vegetative growth and meiosis	4
*EST1, EST3 (EST2)*		Telomerase components	7
*TEL1*		PI3-like protein kinase. ATM orthologue. Required for telomere length maintenance, interacts with MRX.	7
*RRD1, PPH3*		Cdc13, serine 306 phosphatase, that affects *de novo* telomere addition at DSB.	7
*MRE11, RAD50, XRS2*		MRX complex involved in meiosis, telomeres and DSB repair.	7/8
*DPH1, DPH2, DPH5, JJJ3*		Dph1, Dph2, Kti11, Jjj3 and Dph5, for synthesis of diphthamide, a modified histidine residue of translation elongation factor 2 (Eft1 or Eft2);	8/9
*MAK3, MAK10, MAK31*		Catalytic subunit of N-terminal acetyltransferase of the NatC type; required for replication of dsRNA virus	8/9
*CHL1, CTF4, CTF8, CTF18, DCC1, ELG1, KAR3, MRC1, RAD61*		Required for sister chromatid cohesion, replication and/or telomere length maintenance	7/8
*ELP2, ELP3, ELP4, ELP6, IKI3*		Components of elongator complex, required for modification of wobble nucleosides in tRNA; Recently shown to have a role in DNA replication and at telomeres.	8
*RIF1*		Rap1 interacting factor 1, long telomeres.	8
*RIF2*		Rap1 interacting factor 2, long telomeres.	3
*MLP1, MLP2*		Myosin-like proteins associated with the nuclear envelope	6

Genes that affect growth of *cdc13-1* mutants, *yku70*Δ mutants or both. Genes in ( ) brackets are known components of complexes not co-located. This is either because the deletion is missing from our collection or because the gene deletion is in a different position because, for example, deleting the gene affects the function of the adjacent gene also therefore causing a confounding phenotype. Genes in [ ] brackets are associated with the NMD pathway but have different phenotypes and are located in different positions on the plot.

Groupings such as these and their positioning on this type of plot help generate testable, mechanistic predictions about the roles of proteins/protein complexes on telomere capping in budding yeast. For example, we predict that NMD genes (which we examine further in this study) and dipthamide synthesis genes have opposing effects on both Cdc13-mediated and Yku70-mediated telomere capping, because they lie in opposite corners of Figure 3.

The QFA experiments summarised by Figure 3 were performed in a high-throughput manner with the systematic knock out collection in the S288C genetic background and the fitness of different query mutants was measured in slightly different types of media. It was therefore conceivable that some of the genetic interactions scored were due to: defects in the knock out collection, such as incorrect mutations being present or the presence of suppressor mutations, the S288C genetic background, subsets of the cell populations that progressed through the mass mating, sporulation and germination that occur during SGA or media differences.

**Figure 4:**
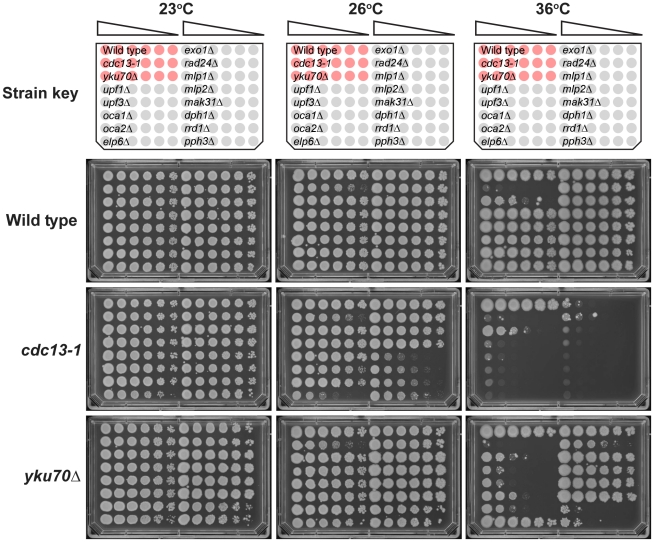
Confirmation of genetic interactions in an alternative genetic background. A selection of genes identified by QFA were combined with either the *yku70*Δ or *cdc13-1* mutations in the W303 genetic background and assessed for growth by manual spot test. Strains were cultured to saturation in 2 ml YPD at 23°C, a six-fold dilution series generated and spotted onto YPD. Strains were incubated at the indicated temperatures for three days before being photographed. All plates contained the reference strains 640 (wild type), 1108 (*cdc13-1*) and 1412 (*yku70*Δ), indicated as red cultures in the key. The “Wild Type” single mutant strains assessed were: 6656, 6811, 3622, 3653, 6620, 1273, 659, 6862, 6927 6951, 6963, 6692 and 6632. The *cdc13-1* double mutant strains assessed were: 6810, 6814, 3624, 3655, 6614, 1296, 1258, 6860, 6928, 6865, 6967, 6694 and 6396. The *yku70*Δ double mutant strains assessed were: 6808, 6812, 4290, 4296, 6628, 1409, 1284, 2413, 2415, 6968, 6971, 6776 and 6763. Growth at other temperatures is shown in Figure S2.

To test whether genetic interactions identified by QFA with *cdc13-1* or *yku70*Δ strains were robust observations we retested a subset of interactions in the W303 genetic background, on rich media, after construction of strains by individual tetrad dissection by manual spot test. Figure 4 and Figure S2 show the behaviour of a number of gene deletions chosen from different regions in Figure 3 to test the effects in W303. In all we measured 26 genetic interactions between 13 gene deletions and *cdc13-1* or *yku70*Δ. Of these we estimate that 20/26 interactions were as expected, 5/26 difficult to classify, and 1/26, due to *elp6*Δ, opposite to that expected after QFA. In particular *exo1*Δ, *mlp1*Δ, *mlp2*Δ, *mak31*Δ and *dph1*Δ mutations suppress the growth defects of *yku70*Δ strains in W303 at 36°C, consistent with their position on the right side of Figure 3 and *exo1*Δ, *rad24*Δ, *upf1*Δ and *upf2*Δ strongly suppress *cdc13-1* at 26°C consistent with their position near the top of Figure 3. *upf1*Δ, *upf2*Δ, *rrd1*Δ and *pph3*Δ mutations all reduced growth of *yku70*Δ strains at 36°C consistent with their position on the left of Figure 3, while *elp6*Δ, *mak31*Δ, *dph2*Δ, *rrd1*Δ and *pph3*Δ mutations all enhanced *cdc13-1* growth defects consistent with their positions near the bottom of Figure 3. Other genes have more subtle effects, the *oca1*Δ and *oca2*Δ mutations had marginal effects on *yku70*Δ strains but improved growth of *cdc13-1* strains (Figure S2). Interestingly the *elp6*Δ mutation enhanced the *cdc13-1* defect at 26°C, as expected, but suppressed the *yku70*Δ strain growth defect at 36°C, the opposite of what was expected from Figure 3. Further experiments will be necessary to clarify the role of Elp6 and other elongator factors in cells with uncapped telomeres. However, overall, it is clear that the majority of genetic interactions identified by QFA are reproducible in smaller scale experiments in a different genetic background.

### Analysis of fitness at other temperatures

Suppressors and enhancers of the *cdc13-1* and *yku70*Δ phenotypes were most easily identified at semi-permissive temperatures for the query mutations (Figure 2, Figure 3), however QFA at other temperatures also proved informative. For example, comparison of the fitness of *yfg*Δ *ura3*Δ strains at 37°C versus 20°C, allowed us to identify temperature sensitive mutants (Figure S1C and Table S9). Of the 57 genes which were categorized with a phenotype of “heat sensitivity: increased” in the *Saccharomyces* Genome Database (http://www.yeastgenome.org), as identified by low though-put experiments, which were also present in the knockout library we used, 45 (79% of total) were identified as being significantly heat sensitive by our independent QFA.

2-dimensional GIS plots, like Figure 3, also proved useful for identifying broader patterns of genetic interactions. For example, we observed a difference between the effects of deleting small and large ribosomal subunit genes on the growth of telomere capping mutants (Figure S3A, S3B). Gene deletions which affect the small ribosomal subunit are generally neutral with both *cdc13-1* and *yku70*Δ mutations (Figure S3A, S3B red). In contrast, disruptions of large ribosomal subunit function suppress the effect of *cdc13-1* on average and enhance that of *yku70*Δ (Figure S3A, S3B blue). Although the basis for this novel observation is unknown it may be related to the finding that the large ribosome sub-unit is subject to autophagy upon starvation, whereas the small ribosome sub-unit is not [23]. Positive and negative regulators of telomere length [24], [25], [26] also showed differing distributions in GIS comparisons – gene deletions which suppress the *yku70*Δ defect are more likely to result in long than short telomeres (Figure S3C). This is perhaps to be expected since *yku70*Δ mutants, on their own, have a short telomere phenotype. Importantly, over 90% of genes identified as suppressors of *cdc13-1* in a previous study [20] showed a positive GIS with *cdc13-1* (Figure S2D), demonstrating that QFA reproduces conclusions derived from qualitatively scored visual inspection. It should be noted however, that the improved sensitivity of QFA has allowed identification of significantly more enhancing mutations than were indentified in the preceding, qualitatively scored study [20].

QFA is sensitive enough to permit identification of genetic interactions even where gene deletions combined with the control *ura3*Δ query mutation impart a poor growth phenotype. For example, deletion of all three *SPE* genes resulted in low fitness that was strongly rescued by *cdc13-1* (Figure S1B, blue, Figure 3 region 2). Interestingly it has recently been reported that increasing spermidine levels increases lifespan in organisms such as yeast, flies and worms [27], but no previous connection with telomeres has been made. Telomere-driven, replicative senescence is thought to be a significant component of the ageing phenotype. Our observations of interactions between *SPE* genes and cells with uncapped telomeres may ultimately lead to experiments to provide insight into the mechanisms by which spermidine affects lifespan.

### NMD and telomere capping

One of the most striking results obtained from QFA experiments was the effect of deleting nonsense mediated RNA decay genes on growth of cells with telomere capping mutations. Deletion of any of the NMD genes *UPF1*, *UPF2* or *UPF3* suppresses the *cdc13-1* telomere capping defect but enhances the *yku70*Δ defect (Figure 3, region 1). We wanted to understand the basis of this interesting interaction and decided to further analyze the NMD genes. We also investigated *EBS1*, a gene that has proposed roles in both the NMD pathway and telomere function [28], [29], [30] and was identified previously as interacting with *CDC13* [20], [31]. *EBS1* had less strong, but qualitatively similar GISs to *UPF* genes in our analysis (Figure 3, region 1∼2), suggesting that the position of *EBS1* in Figure 3 was due a partial defect in nonsense mediated RNA decay.

**Figure 5:**
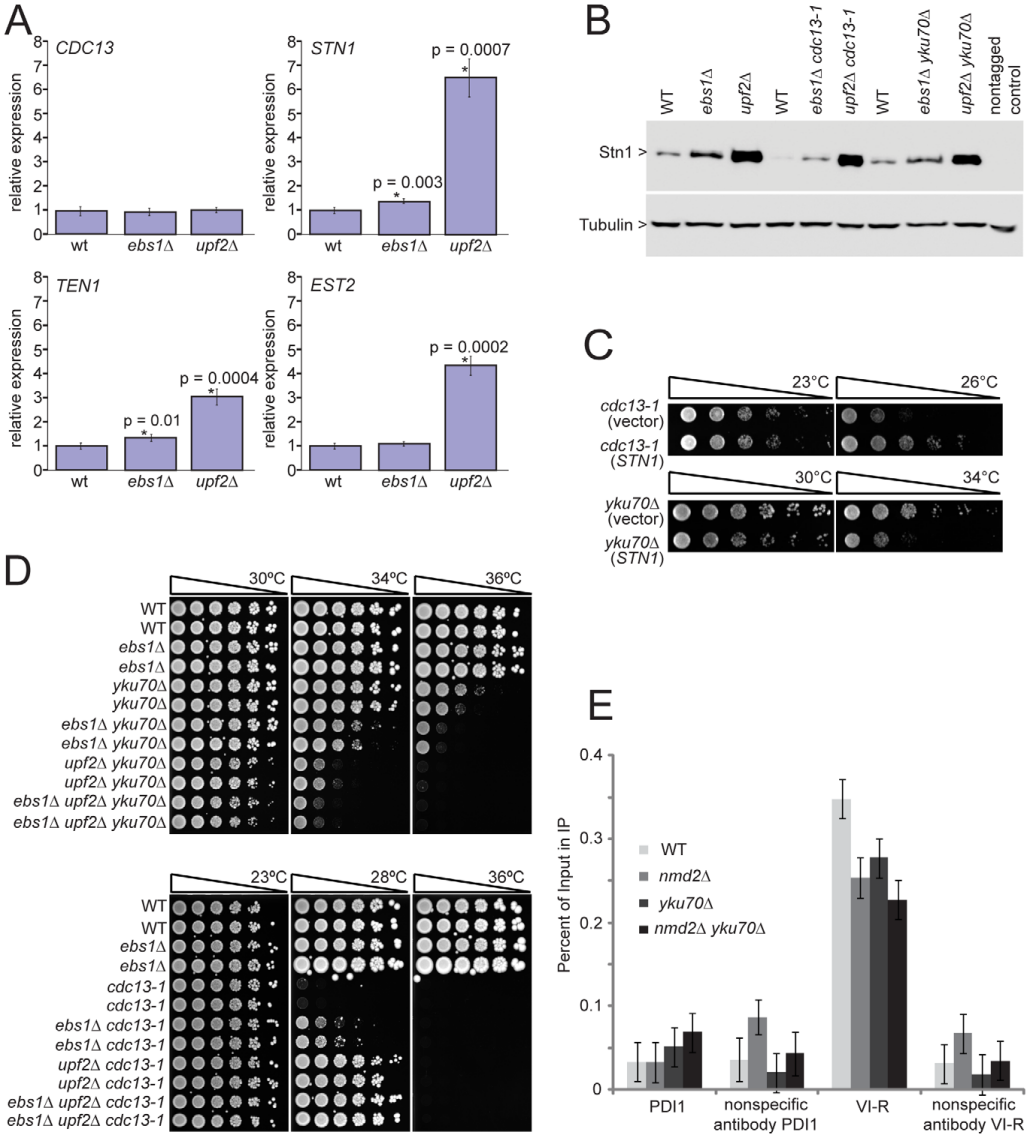
*UPF2* influences telomere capping through *STN1* and telomerase recruitment. A) Transcript levels of four telomere-binding factors measured in *upf2*Δ and *ebs1*Δ mutants. Four strains of each genotype were grown exponentially in liquid culture at 23°C. RNA was isolated and transcript levels were determined by SYBR Green RT-PCR. Each measurement was performed in triplicate and error bars indicate standard deviation from four independent measurements. RNA concentrations of the samples were normalized to the loading control *BUD6*. A single wild type sample was given the value of 1 and all other values were corrected relative to this. Strains measured are 640, 2824, 3001, 4763, 4764, 4765, 4766, 4780, 4781, 4782, 4783 and 4784; B) Western blot analysis of Stn1 protein levels using antibodies against Stn1-13Myc tagged strains. Strains shown are 5757, 5758, 5759, 5760, 5761, 5763, 5764, 5765 and 5766; C) Growth analysis of *yku70*Δ or *cdc13-1* mutants over-expressing *STN1* using the centromeric plasmid pVl1045. The empty vector Ycplac111 was used as a control. Strains 5046, 5047, 5051 and 5052 were spot tested on –LEU medium; D) *upf2*Δ and *ebs1*Δ mutants were combined with *yku70*Δ or *cdc13-1* mutations in the W303 genetic background and assessed for growth by spot test. Strains shown are 640 and 3001 (wild type), 2764 and 2824 (*ebs1*Δ), 2787 and 4309 (*yku70*Δ), 2889 and 2890 (*ebs1*Δ *yku70*Δ), 5007 and 5008 (*nmd2*Δ *yku70*Δ), 5251 and 5242 (*ebs1*Δ *nmd2*Δ *yku70*Δ), 1195 and 4557 (*cdc13-1*), 4576 and 4577 (*ebs1*Δ *cdc13-1*), 4624 and 4625 (*nmd2*Δ *cdc13-1*), 5238 and 5239 (*ebs1*Δ *nmd2*Δ *cdc13-1*); E) ChIP analysis of Est2-13Myc association to the VI-R telomere and the internal locus PDI1 on Chromosome III using primers previously described [50]. Duplicate cultures were grown and harvested in exponential phase. Individual ChIP samples were measured in triplicate and group means are shown with 95% confidence bars derived from a two-way ANOVA. Strains shown are 6977 (Est2-13Myc), 6978 (*nmd2*Δ Est2-13Myc), 6979 (*nmd2*Δ *yku70*Δ Est2-13Myc) and 6980 (*yku70*Δ Est2-13Myc).

One potential mechanism by which *UPF* genes and *EBS1* affect telomere capping is if they regulate the levels of telomere capping proteins. Indeed, *UPF* genes have been shown to regulate transcripts of genes involved in telomere function [32], [33]. The effect of *EBS1* on these transcripts has not so far been reported. Therefore we compared mRNA levels of three NMD targets with roles in telomere regulation (*STN1*, *TEN1* and *EST2*) in *upf2*Δ, *ebs1*Δ and wild-type strains. Transcripts of *STN1* and *TEN1* were increased significantly in *upf2*Δ and *ebs1*Δ, mutants whereas *EST2* transcripts were increased only in *upf2*Δ strains (Figure 5A). We conclude that both *EBS1* and *UPF2* modulate expression of *STN1* and *TEN1*, but the effects of *ebs1*Δ are modest compared to those of *upf2*Δ. Furthermore, elevated levels of Stn1 protein were detected in both *ebs1*Δ and *upf2*Δ mutants (Figure 5B). Consistent with the measured mRNA levels of *STN1*, the increase in Stn1 levels was smaller in *ebs1*Δ strains than *upf2*Δ strains. Thus we concluded that the effects of *UPF2* and *EBS1* could be due to the effects on Stn1 and possibly Ten1 levels.

Increased Stn1 and Ten1 levels are known to suppress the *cdc13-1* defect [33], [34]. To test whether elevated levels of Stn1 or Ten1 proteins could reproduce the enhancement of the *yku70*Δ defect observed in *ebs1*Δ and *upf2*Δ mutants, we over-expressed Stn1 and Ten1 independently of NMD by providing extra copies of *STN1* and *TEN1* on plasmids. Both single copy (centromeric; Figure 5C) and high copy (2 µ) Stn1-expressing plasmids [35] suppressed the temperature sensitivity of *cdc13-1* strains and enhanced the temperature sensitivity of *yku70*Δ strains (Figure S4A), mimicking the *upf2*Δ and *ebs1*Δ phenotypes. In contrast, Ten1-expressing plasmids [35] did not affect the growth of either *cdc13-1* or *yku70*Δ mutants (Holstein; data not shown). We therefore conclude that both *UPF2* and *EBS1* affect telomere capping by modulating expression of *STN1*. However, it is also possible that *UPF2* and *EBS1* affect telomere capping by modulating expression of genes other than *STN1*. To test this and the relative contribution of *STN1* versus any other mechanisms, it would be informative to reduce *STN1* expression in *upf2*Δ mutants. Such experiments might be difficult to perform and interpret since both centromeric single-copy and 2 micron multi-copy *STN1* plasmids suppress the *cdc13-1* defect to similar extents (Figure S4A), suggesting there is not a simple correlation between Stn1 levels and effects on growth of *cdc13-1* and *yku70*Δ mutants.

Since the effect of *ebs1*Δ was milder than that of *upf2*Δ on the fitness of *cdc13-1* and *yku70*Δ cells (Figure 3), we hypothesized that if *ebs1*Δ imparts a mild NMD defect, an *ebs1*Δ *upf2*Δ double mutation would result in the same phenotype as *upf2*Δ on its own. Figure 5D shows that both *upf2*Δ and *ebs1*Δ mutations suppress *cdc13-1* temperature sensitivity and exacerbate *yku70*Δ temperature sensitivity in the W303 genetic background. We also confirmed that *upf1*Δ *upf2*Δ double mutants suppress *cdc13-1* temperature sensitivity and exacerbate *yku70*Δ temperature similarly to either single mutant (Figure S4B). It is clear that the effects of *ebs1*Δ are less strong than *upf2*Δ mutations and interestingly *upf2*Δ *ebs1*Δ double mutants have slightly stronger effects on growth of both *cdc13-1* and *yku70*Δ mutants, suggesting that *ebs1*Δ effects are not solely due to defects in nonsense mediated RNA decay (Figure 5D). We therefore conclude that, at least with respect to telomere capping, *EBS1* and *UPF2* act partially through different pathways. We do not yet understand these differences, but they may be related to the homology between Ebs1 and the telomerase protein Est1.

**Figure 6:**
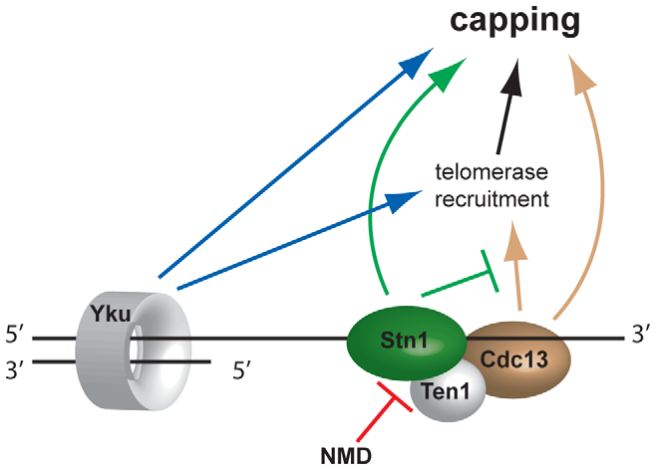
Model of telomere capping activities influenced by NMD. Disruption of NMD activity results in higher Stn1 transcript and protein levels (Figure 5). Stn1 promotes capping directly and is thought to oppose telomerase recruitment through interaction with Cdc13. Since telomerase has telomere capping function, Stn1 therefore both promotes and inhibits telomere capping.

It is simple to hypothesize why increased Stn1 levels, caused by inactivation of nonsense mediated mRNA decay pathways, suppress the *cdc13-1* defect, presumably by stabilizing the Cdc13-1/Stn1/Ten1 complex at telomeres. It is less simple to explain why increased Stn1 levels enhance the *yku70*Δ-induced telomere-capping defect. Our hypothesis is based on the facts that the Stn1 protein can inhibit telomerase activity [36], [37] and that Yku70 interacts with and helps recruit telomerase to telomeres [38], [39]. Thus we hypothesized that *yku70*Δ causes a partial defect in telomerase recruitment, one that is exacerbated by the *upf2*Δ mutation that causes high levels of Stn1, thus inhibiting telomerase activity. To test the simplest version of this hypothesis, that *yku70*Δ and *upf2*Δ mutations reduce the amount of telomerase binding to telomeres, we performed ChIP analyses. We examined binding of the Est2 sub-unit of telomerase in *yku70*Δ, *upf2*Δ and *yku70*Δ *upf2*Δ double mutants. Interestingly we observed a significant reduction in binding of telomerase to telomeres in *yku70*Δ, *upf2*Δ and *yku70*Δ *upf2*Δ mutants (Figure 5E). It is known that *yku70*Δ mutants recruit less telomerase to telomeres [39] but we are unaware of any other reports showing that *upf2*Δ mutants recruit less telomerase to telomeres. This observation most likely explains the short telomere phenotype of *upf2*Δ (as well as *yku70*Δ) mutants [24], [25]. It is noteworthy that although the *upf2*Δ mutation causes a four-fold increase in the *EST2* transcript, it causes a reduction in the amount of Est2 bound to telomeres. This suggests that the increased levels of Stn1in *upf2*Δ cells more than counteracts any mass action effects on telomerase recruitment to telomeres caused by *EST2* over-expression. However, the simple hypothesis that *yku70*Δ *upf2*Δ mutants show a more severe capping defect because of a reduction in the recruitment of telomerase appears not to be valid. Further experiments will be necessary to better understand the complex interplay between Ku, nonsense mediated decay pathways, Cdc13, Stn1 and telomere capping (Figure 6).

## Discussion

Systematic measurement of genetic interactions is a powerful way to help understand how cells and organisms function [40], [41]. This is because genetic approaches examine the role of individual gene products, or individual residues in genes, in the context of the whole organism and can help dissect the effects of weak biochemical interactions that are important for cells to function [42]. Systematic SGA and eMAP experiments typically examine millions of genetic interactions and use comparatively crude measures of growth (colony size) to infer genetic interactions [19], [41]. Here we have more accurately measured a smaller number of genetic interactions, focusing on interactions that affect budding yeast telomere function. The telomere is an important and interesting subject for systematic genetic analysis because it is a complex, subtle and in some senses paradoxical nucleic acid/protein structure that plays critical roles during human ageing and carcinogenesis. One paradox of telomeres is that many DNA damage response proteins, which induce DNA repair or cell cycle arrest when interacting elsewhere in the genome, induce neither response at telomeres but instead play important roles in telomere physiology.

We used Quantitative Fitness Analysis (QFA) to accurately assess the fitness of many thousands of yeast strains containing mutations that affect telomere function in combination with other deletion mutations. To assess fitness, cells were grown in parallel, in 384 spot arrays on solid agar plates. Photographs of plates were taken, images processed and analysed and growth curves for each culture generated. The growth curves are in essence very similar to those observed in liquid culture, with clear exponential and saturation phases (Figure 2 from Lawless et al. 2010) and can be summarized with as few as three logistic growth parameters. The major advantage of QFA over parallel liquid culture methods to measure yeast fitness is that many more cultures can be examined in parallel. For example we routinely follow the growth of about 18,000 parallel cultures (4,500 yeast strains, incubated at four different temperatures), whereas parallel liquid culture based methods are generally restricted to up to 200 parallel cultures [43].

QFA is similar to SGA or EMAP approaches but typically four times fewer strains per plate are cultured (384 spots versus 1536 colonies) [17], [18], [19], [41]. A further difference between QFA and SGA is that in QFA, which has a liquid growth phase, double mutants are cultured for longer before fitness is assessed. This means that that in QFA, synthetically sick double mutants often show poorer growth than is observed in SGA experiments simply because the more divisions that occur the easier it is to observe growth defects. There is a risk with QFA that during the comparatively long culturing period that suppressors or modifiers will arise. In the experiments we performed in this paper the double mutants show conditional, temperature sensitive defects and were generated in permissive conditions where there was little selection for suppressors/modifiers. The principal advantage of QFA over SGA and EMAP is that QFA provides more accurate fitness measurements that can be measured at higher culture densities. The accuracy of QFA is indicated by the tight clustering of genes affecting particular biochemical pathways/functions in Figure 3.

QFA is also lower throughput than “bar code” based assays where up to 6000 independent strains compete in a single culture [44]. One principal difference between QFA and bar code competition methods is that fitness measures are absolute, rather than comparative.

Comparison of genetic interactions observed in yeast cells containing *cdc13-1* or *yku70*Δ mutations, affecting telomeres in different ways, has generated numerous new insights into telomere biology. For example, we have identified at least 19 groups of genes, each representing a particular protein complex or biological process, that significantly affect growth of cells with telomere capping defects in different ways and these are highlighted in Figure 3. Each of these groups of genes, as well as numerous individual genes, warrant further investigation to characterize how they influence the telomere cap. In this paper we followed up just one striking observation that deletions of NMD pathway genes suppress the *cdc13-1* temperature-sensitive phenotype and enhance the *yku70*Δ temperature sensitive phenotype. In *upf2*Δ strains, levels of *STN1* transcripts and levels of Stn1 protein increased. Our detailed follow-up observations are consistent with the hypothesis that the NMD pathway influences Cdc13- or Yku70-mediated telomere capping through modification of Stn1 but not Ten1 levels (Figure 6).

As well as helping generate hypotheses about the roles of individual gene products at telomeres QFA will be ideal for developing, constraining and testing dynamic, systems models of the effects of complex biological processes on telomere function. Any model describing cellular growth and division as an outcome of the complex interaction of gene products e.g. [45] could usefully be parameterized and tested by QFA. We expect QFA to be widely applicable to other quantitative phenotypic screens in budding yeast and other microbial systems.

## Materials and Methods

### Growth media

Library strains created using SGA in this study were cultured in SD/MSG media [17] with appropriate amino-acids and antibiotics added – Canavanine (final concentration, 50 µg/ml); G418 (200 µg/ml); thialysine (50 µg/ml); clonNAT (100 µg/ml); hygromycin B (300 µg/ml). Media were made lacking arginine when using canavanine and lacking lysine when using thialysine. W303 genetic background strains were cultured in YEPD (ade).

### Western blot analysis

Cell lysis and western blot analysis were performed as previously described [46]. Antibody 9E10 from Cancer Research UK was used to detect the C-Myc epitope and anti-tubulin antibodies, from Keith Gull, Oxford, UK, used as loading controls.

### Quantitiative RT-PCR

RNA extraction and RT-PCR were performed as previously described [47]. RNA concentrations of each sample were normalized relative to the loading control, *BUD6*.

### ChIP

Chromatin immunoprecipitation was performed as previously described with minor modifications [48]. Mouse anti-myc (9E10) or goat anti-Mouse antibodies were used for the immunoprecipitations. Immunoprecipitated DNA was quantified by RT-PCR using the SYBR Green qPCR SuperMIX-UDG w/ROX kit (Invitrogen, 11744500).

### Plate filling

Rectangular, single chamber, SBS footprint plates (omnitrays; Nunc, Thermo Fisher Scientific) were filled with 35 ml molten agar media using a Perimatic GP peristaltic pump (Jencons (Scientific) Limited, Leighton Buzzard, UK) fitted with a foot switch. 96-well plates (Greiner Bio-One Ltd.) were filled with liquid media or distilled H_2_O (200 µl per well) using a Wellmate plate-filler with stacker (Matrix Technologies, Thermo Fisher Scientific).

### Robotics

Solid agar to solid agar pinning was performed on a Biomatrix BM3-SC robot (S&P Robotics Inc., Toronto, Canada) using either 384-pin (1 mm diameter) or 1536-pin (0.8 mm diameter) pintools. Inoculation from solid agar to liquid media was performed on the Biomatrix BM3-SC robot using a 96-pin (1 mm diameter) pintool. Resuscitation of frozen strain collections (from liquid to solid agar) was performed on the Biomatrix BM3-SC robot using a 384-pin (1 mm diameter) tool. Re-array procedures were carried out using the BM3-SC robot equipped with a 96-pin rearray pintool. Dilution and spotting of liquid cultures onto solid agar plates was performed on a Biomek FX robot (Beckman Coulter (UK) Limited, High Wycombe, UK) equipped with a pintool magnetic mount and a 96-pin (2 mm diameter) pintool (V&P Scientific, Inc., San Diego, CA, USA). Both the Biomatrix BM3-SC and the Biomek FX were equipped with bar-code readers (Microscan Systems, Inc.) and the bar-codes of plates involved in each experiment were recorded in robot log-files.

### Strains, strain collections, oligonucleotide primers, and plasmids

All strains, strain collections oligonucleotide primers and plasmids are described in Text S1. Single gene deletion collections (a gift from C. Boone) were stored at −80°C in 384-well plates (Greiner BioOne) in 15% glycerol and when required, were thawed and pinned onto YEPD + G418 agar. Strains were then routinely pinned onto fresh YEPD + G418 agar plates approximately every two months but were re-pinned from frozen stocks after approximately 6 months. An array containing 6 replicates of 12 telomere-related genes, 14 replicates of *his3*Δ and 6 replicates of 37 randomly chosen genes was created from the original deletion collection (SGAv2). This array (plate 15 in our deletion mutant collections) was designed to quickly check that gene deletions with familiar phenotypes were behaving as expected and to also provide high numbers of replicates for a small number of genes (49) allowing more robust statistical analysis. This collection was SGAv2p15. Collection SGAv3 was made by re-arraying each of the 15 plates of SGAv2p15, randomly, with the exceptions that all *his3*Δ strains on the plate periphery [17] were not moved and genes which were in the corner area of plates in SGAv2p15 were specifically moved to non-corner positions in SGAv3.

### Growth assays

Liquid-to-solid agar 384-format robotic spot tests were performed as follows. Colonies were inoculated from solid agar SGA plates into 96-well plates containing 200 µl appropriately supplemented liquid SD/MSG media in each well. These were grown to saturation (usually three days), without shaking, at 20°C. Cultures were resuspended, diluted approximately 1/100 in 200 µl H_2_O and spotted onto appropriately supplemented solid SD/MSG media plates which were incubated at different temperatures.

### SGA with *cdc13-1* and *yku70*Δ

SGA query strains DLY5688 (*cdc13-1* flanked by *LEU2* and *HPHMX* (Hygromycin^R^)), DLY3541 (*yku70*Δ*::URA3*) and DLY4228 (*ura3::NATMX*) were crossed to SDLv2p15 and SDLv3 in quadruplicate, giving eight biological replicate crosses each. Fitness of each strain under different conditions was assayed in 384-spot growth assays. As previously [20], growth at 36°C was used as an indication of failure of the SGA process or spontaneous reversion in SGA screens where *cdc13-1* was the query mutation. In this study, repeats with modeled Trimmed Area >25000 after 6 days at 36°C (provided this included no more than 3 repeats for a single gene deletion) were stripped out. In each SGA experiment, a small number of strains were missing from the starting mutant array (due to mis-pinning, strains being lost, replaced etc.). These experiment-specific missing strains; together with genes affecting selection during SGA; and experiment-specific genes situated within 20 kb of SGA markers; were removed from analysis.

### Photography

Solid agar plates were photographed on an spImager (S&P Robotics Inc., Toronto, Canada). The integrated camera (Canon EOS 40D) was used in manual mode with a pre-set manual focus. Manual settings were as follows: exposure, 0.25 s; aperture, F10; white balance, 3700 K; ISO100; image size, large; image quality, fine; image type, .jpg. Using the spImager software, the plate barcode number and a time stamp (date in year, month, day and time in hour, minute, second) were incorporated as the image name (e.g. DLY00000516-2008-12-24_23-59-59.jpg).

### Image analysis

The image analysis tool Colonyzer [21] was used to quantify cell density from captured photographs. Colonyzer corrects for lighting gradients, removing spatial bias from density estimates. It is designed to detect cultures with extremely low cell densities, allowing it to capture a wide range of culture densities after dilute spotting on agar. Colonyzer is available under GPL at http://research.ncl.ac.uk/colonyzer.

### 384 spot versus 1,536 colony sensitivity

We directly compared QFA of pinned 1536- colony format versus spotted 384- culture format and found that the range of normalized 384 spot fitness is approximately 4 times that estimated from 1,536 colony growth curves (Lawless et al., in prep). We also find that 384 spot fitness estimates adequately captures the strong temperature dependent growth of *cdc13-1* mutants, whereas 1536-format growth estimates do not, and that analysis of growth in 384 spot format captures a much higher dynamic range of cell densities than 1536 colony format (approx 1,000 versus 20 fold, see Fig. 2, Lawless et al, 2010). For these reasons we chose to perform QFA of telomere capping mutants arrayed as 384 spotted cultures.

### Sample tracking and data storage

Strain array positions on a 384-spot layout (plate, row, column) were defined in a comma-separated text file and tracked using bar-codes reported in robot log-files. Data was stored in a Robot Object Database (ROD) as described previously [20]. Screen data is exported from ROD in tab delimited format (Table S7) ready for modeling and statistical analysis (see below).

### Modeling of fitness

Culture density (G) was estimated from captured photographs using the Integrated Optical Density (IOD or Trimmed Area; Table S7) measure of cell density provided by the image-analysis tool Colonyzer (Lawless et al 2010). Observed density time series were summarised with the logistic population model, which is an ODE describing self-limiting population growth. It has an analytical solution *G (t)*:
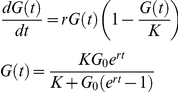



Modelled inoculum density (*G_0_*, AU) was fixed (at 43 AU in this case), assuming that all liquid cultures reached the same density in stationary phase before water dilution and inoculation onto agar. Logistic parameter values *r* (growth rate, d^−1^) and *K* (carrying capacity, AU) were inferred by least squares fit to observations, using optimization routines in the SciPy Python library (code available from http://sourceforge.net/projects/colonyzer/).

For least-squares minimisation, initial guesses for *K* were the maximum observed cell density for that culture. For *r*, we constructed initial guesses by observing that *G'(t)* is at a maximum when t = t^*^:
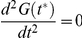



Linearly interpolating between cell density observations we estimated the time of greatest rate of change of density. We then estimated *r* as:
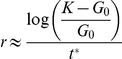



A quantitative measure of fitness was then constructed from the optimal parameters. The particular measure we used was the product of the maximal doubling rate (*MDR*, doublings.d^−1^), which is the inverse of the doubling time and the maximal doubling potential (*MDP*, doublings). These phenotypes were quantified using logistic model parameter estimates as follows.

We estimate the minimum doubling time *T* which the cell population takes to reach a density of *2G_0_* (assuming that the culture is in exponential phase immediately after inoculation):
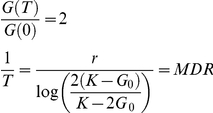



MDP is the number of divisions the culture is observed to undergo. Considering cell growth as a geometric progression:
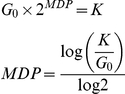



These two phenotypes provide different information about the nature of population fitness and both of them are important, reflecting the rate of growth (*MDR*) and the capacity of the mutant to divide (*MDP*) under given experimental conditions. Our chosen measure of fitness (F = MDR×MDP) places equal importance on these two phenotypes.

### Quantifying genetic interaction

To estimate GIS, F is obtained for a particular temperature for both the QFA screen of interest and a second QFA screen using a control query mutation, *ura3*Δ, which is assumed to be neutral under the experimental conditions, approximating wild-type fitness. Experimental and control strain fitnesses are analysed for evidence of epistatic interactions contradicting a multiplicative model of genetic independence [49] (used due to the ratio scale of the phenotype). We denote the fitness of the query (or background) mutation *F_xyz_*, that of a typical deletion from the yeast knockout library *F_yfgΔ_* and double mutant fitnesses as *F_xyz yfgΔ_*. Genetic independence therefore implies:
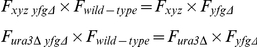
and re-arranging gives:
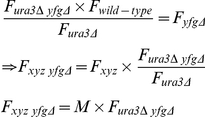
where *M = F_xyz_*/*F_ura3Δ_* is a constant independent of the particular knockout, *yfg*Δ. Thus, after normalising fitnesses (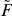) so that the means across all knockouts for both the experimental (QFA, *xyz yfg*Δ) and control (cQFA, *ura3*Δ *yfg*Δ) mutation strains are equal to 1, evidence that 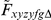 is significantly different from 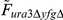 is evidence of genetic interaction. Thus for each knockout a model is fitted of the form: 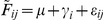
where 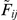 *i* = 1,2, *j* = 1,..,*n_i_* is the *j* ^th^ normalised fitness for treatment *i* (cQFA = 1, QFA = 2), *µ* is the mean fitness for the knockout in the control QFA, *γ*_1_ = 0, *γ*_2_ represents genetic interaction and *ε*_ij_ is (normal, iid) random error. Typically *n_i_* is 8 (4 replicates each of SGAv2p15 and SGAv3), but is sometimes a larger multiple of 8 for strains that are repeated in the libraries (e.g. those on plate 15). The model is fitted in R using the lmList command. For each knockout the fitted value of *γ*_2_ is recorded as an estimated measure of the strength of genetic interaction (with the sign indicating suppression or enhancement) and the corresponding *p*-value is used as a measure of statistical significance of the effect. The *p*-value is corrected using the R function p.adjust to give a FDR-corrected *q*-value, and it is this *q*-value which is thresholded to give the lists of statistically significant genetically interacting strains (see Figure 2).

The R code used for the statistical analysis of data from ROD and Colonyzer is available from the authors on request and sample logistic analysis output is presented in Table S8.

Stringent lists of genetic interactors for each query mutation and growth condition (Tables S1, S2, S3, S4, S5, S6) were compiled by imposing a 5% FDR cutoff and arbitrarily removing genes with −0.5< GIS >0.5.

### Supplementary information data files website

Raw output data and hyperlinked supplementary tables, together with detailed legends for interpretation of data files are available from: http://research.ncl.ac.uk/colonyzer/AddinallQFA/

## Supporting Information

Figure S1 Fitness plots for *cdc13-1 versus ura3*Δ strains at 20°C and 27°C and for *ura3*Δ strains at 20°C versus 37°C. A] Fitness plot showing *cdc13-1* at 20°C, compared with QFA for *ura3*Δ at 20°C. B] Fitness plot showing QFA for of *cdc13-1* at 27°C compared with QFA for *ura3*Δ at 27°C. Note that *SPE1*, *SPE2* and *SPE3* (blue text and symbols) have poor fitness in both conditions but fall above the line of equal growth, hence double mutants with *cdc13-1* grow better than the single deletion strains. Note the tight clustering of the members of the *MRX* complex: *MRE11*, *RAD50* and *XRS2* (blue squares) C] Temperature sensitivity analysis of *ura3*Δ strains comparing fitnesses of *ura3*Δ mutations at 37°C with those at 20°C. A list of stringent temperature sensitive deletion mutations taken from this analysis are presented in Table S9. Figure annotations are as for Figure 2.
(1.27 MB TIF)

Figure S2 W303 Spot tests. Strains shown in Figure 4 were incubated at the additional temperatures shown.
(2.28 MB TIF)

Figure S3 Genetic interaction strength (GIS) analysis of ribosomal and telomere length maintenance genes. A] Large ribosomal subunit genes [26] (blue) and small ribosomal subunit genes [26] (red) are indicated. B] Contour lines represent the density of large (blue) and small (red) ribosomal subunit genes and all other genes (white). Density was estimated using the kde2d function in the R package MASS (bandwidth  =  0.6). Crosses represent the mean location for genes in each group. See also Figure S3. C] Genes identified as affecting telomere length maintenance [24]–[26] are indicated. Colour represents telomere length, ranging from blue (short telomeres) to red (long telomeres). White indicates telomere length was either wild-type or not measured [24]. D] Genes that were previously identified [20] as suppressors of *cdc13-1* (red) are indicated.
(2.22 MB TIF)

Figure S4 Effects of over-expression of *STN1* or Nonsense Mediated Decay genes on telomere capping mutants. A] Spot tests of *yku70*Δ (4413) or *cdc13-1* (1195) mutants over-expressing *STN1* using the centromeric vector pVL1045 and the 2 µ vector pVL1066. The empty centromeric vector Ycplac111 or the 2 µ vector YEplac181 were used as controls. Strains were grown on selective media at the temperatures indicated. B] Spot tests of strains on YEPD at the temperatures indicated. Strains were 2787, 4557, 6656, 4765, 6976, 6808, 5007, 6974, 6975, 6810, 5107, 6867 and 6868.
(1.07 MB TIF)

Table S1 List of suppressors and enhancers of *yku70*Δ defect at 23°C. A list of genes which, when deleted, result in suppression or enhancement of the *yku70*Δ phenotype at 23°C. Only included are gene deletions which passed a 5% FDR cutoff and had a GIS of greater than 0.5 (+ or −) in magnitude. http://research.ncl.ac.uk/colonyzer/AddinallQFA/S1_yku70_23.html. See http://research.ncl.ac.uk/colonyzer/AddinallQFA for a list of all significant interactors, a GIS plot showing interactors and raw data.
(0.01 MB HTML)

Table S2 List of suppressors and enhancers of *yku70*Δ defect at 30°C. A list of genes which, when deleted, result in suppression or enhancement of the *yku70*Δ phenotype at 30°C. Only included are gene deletions which passed a 5% FDR cutoff and had a GIS of greater than 0.5 (+ or −) in magnitude. http://research.ncl.ac.uk/colonyzer/AddinallQFA/S2_yku70_30.html. See http://research.ncl.ac.uk/colonyzer/AddinallQFA for a list of all significant interactors, a GIS plot showing interactors and raw data.
(0.02 MB HTML)

Table S3 List of suppressors and enhancers of *yku70*Δ defect at 37°C. A list of genes which, when deleted, result in suppression or enhancement of the *yku70*Δ phenotype at 37°C. Only included are gene deletions which passed a 5% FDR cutoff and had a GIS of greater than 0.5 (+ or −) in magnitude. http://research.ncl.ac.uk/colonyzer/AddinallQFA/S3_yku70_37.html. See http://research.ncl.ac.uk/colonyzer/AddinallQFA for a list of all significant interactors, a GIS plot showing interactors and raw data.
(0.05 MB HTML)

Table S4 List of suppressors and enhancers of *yku70*Δ defect at 37.5°C. A list of genes which, when deleted, result in suppression or enhancement of the *yku70*Δ phenotype at 37.5°C. Only included are gene deletions which passed a 5% FDR cutoff and had a GIS of greater than 0.5 (+ or −) in magnitude. http://research.ncl.ac.uk/colonyzer/AddinallQFA/S4_yku70_375.html. See http://research.ncl.ac.uk/colonyzer/AddinallQFA for a list of all significant interactors, a GIS plot showing interactors and raw data.
(0.10 MB HTML)

Table S5 List of suppressors and enhancers of *cdc13-1* defect at 20°C. A list of genes which, when deleted, result in suppression or enhancement of the *cdc13-1* phenotype at 20°C. Only included are gene deletions which passed a 5% FDR cutoff and had a GIS of greater than 0.5 (+ or −) in magnitude. http://research.ncl.ac.uk/colonyzer/AddinallQFA/S5_cdc131_20.html. See http://research.ncl.ac.uk/colonyzer/AddinallQFA for a list of all significant interactors, a GIS plot showing interactors and raw data.
(0.01 MB HTML)

Table S6 List of suppressors and enhancers of *cdc13-1* defect at 27°C. A list of genes which, when deleted, result in suppression or enhancement of the *cdc13-1* phenotype at 27°C. Only included are gene deletions which passed a 5% FDR cutoff and had a GIS of greater than 0.5 (+ or −) in magnitude. http://research.ncl.ac.uk/colonyzer/AddinallQFA/S6_cdc131_27.html. See http://research.ncl.ac.uk/colonyzer/AddinallQFA for a list of all significant interactors, a GIS plot showing interactors and raw data.
(0.14 MB HTML)

Table S7 ROD output. Robot log files, metadata and image analysis data are stored in the ROD database, then exported in this format for further analysis. These are text files compressed in. zip format: http://research.ncl.ac.uk/colonyzer/AddinallQFA/RODOutput.zip. See http://research.ncl.ac.uk/colonyzer/AddinallQFA for detailed description of column contents.
(131.84 MB ZIP)

Table S8 Logistic data file. ROD output data is subjected to logistic modelling and exported in this format for further analysis. These are text files compressed in .zip format: http://research.ncl.ac.uk/colonyzer/AddinallQFA/Logistic.zip. See http://research.ncl.ac.uk/colonyzer/AddinallQFA for detailed description of column contents.
(30.13 MB ZIP)

Table S9 List of suppressors and enhancers of temperature-induced fitness defect at 37°C. A list of genes which, when deleted, result in significantly better or worse growth at 37°C compared to 20°C. Only included are gene deletions which passed a 5% FDR cutoff and had a GIS of greater than 0.5 (+ or −) in magnitude. http://research.ncl.ac.uk/colonyzer/AddinallQFA/S9_cSGA_37_20.html. See http://research.ncl.ac.uk/colonyzer/AddinallQFA for a list of all significant interactors, a GIS plot of these data and raw data.
(0.09 MB HTML)

Text S1 Supplemental experimental procedures, strains and strain collections list, supplemental references.
(0.12 MB PDF)

## References

[1] Olovnikov AM (1973). A theory of marginotomy. The incomplete copying of template margin in enzymic synthesis of polynucleotides and biological significance of the phenomenon.. J Theor Biol.

[2] Longhese MP (2008). DNA damage response at functional and dysfunctional telomeres.. Genes Dev.

[3] Greider CW, Blackburn EH (1985). Identification of a specific telomere terminal transferase activity in *Tetrahymena* extracts.. Cell.

[4] Lydall D (2009). Taming the tiger by the tail: modulation of DNA damage responses by telomeres.. EMBO J.

[5] Fisher TS, Zakian VA (2005). Ku: a multifunctional protein involved in telomere maintenance.. DNA Repair (Amst).

[6] Boulton SJ, Jackson SP (1998). Components of the Ku-dependent non-homologous end-joining pathway are involved in telomeric length maintenance and telomeric silencing.. EMBO J.

[7] Maringele L, Lydall D (2002). *EXO1*-dependent single-stranded DNA at telomeres activates subsets of DNA damage and spindle checkpoint pathways in budding yeast *yku70*Delta mutants.. Genes Dev.

[8] Polotnianka RM, Li J, Lustig AJ (1998). The yeast Ku heterodimer is essential for protection of the telomere against nucleolytic and recombinational activities.. Curr Biol.

[9] Gravel S, Larrivee M, Labrecque P, Wellinger RJ (1998). Yeast Ku as a regulator of chromosomal DNA end structure.. Science.

[10] Miyake Y, Nakamura M, Nabetani A, Shimamura S, Tamura M (2009). RPA-like mammalian Ctc1-Stn1-Ten1 complex binds to single-stranded DNA and protects telomeres independently of the Pot1 pathway.. Mol Cell.

[11] Surovtseva YV, Churikov D, Boltz KA, Song X, Lamb JC (2009). Conserved telomere maintenance component 1 interacts with STN1 and maintains chromosome ends in higher eukaryotes.. Mol Cell.

[12] Lin JJ, Zakian VA (1996). The Saccharomyces CDC13 protein is a single-strand TG1-3 telomeric DNA-binding protein in vitro that affects telomere behavior in vivo.. Proc Natl Acad Sci U S A.

[13] Nugent CI, Hughes TR, Lue NF, Lundblad V (1996). Cdc13p: a single-strand telomeric DNA-binding protein with a dual role in yeast telomere maintenance.. Science.

[14] Garvik B, Carson M, Hartwell L (1995). Single-stranded DNA arising at telomeres in *cdc13* mutants may constitute a specific signal for the *RAD9* checkpoint.. Mol Cell Biol.

[15] Zubko MK, Guillard S, Lydall D (2004). Exo1 and Rad24 differentially regulate generation of ssDNA at telomeres of *Saccharomyces cerevisiae cdc13-1* mutants.. Genetics.

[16] Weinert TA, Kiser GL, Hartwell LH (1994). Mitotic checkpoint genes in budding yeast and the dependence of mitosis on DNA replication and repair.. Genes Dev.

[17] Tong AH, Boone C (2006). Synthetic genetic array analysis in *Saccharomyces cerevisiae*.. Methods Mol Biol.

[18] Tong AH, Evangelista M, Parsons AB, Xu H, Bader GD (2001). Systematic genetic analysis with ordered arrays of yeast deletion mutants.. Science.

[19] Collins SR, Miller KM, Maas NL, Roguev A, Fillingham J (2007). Functional dissection of protein complexes involved in yeast chromosome biology using a genetic interaction map.. Nature.

[20] Addinall SG, Downey M, Yu M, Zubko MK, Dewar J (2008). A genomewide suppressor and enhancer analysis of cdc13-1 reveals varied cellular processes influencing telomere capping in Saccharomyces cerevisiae.. Genetics.

[21] Lawless C, Wilkinson D, Young A, Addinall S, Lydall D (2010). Colonyzer: automated quantification of micro-organism growth characteristics on solid agar.. BMC Bioinformatics.

[22] Shah NA, Laws RJ, Wardman B, Zhao LP, Hartman JLt (2007). Accurate, precise modeling of cell proliferation kinetics from time-lapse imaging and automated image analysis of agar yeast culture arrays.. BMC Syst Biol.

[23] Kraft C, Deplazes A, Sohrmann M, Peter M (2008). Mature ribosomes are selectively degraded upon starvation by an autophagy pathway requiring the Ubp3p/Bre5p ubiquitin protease.. Nat Cell Biol.

[24] Askree SH, Yehuda T, Smolikov S, Gurevich R, Hawk J (2004). A genome-wide screen for *Saccharomyces cerevisiae* deletion mutants that affect telomere length.. Proc Natl Acad Sci U S A.

[25] Gatbonton T, Imbesi M, Nelson M, Akey JM, Ruderfer DM (2006). Telomere length as a quantitative trait: genome-wide survey and genetic mapping of telomere length-control genes in yeast.. PLoS Genet.

[26] SGD (2008). Saccharomyces Genome Database.

[27] Eisenberg T, Knauer H, Schauer A, Buttner S, Ruckenstuhl C (2009). Induction of autophagy by spermidine promotes longevity.. Nat Cell Biol.

[28] Ford AS, Guan Q, Neeno-Eckwall E, Culbertson MR (2006). Ebs1p, a negative regulator of gene expression controlled by the Upf proteins in the yeast Saccharomyces cerevisiae.. Eukaryot Cell.

[29] Luke B, Azzalin CM, Hug N, Deplazes A, Peter M (2007). Saccharomyces cerevisiae Ebs1p is a putative ortholog of human Smg7 and promotes nonsense-mediated mRNA decay.. Nucleic Acids Res.

[30] Zhou J, Hidaka K, Futcher B (2000). The Est1 subunit of yeast telomerase binds the Tlc1 telomerase RNA.. Mol Cell Biol.

[31] Downey M, Houlsworth R, Maringele L, Rollie A, Brehme M (2006). A genome-wide screen identifies the evolutionarily conserved KEOPS complex as a telomere regulator.. Cell.

[32] Dahlseid JN, Lew-Smith J, Lelivelt MJ, Enomoto S, Ford A (2003). mRNAs encoding telomerase components and regulators are controlled by UPF genes in *Saccharomyces cerevisiae*.. Eukaryot Cell.

[33] Enomoto S, Glowczewski L, Lew-Smith J, Berman JG (2004). Telomere cap components influence the rate of senescence in telomerase-deficient yeast cells.. Mol Cell Biol.

[34] Grandin N, Damon C, Charbonneau M (2000). Cdc13 cooperates with the yeast Ku proteins and Stn1 to regulate telomerase recruitment.. Mol Cell Biol.

[35] Petreaca RC, Chiu HC, Nugent CI (2007). The role of Stn1p in Saccharomyces cerevisiae telomere capping can be separated from its interaction with Cdc13p.. Genetics.

[36] Puglisi A, Bianchi A, Lemmens L, Damay P, Shore D (2008). Distinct roles for yeast Stn1 in telomere capping and telomerase inhibition.. EMBO J.

[37] Chandra A, Hughes TR, Nugent CI, Lundblad V (2001). Cdc13 both positively and negatively regulates telomere replication.. Genes Dev.

[38] Stellwagen AE, Haimberger ZW, Veatch JR, Gottschling DE (2003). Ku interacts with telomerase RNA to promote telomere addition at native and broken chromosome ends.. Genes Dev.

[39] Chan A, Boule JB, Zakian VA (2008). Two pathways recruit telomerase to Saccharomyces cerevisiae telomeres.. PLoS Genet.

[40] Beltrao P, Cagney G, Krogan NJ (2010). Quantitative genetic interactions reveal biological modularity.. Cell.

[41] Costanzo M, Baryshnikova A, Bellay J, Kim Y, Spear ED (2010). The genetic landscape of a cell.. Science.

[42] Gibson TJ (2009). Cell regulation: determined to signal discrete cooperation.. Trends Biochem Sci.

[43] Warringer J, Ericson E, Fernandez L, Nerman O, Blomberg A (2003). High-resolution yeast phenomics resolves different physiological features in the saline response.. Proc Natl Acad Sci U S A.

[44] Hillenmeyer ME, Fung E, Wildenhain J, Pierce SE, Hoon S (2008). The chemical genomic portrait of yeast: uncovering a phenotype for all genes.. Science.

[45] Smallbone K, Simeonidis E, Swainston N, Mendes P (2010). Towards a genome-scale kinetic model of cellular metabolism.. BMC Syst Biol.

[46] Morin I, Ngo HP, Greenall A, Zubko MK, Morrice N (2008). Checkpoint-dependent phosphorylation of Exo1 modulates the DNA damage response.. EMBO J.

[47] Greenall A, Lei G, Swan DC, James K, Wang L (2008). A genome wide analysis of the response to uncapped telomeres in budding yeast reveals a novel role for the NAD+ biosynthetic gene BNA2 in chromosome end protection.. Genome Biol.

[48] Aparicio O, Geisberg JV, Struhl K (2004). Chromatin immunoprecipitation for determining the association of proteins with specific genomic sequences in vivo.. Curr Protoc Cell Biol Chapter 17.

[49] Mani R, St Onge RP, Hartman JLt, Giaever G, Roth FP (2008). Defining genetic interaction.. Proc Natl Acad Sci U S A.

[50] Bianchi A, Shore D (2007). Early replication of short telomeres in budding yeast.. Cell.

