# Supplementary figures and images for: Quantitative Fitness Analysis Shows That NMD Proteins and Many Other Protein Complexes Suppress or Enhance Distinct Telomere Cap Defects
Source: PLoS Genet. 2011 Apr 7;7(4):e1001362. doi: 10.1371/journal.pgen.1001362 (PMC3072368; doi:10.1371/journal.pgen.1001362)

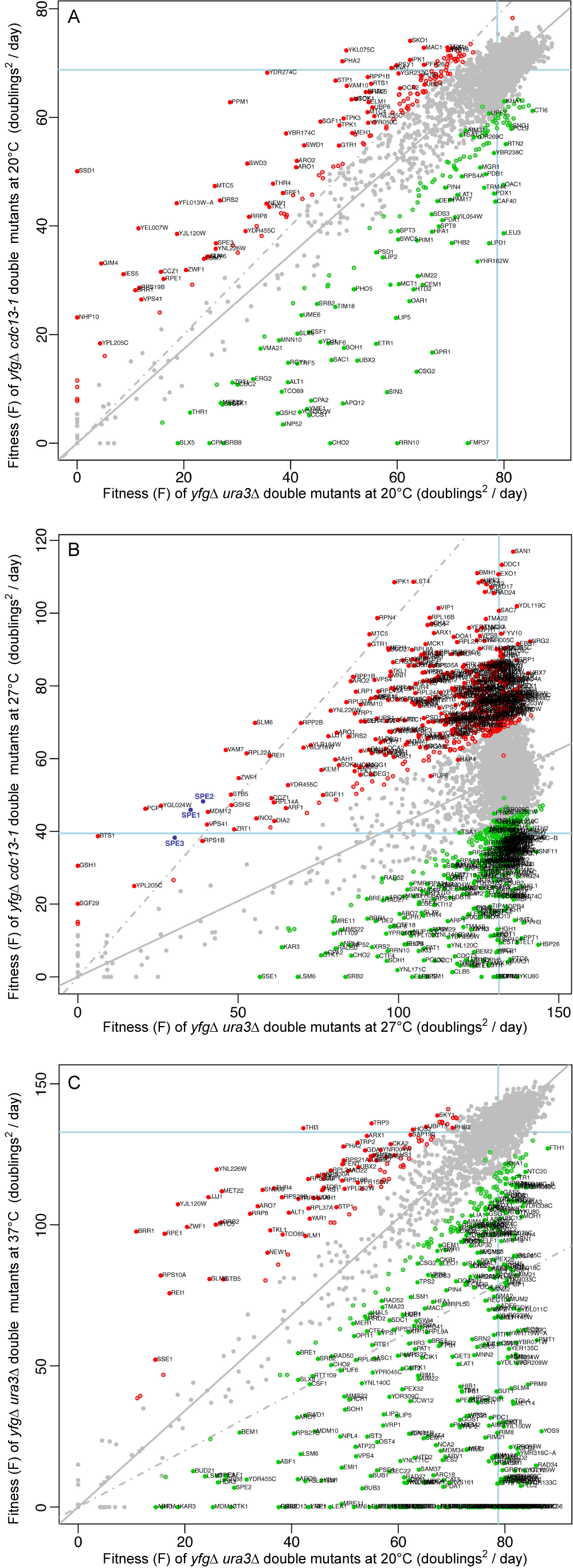

Supplement: Figure S1 — Fitness plots for cdc13-1 versus ura3Δ strains at 20°C and 27°C and for ura3Δ strains at 20°C versus 37°C. A] Fitness plot showing cdc13-1 at 20°C, compared with QFA for ura3Δ at 20°C. B] Fitness plot showing QFA for of cdc13-1 at 27°C compared with QFA for ura3Δ at 27°C. Note that SPE1, SPE2 and SPE3 (blue text and symbols) have poor fitness in both conditions but fall above the line of equal growth, hence double mutants with cdc13-1 grow better than the single deletion strains. Note the tight clustering of the members of the MRX complex: MRE11, RAD50 and XRS2 (blue squares) C] Temperature sensitivity analysis of ura3Δ strains comparing fitnesses of ura3Δ mutations at 37°C with those at 20°C. A list of stringent temperature sensitive deletion mutations taken from this analysis are presented in Table S9. Figure annotations are as for Figure 2. (1.27 MB TIF) [file pgen.1001362.s001.tif]

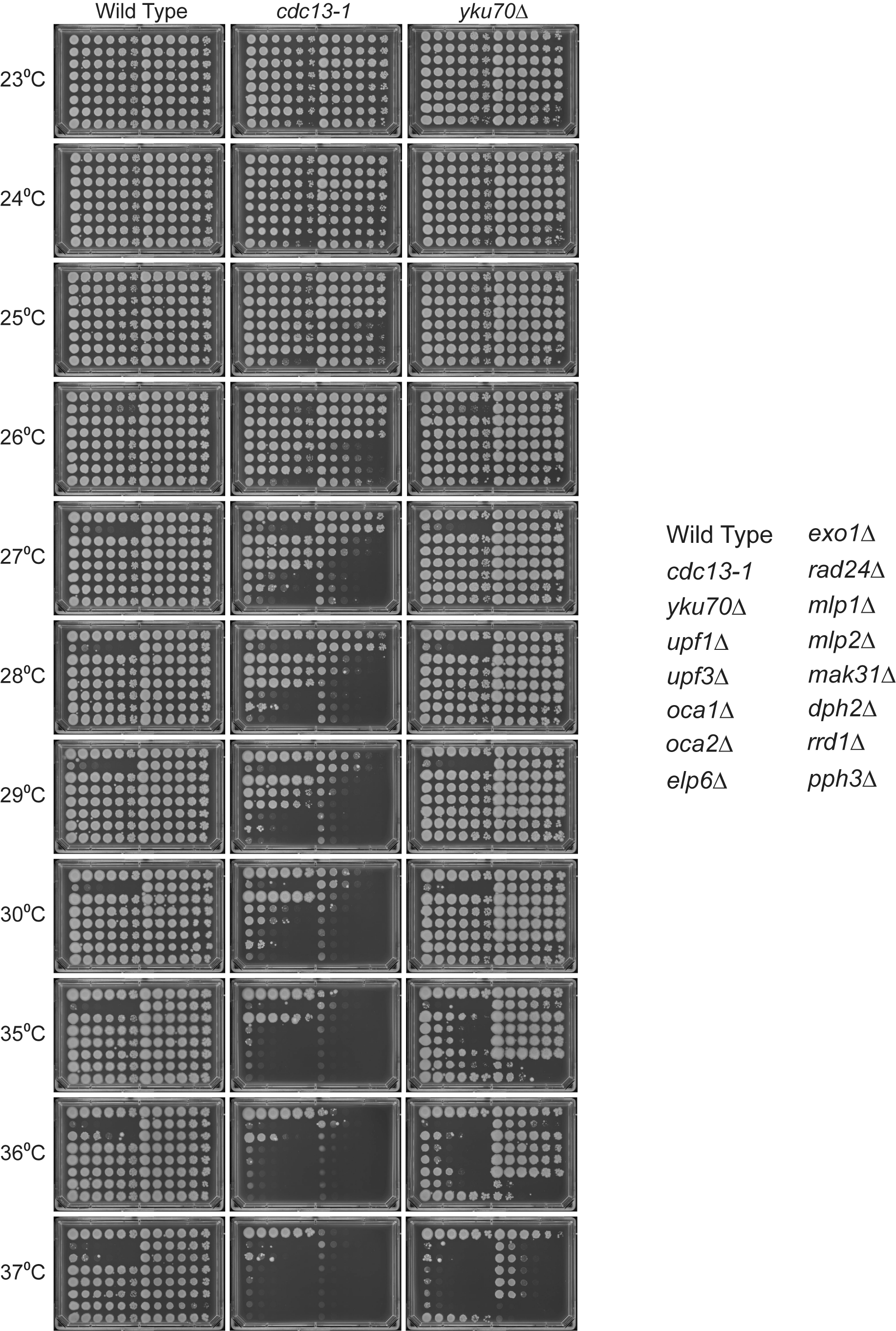

Supplement: Figure S2 — W303 Spot tests. Strains shown in Figure 4 were incubated at the additional temperatures shown. (2.28 MB TIF) [file pgen.1001362.s002.tif]

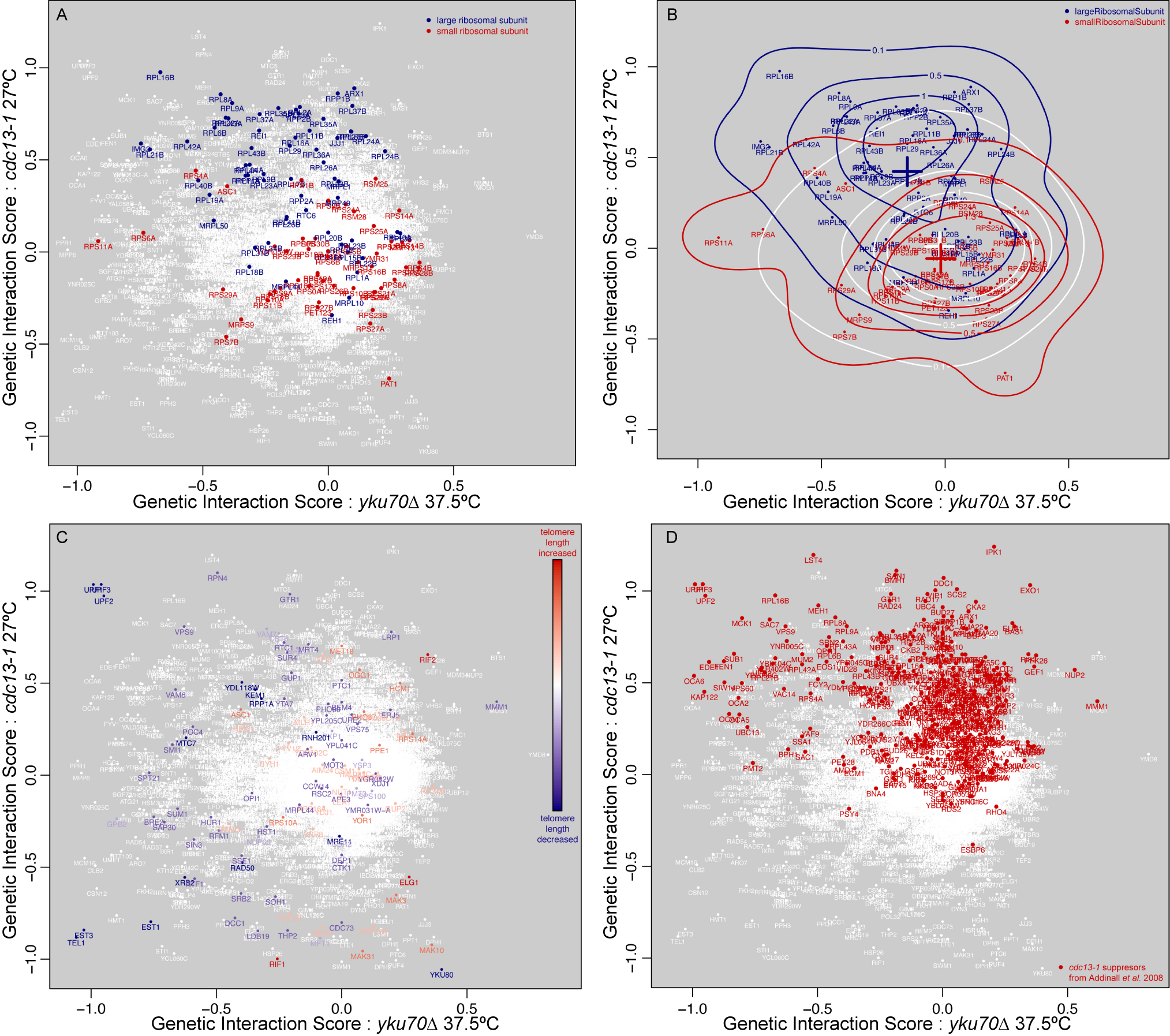

Supplement: Figure S3 — Genetic interaction strength (GIS) analysis of ribosomal and telomere length maintenance genes. A] Large ribosomal subunit genes [26] (blue) and small ribosomal subunit genes [26] (red) are indicated. B] Contour lines represent the density of large (blue) and small (red) ribosomal subunit genes and all other genes (white). Density was estimated using the kde2d function in the R package MASS (bandwidth = 0.6). Crosses represent the mean location for genes in each group. See also Figure S3. C] Genes identified as affecting telomere length maintenance [24]–[26] are indicated. Colour represents telomere length, ranging from blue (short telomeres) to red (long telomeres). White indicates telomere length was either wild-type or not measured [24]. D] Genes that were previously identified [20] as suppressors of cdc13-1 (red) are indicated. (2.22 MB TIF) [file pgen.1001362.s003.tif]

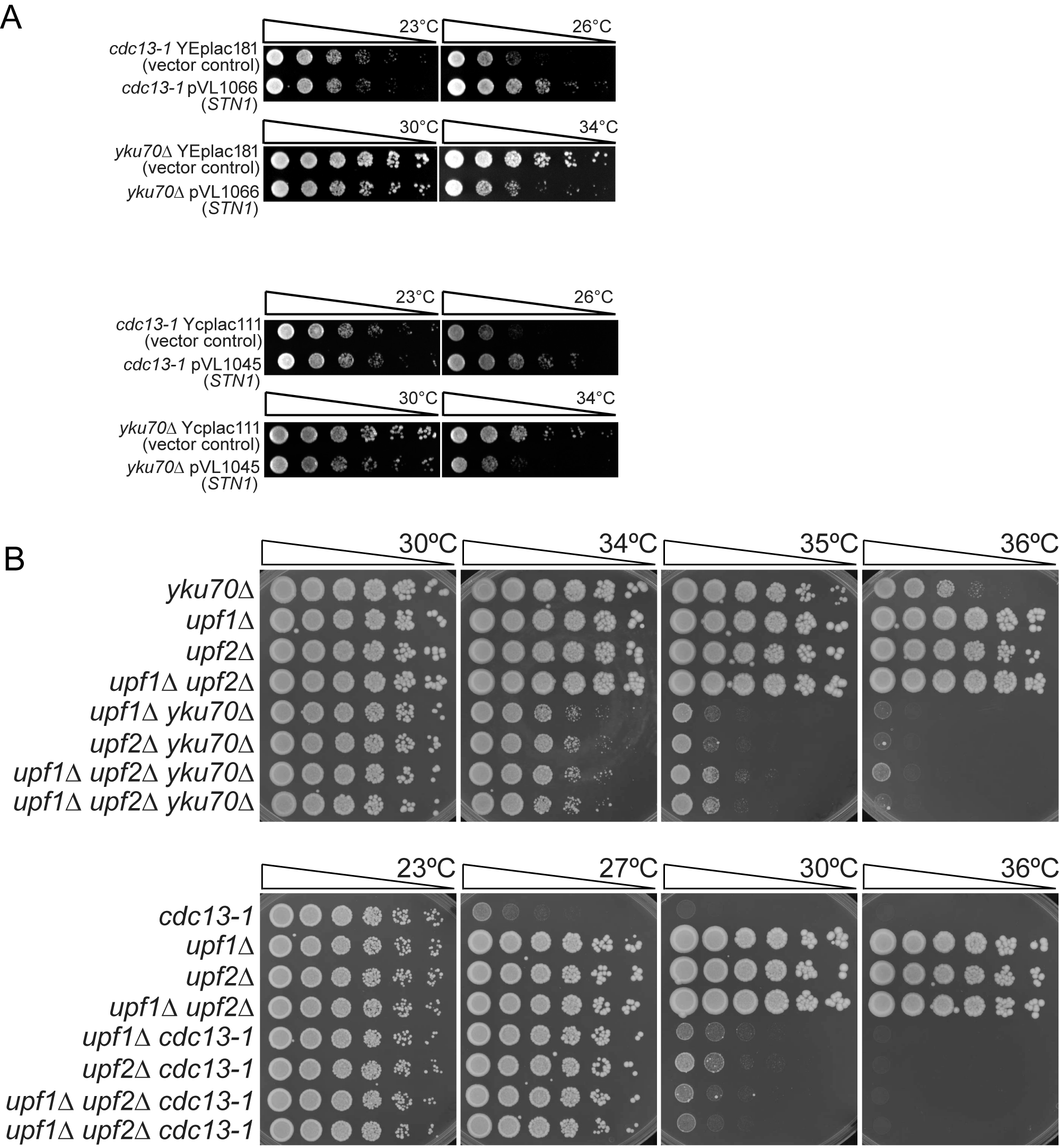

Supplement: Figure S4 — Effects of over-expression of STN1 or Nonsense Mediated Decay genes on telomere capping mutants. A] Spot tests of yku70Δ (4413) or cdc13-1 (1195) mutants over-expressing STN1 using the centromeric vector pVL1045 and the 2 µ vector pVL1066. The empty centromeric vector Ycplac111 or the 2 µ vector YEplac181 were used as controls. Strains were grown on selective media at the temperatures indicated. B] Spot tests of strains on YEPD at the temperatures indicated. Strains were 2787, 4557, 6656, 4765, 6976, 6808, 5007, 6974, 6975, 6810, 5107, 6867 and 6868. (1.07 MB TIF) [file pgen.1001362.s004.tif]
