# Supplementary material for: Quantitative Fitness Analysis Shows That NMD Proteins and Many Other Protein Complexes Suppress or Enhance Distinct Telomere Cap Defects
Source: PLoS Genet. 2011 Apr 7;7(4):e1001362. doi: 10.1371/journal.pgen.1001362 (PMC3072368; doi:10.1371/journal.pgen.1001362)
Supplement: Table S1 — List of suppressors and enhancers of yku70Δ defect at 23°C. A list of genes which, when deleted, result in suppression or enhancement of the yku70Δ phenotype at 23°C. Only included are gene deletions which passed a 5% FDR cutoff and had a GIS of greater than 0.5 (+ or −) in magnitude. http://research.ncl.ac.uk/colonyzer/AddinallQFA/S1_yku70_23.html. See http://research.ncl.ac.uk/colonyzer/AddinallQFA for a list of all significant interactors, a GIS plot showing interactors and raw data. (0.01 MB HTML) [file pgen.1001362.s005.html]

Genetic interaction hitlist after QFA

yku70Δ at 23° C

| | ORF | GIS | stderr | tval | pval | qval | genename | interaction | query | | --- | --- | --- | --- | --- | --- | --- | --- | --- | | YLR014C | -0.9802 | 0.06795 | -14.420 | 0.000e+00 | 0.000e+00 | PPR1 | Phenotypic enhancement | yku70 | | YIL009C-A | -0.9585 | 0.06795 | -14.100 | 0.000e+00 | 0.000e+00 | EST3 | Phenotypic enhancement | yku70 | | YCR071C | -0.8079 | 0.06795 | -11.890 | 0.000e+00 | 0.000e+00 | IMG2 | Phenotypic enhancement | yku70 | | YBL025W | -0.7705 | 0.06795 | -11.340 | 0.000e+00 | 0.000e+00 | RRN10 | Phenotypic enhancement | yku70 | | YML107C | -0.7598 | 0.06795 | -11.180 | 0.000e+00 | 0.000e+00 | PML39 | Phenotypic enhancement | yku70 | | YBR036C | -0.7514 | 0.06795 | -11.060 | 0.000e+00 | 0.000e+00 | CSG2 | Phenotypic enhancement | yku70 | | YLR233C | -0.7324 | 0.02568 | -28.520 | 0.000e+00 | 0.000e+00 | EST1 | Phenotypic enhancement | yku70 | | YDR369C | -0.7170 | 0.06795 | -10.550 | 0.000e+00 | 0.000e+00 | XRS2 | Phenotypic enhancement | yku70 | | YGL168W | -0.6834 | 0.06795 | -10.060 | 0.000e+00 | 0.000e+00 | HUR1 | Phenotypic enhancement | yku70 | | YMR274C | -0.6481 | 0.06795 | -9.537 | 0.000e+00 | 0.000e+00 | RCE1 | Phenotypic enhancement | yku70 | | YIL040W | -0.6396 | 0.06795 | -9.413 | 0.000e+00 | 0.000e+00 | APQ12 | Phenotypic enhancement | yku70 | | YMR224C | -0.6341 | 0.02568 | -24.690 | 0.000e+00 | 0.000e+00 | MRE11 | Phenotypic enhancement | yku70 | | YKL212W | -0.6124 | 0.06795 | -9.013 | 0.000e+00 | 0.000e+00 | SAC1 | Phenotypic enhancement | yku70 | | YGL167C | -0.6107 | 0.06795 | -8.987 | 0.000e+00 | 0.000e+00 | PMR1 | Phenotypic enhancement | yku70 | | YGR157W | -0.6094 | 0.06795 | -8.968 | 0.000e+00 | 0.000e+00 | CHO2 | Phenotypic enhancement | yku70 | | YOL004W | -0.5840 | 0.06795 | -8.595 | 0.000e+00 | 0.000e+00 | SIN3 | Phenotypic enhancement | yku70 | | YNL250W | -0.5824 | 0.02774 | -20.990 | 0.000e+00 | 0.000e+00 | RAD50 | Phenotypic enhancement | yku70 | | YHR041C | -0.5775 | 0.06795 | -8.499 | 0.000e+00 | 0.000e+00 | SRB2 | Phenotypic enhancement | yku70 | | YIR023W | -0.5742 | 0.06795 | -8.450 | 0.000e+00 | 0.000e+00 | DAL81 | Phenotypic enhancement | yku70 | | YJR109C | -0.5634 | 0.04805 | -11.730 | 0.000e+00 | 0.000e+00 | CPA2 | Phenotypic enhancement | yku70 | | YBR170C | -0.5387 | 0.06795 | -7.928 | 2.220e-15 | 1.724e-13 | NPL4 | Phenotypic enhancement | yku70 | | YOR302W | -0.5361 | 0.06795 | -7.889 | 3.109e-15 | 2.329e-13 | \_ | Phenotypic enhancement | yku70 | | YOR106W | -0.5224 | 0.06795 | -7.688 | 1.510e-14 | 1.057e-12 | VAM3 | Phenotypic enhancement | yku70 | | YLR085C | -0.5118 | 0.06795 | -7.532 | 5.063e-14 | 3.432e-12 | ARP6 | Phenotypic enhancement | yku70 | | YLR449W | -0.5072 | 0.06795 | -7.463 | 8.527e-14 | 5.435e-12 | FPR4 | Phenotypic enhancement | yku70 | | YPR057W | 0.6384 | 0.06795 | 9.394 | 0.000e+00 | 0.000e+00 | BRR1 | Phenotypic suppression | yku70 | | YML038C | 0.6794 | 0.06795 | 9.998 | 0.000e+00 | 0.000e+00 | YMD8 | Phenotypic suppression | yku70 | | YGL255W | 0.7084 | 0.06795 | 10.430 | 0.000e+00 | 0.000e+00 | ZRT1 | Phenotypic suppression | yku70 | |
