# Supplementary material for: Quantitative Fitness Analysis Shows That NMD Proteins and Many Other Protein Complexes Suppress or Enhance Distinct Telomere Cap Defects
Source: PLoS Genet. 2011 Apr 7;7(4):e1001362. doi: 10.1371/journal.pgen.1001362 (PMC3072368; doi:10.1371/journal.pgen.1001362)
Supplement: Table S2 — List of suppressors and enhancers of yku70Δ defect at 30°C. A list of genes which, when deleted, result in suppression or enhancement of the yku70Δ phenotype at 30°C. Only included are gene deletions which passed a 5% FDR cutoff and had a GIS of greater than 0.5 (+ or −) in magnitude. http://research.ncl.ac.uk/colonyzer/AddinallQFA/S2_yku70_30.html. See http://research.ncl.ac.uk/colonyzer/AddinallQFA for a list of all significant interactors, a GIS plot showing interactors and raw data. (0.02 MB HTML) [file pgen.1001362.s006.html]

Genetic interaction hitlist after QFA

yku70Δ at 30° C

| | ORF | GIS | stderr | tval | pval | qval | genename | interaction | query | | --- | --- | --- | --- | --- | --- | --- | --- | --- | | YLR014C | -0.9798 | 0.06647 | -14.740 | 0.000e+00 | 0.000e+00 | PPR1 | Phenotypic enhancement | yku70 | | YIL009C-A | -0.9515 | 0.06647 | -14.310 | 0.000e+00 | 0.000e+00 | EST3 | Phenotypic enhancement | yku70 | | YCR071C | -0.8007 | 0.06647 | -12.050 | 0.000e+00 | 0.000e+00 | IMG2 | Phenotypic enhancement | yku70 | | YLR233C | -0.7559 | 0.02512 | -30.090 | 0.000e+00 | 0.000e+00 | EST1 | Phenotypic enhancement | yku70 | | YGL168W | -0.7414 | 0.06647 | -11.150 | 0.000e+00 | 0.000e+00 | HUR1 | Phenotypic enhancement | yku70 | | YIL040W | -0.7409 | 0.06647 | -11.150 | 0.000e+00 | 0.000e+00 | APQ12 | Phenotypic enhancement | yku70 | | YDR369C | -0.7044 | 0.06647 | -10.600 | 0.000e+00 | 0.000e+00 | XRS2 | Phenotypic enhancement | yku70 | | YBL025W | -0.6841 | 0.06647 | -10.290 | 0.000e+00 | 0.000e+00 | RRN10 | Phenotypic enhancement | yku70 | | YKL212W | -0.6805 | 0.06647 | -10.240 | 0.000e+00 | 0.000e+00 | SAC1 | Phenotypic enhancement | yku70 | | YNL106C | -0.6520 | 0.06647 | -9.809 | 0.000e+00 | 0.000e+00 | INP52 | Phenotypic enhancement | yku70 | | YBR036C | -0.6441 | 0.06647 | -9.690 | 0.000e+00 | 0.000e+00 | CSG2 | Phenotypic enhancement | yku70 | | YHR041C | -0.6408 | 0.06647 | -9.640 | 0.000e+00 | 0.000e+00 | SRB2 | Phenotypic enhancement | yku70 | | YMR274C | -0.6353 | 0.06647 | -9.558 | 0.000e+00 | 0.000e+00 | RCE1 | Phenotypic enhancement | yku70 | | YJL204C | -0.6301 | 0.06647 | -9.479 | 0.000e+00 | 0.000e+00 | RCY1 | Phenotypic enhancement | yku70 | | YGL167C | -0.6249 | 0.06647 | -9.402 | 0.000e+00 | 0.000e+00 | PMR1 | Phenotypic enhancement | yku70 | | YML107C | -0.6191 | 0.06647 | -9.314 | 0.000e+00 | 0.000e+00 | PML39 | Phenotypic enhancement | yku70 | | YMR224C | -0.6037 | 0.02512 | -24.030 | 0.000e+00 | 0.000e+00 | MRE11 | Phenotypic enhancement | yku70 | | YPR024W | -0.5982 | 0.06647 | -8.999 | 0.000e+00 | 0.000e+00 | YME1 | Phenotypic enhancement | yku70 | | YOR302W | -0.5969 | 0.06647 | -8.980 | 0.000e+00 | 0.000e+00 | \_ | Phenotypic enhancement | yku70 | | YJR109C | -0.5943 | 0.04700 | -12.640 | 0.000e+00 | 0.000e+00 | CPA2 | Phenotypic enhancement | yku70 | | YNL250W | -0.5884 | 0.02714 | -21.680 | 0.000e+00 | 0.000e+00 | RAD50 | Phenotypic enhancement | yku70 | | YGR157W | -0.5558 | 0.06647 | -8.361 | 0.000e+00 | 0.000e+00 | CHO2 | Phenotypic enhancement | yku70 | | YBR170C | -0.5474 | 0.06647 | -8.235 | 2.220e-16 | 1.664e-14 | NPL4 | Phenotypic enhancement | yku70 | | YIR023W | -0.5417 | 0.06647 | -8.149 | 4.441e-16 | 3.215e-14 | DAL81 | Phenotypic enhancement | yku70 | | YNR006W | -0.5410 | 0.06647 | -8.140 | 4.441e-16 | 3.215e-14 | VPS27 | Phenotypic enhancement | yku70 | | YLR418C | -0.5381 | 0.06647 | -8.095 | 6.661e-16 | 4.742e-14 | CDC73 | Phenotypic enhancement | yku70 | | YDR207C | -0.5340 | 0.06647 | -8.033 | 8.882e-16 | 6.219e-14 | UME6 | Phenotypic enhancement | yku70 | | YLR449W | -0.5218 | 0.06647 | -7.851 | 4.219e-15 | 2.906e-13 | FPR4 | Phenotypic enhancement | yku70 | | YMR272C | -0.5090 | 0.06647 | -7.658 | 1.910e-14 | 1.217e-12 | SCS7 | Phenotypic enhancement | yku70 | | YDR359C | -0.5039 | 0.06647 | -7.580 | 3.486e-14 | 2.127e-12 | EAF1 | Phenotypic enhancement | yku70 | | YGL255W | 0.7024 | 0.06647 | 10.570 | 0.000e+00 | 0.000e+00 | ZRT1 | Phenotypic suppression | yku70 | | YML038C | 0.7579 | 0.06647 | 11.400 | 0.000e+00 | 0.000e+00 | YMD8 | Phenotypic suppression | yku70 | |
