# Supplementary material for: Quantitative Fitness Analysis Shows That NMD Proteins and Many Other Protein Complexes Suppress or Enhance Distinct Telomere Cap Defects
Source: PLoS Genet. 2011 Apr 7;7(4):e1001362. doi: 10.1371/journal.pgen.1001362 (PMC3072368; doi:10.1371/journal.pgen.1001362)
Supplement: Table S3 — List of suppressors and enhancers of yku70Δ defect at 37°C. A list of genes which, when deleted, result in suppression or enhancement of the yku70Δ phenotype at 37°C. Only included are gene deletions which passed a 5% FDR cutoff and had a GIS of greater than 0.5 (+ or −) in magnitude. http://research.ncl.ac.uk/colonyzer/AddinallQFA/S3_yku70_37.html. See http://research.ncl.ac.uk/colonyzer/AddinallQFA for a list of all significant interactors, a GIS plot showing interactors and raw data. (0.05 MB HTML) [file pgen.1001362.s007.html]

Genetic interaction hitlist after QFA

yku70Δ at 37° C

| | ORF | GIS | stderr | tval | pval | qval | genename | interaction | query | | --- | --- | --- | --- | --- | --- | --- | --- | --- | | YLR014C | -1.0540 | 0.09549 | -11.040 | 0.000e+00 | 0.000e+00 | PPR1 | Phenotypic enhancement | yku70 | | YIL009C-A | -1.0300 | 0.09549 | -10.790 | 0.000e+00 | 0.000e+00 | EST3 | Phenotypic enhancement | yku70 | | YPR046W | -1.0270 | 0.09549 | -10.750 | 0.000e+00 | 0.000e+00 | MCM16 | Phenotypic enhancement | yku70 | | YJR084W | -1.0230 | 0.09549 | -10.710 | 0.000e+00 | 0.000e+00 | CSN12 | Phenotypic enhancement | yku70 | | YGL016W | -0.9543 | 0.09549 | -9.994 | 0.000e+00 | 0.000e+00 | KAP122 | Phenotypic enhancement | yku70 | | YPR119W | -0.9453 | 0.03609 | -26.190 | 0.000e+00 | 0.000e+00 | CLB2 | Phenotypic enhancement | yku70 | | YDR067C | -0.9295 | 0.09549 | -9.734 | 0.000e+00 | 0.000e+00 | OCA6 | Phenotypic enhancement | yku70 | | YNR025C | -0.8955 | 0.09549 | -9.378 | 0.000e+00 | 0.000e+00 | \_ | Phenotypic enhancement | yku70 | | YBL088C | -0.8780 | 0.09549 | -9.194 | 0.000e+00 | 0.000e+00 | TEL1 | Phenotypic enhancement | yku70 | | YGL066W | -0.8756 | 0.09549 | -9.170 | 0.000e+00 | 0.000e+00 | SGF73 | Phenotypic enhancement | yku70 | | YKR035C | -0.8698 | 0.09549 | -9.109 | 0.000e+00 | 0.000e+00 | OPI8 | Phenotypic enhancement | yku70 | | YNR024W | -0.8526 | 0.09549 | -8.929 | 0.000e+00 | 0.000e+00 | MPP6 | Phenotypic enhancement | yku70 | | YDR025W | -0.8252 | 0.09549 | -8.642 | 0.000e+00 | 0.000e+00 | RPS11A | Phenotypic enhancement | yku70 | | YCR095C | -0.8211 | 0.09549 | -8.599 | 0.000e+00 | 0.000e+00 | OCA4 | Phenotypic enhancement | yku70 | | YPR060C | -0.8154 | 0.09549 | -8.539 | 0.000e+00 | 0.000e+00 | ARO7 | Phenotypic enhancement | yku70 | | YMR216C | -0.8080 | 0.09549 | -8.461 | 0.000e+00 | 0.000e+00 | SKY1 | Phenotypic enhancement | yku70 | | YPL138C | -0.7905 | 0.09549 | -8.278 | 2.220e-16 | 2.271e-14 | SPP1 | Phenotypic enhancement | yku70 | | YNL099C | -0.7817 | 0.09549 | -8.186 | 2.220e-16 | 2.271e-14 | OCA1 | Phenotypic enhancement | yku70 | | YLR233C | -0.7774 | 0.03609 | -21.540 | 0.000e+00 | 0.000e+00 | EST1 | Phenotypic enhancement | yku70 | | YCR034W | -0.7740 | 0.09549 | -8.105 | 4.441e-16 | 4.433e-14 | FEN1 | Phenotypic enhancement | yku70 | | YNL032W | -0.7579 | 0.09549 | -7.937 | 2.220e-15 | 2.069e-13 | SIW14 | Phenotypic enhancement | yku70 | | YLR015W | -0.7507 | 0.09549 | -7.862 | 3.775e-15 | 3.368e-13 | BRE2 | Phenotypic enhancement | yku70 | | YCR071C | -0.7474 | 0.09549 | -7.827 | 5.107e-15 | 4.461e-13 | IMG2 | Phenotypic enhancement | yku70 | | YDR120C | -0.7404 | 0.09549 | -7.754 | 9.104e-15 | 7.790e-13 | TRM1 | Phenotypic enhancement | yku70 | | YJR118C | -0.7347 | 0.09549 | -7.694 | 1.443e-14 | 1.210e-12 | ILM1 | Phenotypic enhancement | yku70 | | YOR069W | -0.7323 | 0.09549 | -7.668 | 1.754e-14 | 1.442e-12 | VPS5 | Phenotypic enhancement | yku70 | | YHR077C | -0.7282 | 0.03609 | -20.180 | 0.000e+00 | 0.000e+00 | UPF2 | Phenotypic enhancement | yku70 | | YDR392W | -0.7200 | 0.09549 | -7.540 | 4.752e-14 | 3.832e-12 | SPT3 | Phenotypic enhancement | yku70 | | YLR085C | -0.7164 | 0.09549 | -7.503 | 6.328e-14 | 5.006e-12 | ARP6 | Phenotypic enhancement | yku70 | | YNL068C | -0.7026 | 0.09549 | -7.358 | 1.892e-13 | 1.344e-11 | FKH2 | Phenotypic enhancement | yku70 | | YLR055C | -0.6984 | 0.09549 | -7.314 | 2.629e-13 | 1.837e-11 | SPT8 | Phenotypic enhancement | yku70 | | YPL090C | -0.6956 | 0.09549 | -7.285 | 3.260e-13 | 2.241e-11 | RPS6A | Phenotypic enhancement | yku70 | | YMR080C | -0.6943 | 0.09549 | -7.271 | 3.608e-13 | 2.440e-11 | UPF1 | Phenotypic enhancement | yku70 | | YHL029C | -0.6917 | 0.09549 | -7.244 | 4.405e-13 | 2.888e-11 | OCA5 | Phenotypic enhancement | yku70 | | YGL163C | -0.6917 | 0.09549 | -7.244 | 4.408e-13 | 2.888e-11 | RAD54 | Phenotypic enhancement | yku70 | | YBL047C | -0.6820 | 0.09549 | -7.142 | 9.293e-13 | 5.904e-11 | EDE1 | Phenotypic enhancement | yku70 | | YGR229C | -0.6762 | 0.09549 | -7.082 | 1.440e-12 | 8.879e-11 | SMI1 | Phenotypic enhancement | yku70 | | YBR231C | -0.6740 | 0.09549 | -7.058 | 1.707e-12 | 1.037e-10 | SWC5 | Phenotypic enhancement | yku70 | | YLR449W | -0.6734 | 0.09549 | -7.052 | 1.775e-12 | 1.063e-10 | FPR4 | Phenotypic enhancement | yku70 | | YPL100W | -0.6700 | 0.09549 | -7.016 | 2.303e-12 | 1.360e-10 | ATG21 | Phenotypic enhancement | yku70 | | YGR072W | -0.6663 | 0.09549 | -6.978 | 3.023e-12 | 1.760e-10 | UPF3 | Phenotypic enhancement | yku70 | | YNL056W | -0.6646 | 0.09549 | -6.960 | 3.435e-12 | 1.973e-10 | OCA2 | Phenotypic enhancement | yku70 | | YDL077C | -0.6636 | 0.09549 | -6.949 | 3.711e-12 | 2.103e-10 | VAM6 | Phenotypic enhancement | yku70 | | YOR054C | -0.6617 | 0.09549 | -6.930 | 4.251e-12 | 2.376e-10 | VHS3 | Phenotypic enhancement | yku70 | | YPL139C | -0.6600 | 0.09549 | -6.912 | 4.833e-12 | 2.659e-10 | UME1 | Phenotypic enhancement | yku70 | | YBR036C | -0.6599 | 0.09549 | -6.910 | 4.882e-12 | 2.659e-10 | CSG2 | Phenotypic enhancement | yku70 | | YOR123C | -0.6594 | 0.09549 | -6.905 | 5.069e-12 | 2.725e-10 | LEO1 | Phenotypic enhancement | yku70 | | YOL081W | -0.6586 | 0.09549 | -6.897 | 5.361e-12 | 2.846e-10 | IRA2 | Phenotypic enhancement | yku70 | | YKR035W-A | -0.6524 | 0.09549 | -6.832 | 8.458e-12 | 4.325e-10 | DID2 | Phenotypic enhancement | yku70 | | YBL025W | -0.6456 | 0.09549 | -6.761 | 1.386e-11 | 7.002e-10 | RRN10 | Phenotypic enhancement | yku70 | | YDR024W | -0.6450 | 0.09549 | -6.755 | 1.439e-11 | 7.185e-10 | FYV1 | Phenotypic enhancement | yku70 | | YOL061W | -0.6425 | 0.09549 | -6.728 | 1.735e-11 | 8.557e-10 | PRS5 | Phenotypic enhancement | yku70 | | YDR372C | -0.6342 | 0.09549 | -6.641 | 3.131e-11 | 1.509e-09 | VPS74 | Phenotypic enhancement | yku70 | | YMR274C | -0.6287 | 0.09549 | -6.584 | 4.606e-11 | 2.194e-09 | RCE1 | Phenotypic enhancement | yku70 | | YDR369C | -0.6268 | 0.09549 | -6.564 | 5.270e-11 | 2.483e-09 | XRS2 | Phenotypic enhancement | yku70 | | YJR083C | -0.6231 | 0.09549 | -6.526 | 6.818e-11 | 3.142e-09 | ACF4 | Phenotypic enhancement | yku70 | | YER095W | -0.6224 | 0.09549 | -6.518 | 7.167e-11 | 3.266e-09 | RAD51 | Phenotypic enhancement | yku70 | | YIL040W | -0.6219 | 0.09549 | -6.513 | 7.438e-11 | 3.353e-09 | APQ12 | Phenotypic enhancement | yku70 | | YDR388W | -0.6201 | 0.09549 | -6.494 | 8.412e-11 | 3.752e-09 | RVS167 | Phenotypic enhancement | yku70 | | YDL190C | -0.6170 | 0.09549 | -6.461 | 1.045e-10 | 4.477e-09 | UFD2 | Phenotypic enhancement | yku70 | | YJR075W | -0.6170 | 0.09549 | -6.461 | 1.046e-10 | 4.477e-09 | HOC1 | Phenotypic enhancement | yku70 | | YPL079W | -0.6162 | 0.09549 | -6.453 | 1.102e-10 | 4.668e-09 | RPL21B | Phenotypic enhancement | yku70 | | YDR074W | -0.6094 | 0.09549 | -6.381 | 1.767e-10 | 7.335e-09 | TPS2 | Phenotypic enhancement | yku70 | | YNL307C | -0.6061 | 0.09549 | -6.347 | 2.206e-10 | 8.896e-09 | MCK1 | Phenotypic enhancement | yku70 | | YJL062W | -0.6050 | 0.09549 | -6.336 | 2.378e-10 | 9.497e-09 | LAS21 | Phenotypic enhancement | yku70 | | YML013C-A | -0.6046 | 0.09549 | -6.332 | 2.439e-10 | 9.649e-09 |  | Phenotypic enhancement | yku70 | | YNL069C | -0.6030 | 0.09549 | -6.315 | 2.715e-10 | 1.064e-08 | RPL16B | Phenotypic enhancement | yku70 | | YNL097C | -0.5962 | 0.09549 | -6.244 | 4.292e-10 | 1.666e-08 | PHO23 | Phenotypic enhancement | yku70 | | YIR023W | -0.5947 | 0.09549 | -6.228 | 4.743e-10 | 1.825e-08 | DAL81 | Phenotypic enhancement | yku70 | | YBR194W | -0.5935 | 0.09549 | -6.215 | 5.170e-10 | 1.971e-08 | AIM4 | Phenotypic enhancement | yku70 | | YOL004W | -0.5907 | 0.09549 | -6.186 | 6.221e-10 | 2.350e-08 | SIN3 | Phenotypic enhancement | yku70 | | YMR179W | -0.5899 | 0.09549 | -6.178 | 6.533e-10 | 2.424e-08 | SPT21 | Phenotypic enhancement | yku70 | | YGL244W | -0.5874 | 0.09549 | -6.151 | 7.728e-10 | 2.843e-08 | RTF1 | Phenotypic enhancement | yku70 | | YMR166C | -0.5866 | 0.09549 | -6.143 | 8.153e-10 | 2.973e-08 | \_ | Phenotypic enhancement | yku70 | | YOR132W | -0.5706 | 0.09549 | -5.975 | 2.310e-09 | 8.210e-08 | VPS17 | Phenotypic enhancement | yku70 | | YMR039C | -0.5659 | 0.09549 | -5.926 | 3.112e-09 | 1.096e-07 | SUB1 | Phenotypic enhancement | yku70 | | YOR106W | -0.5649 | 0.09549 | -5.916 | 3.316e-09 | 1.159e-07 | VAM3 | Phenotypic enhancement | yku70 | | YIL084C | -0.5640 | 0.09549 | -5.907 | 3.509e-09 | 1.216e-07 | SDS3 | Phenotypic enhancement | yku70 | | YML032C | -0.5588 | 0.03609 | -15.480 | 0.000e+00 | 0.000e+00 | RAD52 | Phenotypic enhancement | yku70 | | YNL206C | -0.5532 | 0.09549 | -5.793 | 6.943e-09 | 2.367e-07 | RTT106 | Phenotypic enhancement | yku70 | | YDR207C | -0.5520 | 0.09549 | -5.780 | 7.487e-09 | 2.532e-07 | UME6 | Phenotypic enhancement | yku70 | | YKL212W | -0.5506 | 0.09549 | -5.766 | 8.150e-09 | 2.734e-07 | SAC1 | Phenotypic enhancement | yku70 | | YEL033W | -0.5495 | 0.09549 | -5.755 | 8.707e-09 | 2.897e-07 | MTC7 | Phenotypic enhancement | yku70 | | YMR263W | -0.5473 | 0.09549 | -5.731 | 9.999e-09 | 3.301e-07 | SAP30 | Phenotypic enhancement | yku70 | | YAL058C-A | -0.5441 | 0.09549 | -5.698 | 1.218e-08 | 3.990e-07 |  | Phenotypic enhancement | yku70 | | YMR273C | -0.5415 | 0.09549 | -5.670 | 1.431e-08 | 4.651e-07 | ZDS1 | Phenotypic enhancement | yku70 | | YML107C | -0.5393 | 0.09549 | -5.648 | 1.632e-08 | 5.184e-07 | PML39 | Phenotypic enhancement | yku70 | | YDR162C | -0.5345 | 0.09549 | -5.597 | 2.188e-08 | 6.898e-07 | NBP2 | Phenotypic enhancement | yku70 | | YLR402W | -0.5343 | 0.09549 | -5.595 | 2.215e-08 | 6.931e-07 | \_ | Phenotypic enhancement | yku70 | | YNL106C | -0.5313 | 0.09549 | -5.564 | 2.650e-08 | 8.230e-07 | INP52 | Phenotypic enhancement | yku70 | | YML097C | -0.5295 | 0.09549 | -5.545 | 2.948e-08 | 9.040e-07 | VPS9 | Phenotypic enhancement | yku70 | | YPL144W | -0.5295 | 0.09549 | -5.545 | 2.954e-08 | 9.040e-07 | POC4 | Phenotypic enhancement | yku70 | | YOL086C | -0.5288 | 0.09549 | -5.538 | 3.075e-08 | 9.345e-07 | ADH1 | Phenotypic enhancement | yku70 | | YGL168W | -0.5218 | 0.09549 | -5.464 | 4.670e-08 | 1.399e-06 | HUR1 | Phenotypic enhancement | yku70 | | YPL087W | -0.5209 | 0.09549 | -5.455 | 4.921e-08 | 1.463e-06 | YDC1 | Phenotypic enhancement | yku70 | | YPL055C | -0.5198 | 0.09549 | -5.444 | 5.234e-08 | 1.545e-06 | LGE1 | Phenotypic enhancement | yku70 | | YLR386W | -0.5188 | 0.09549 | -5.433 | 5.549e-08 | 1.627e-06 | VAC14 | Phenotypic enhancement | yku70 | | YMR272C | -0.5157 | 0.09549 | -5.401 | 6.658e-08 | 1.939e-06 | SCS7 | Phenotypic enhancement | yku70 | | YML117W-A | -0.5107 | 0.09549 | -5.348 | 8.905e-08 | 2.575e-06 |  | Phenotypic enhancement | yku70 | | YDR076W | -0.5100 | 0.09549 | -5.341 | 9.292e-08 | 2.669e-06 | RAD55 | Phenotypic enhancement | yku70 | | YPL004C | -0.5086 | 0.09549 | -5.326 | 1.007e-07 | 2.853e-06 | LSP1 | Phenotypic enhancement | yku70 | | YDR469W | -0.5061 | 0.09549 | -5.300 | 1.160e-07 | 3.242e-06 | SDC1 | Phenotypic enhancement | yku70 | | YPL080C | -0.5044 | 0.09549 | -5.282 | 1.281e-07 | 3.558e-06 | \_ | Phenotypic enhancement | yku70 | | YIL098C | 0.5401 | 0.09549 | 5.656 | 1.556e-08 | 5.019e-07 | FMC1 | Phenotypic suppression | yku70 | | YGL255W | 0.5745 | 0.09549 | 6.017 | 1.792e-09 | 6.421e-08 | ZRT1 | Phenotypic suppression | yku70 | | YLL006W | 0.6192 | 0.09549 | 6.485 | 8.963e-11 | 3.956e-09 | MMM1 | Phenotypic suppression | yku70 | | YPL069C | 0.6392 | 0.09549 | 6.694 | 2.186e-11 | 1.066e-09 | BTS1 | Phenotypic suppression | yku70 | | YML038C | 0.8556 | 0.09549 | 8.960 | 0.000e+00 | 0.000e+00 | YMD8 | Phenotypic suppression | yku70 | |
