# Supplementary material for: Quantitative Fitness Analysis Shows That NMD Proteins and Many Other Protein Complexes Suppress or Enhance Distinct Telomere Cap Defects
Source: PLoS Genet. 2011 Apr 7;7(4):e1001362. doi: 10.1371/journal.pgen.1001362 (PMC3072368; doi:10.1371/journal.pgen.1001362)
Supplement: Table S4 — List of suppressors and enhancers of yku70Δ defect at 37.5°C. A list of genes which, when deleted, result in suppression or enhancement of the yku70Δ phenotype at 37.5°C. Only included are gene deletions which passed a 5% FDR cutoff and had a GIS of greater than 0.5 (+ or −) in magnitude. http://research.ncl.ac.uk/colonyzer/AddinallQFA/S4_yku70_375.html. See http://research.ncl.ac.uk/colonyzer/AddinallQFA for a list of all significant interactors, a GIS plot showing interactors and raw data. (0.10 MB HTML) [file pgen.1001362.s008.html]

Genetic interaction hitlist after QFA

yku70Δ at 37.5° C

| | ORF | GIS | stderr | tval | pval | qval | genename | interaction | query | | --- | --- | --- | --- | --- | --- | --- | --- | --- | | YBL088C | -1.0590 | 0.10240 | -10.340 | 0.000e+00 | 0.000e+00 | TEL1 | Phenotypic enhancement | yku70 | | YLR014C | -1.0530 | 0.10240 | -10.290 | 0.000e+00 | 0.000e+00 | PPR1 | Phenotypic enhancement | yku70 | | YNR024W | -1.0390 | 0.10240 | -10.150 | 0.000e+00 | 0.000e+00 | MPP6 | Phenotypic enhancement | yku70 | | YIL009C-A | -1.0290 | 0.10240 | -10.050 | 0.000e+00 | 0.000e+00 | EST3 | Phenotypic enhancement | yku70 | | YPR046W | -1.0250 | 0.10240 | -10.020 | 0.000e+00 | 0.000e+00 | MCM16 | Phenotypic enhancement | yku70 | | YCR095C | -1.0010 | 0.10240 | -9.776 | 0.000e+00 | 0.000e+00 | OCA4 | Phenotypic enhancement | yku70 | | YDR067C | -0.9940 | 0.10240 | -9.711 | 0.000e+00 | 0.000e+00 | OCA6 | Phenotypic enhancement | yku70 | | YMR080C | -0.9905 | 0.10240 | -9.677 | 0.000e+00 | 0.000e+00 | UPF1 | Phenotypic enhancement | yku70 | | YNR025C | -0.9841 | 0.10240 | -9.614 | 0.000e+00 | 0.000e+00 | \_ | Phenotypic enhancement | yku70 | | YPR119W | -0.9831 | 0.03869 | -25.410 | 0.000e+00 | 0.000e+00 | CLB2 | Phenotypic enhancement | yku70 | | YJR084W | -0.9772 | 0.10240 | -9.547 | 0.000e+00 | 0.000e+00 | CSN12 | Phenotypic enhancement | yku70 | | YGR072W | -0.9594 | 0.10240 | -9.373 | 0.000e+00 | 0.000e+00 | UPF3 | Phenotypic enhancement | yku70 | | YGL016W | -0.9531 | 0.10240 | -9.312 | 0.000e+00 | 0.000e+00 | KAP122 | Phenotypic enhancement | yku70 | | YHR077C | -0.9489 | 0.03869 | -24.530 | 0.000e+00 | 0.000e+00 | UPF2 | Phenotypic enhancement | yku70 | | YBL047C | -0.9338 | 0.10240 | -9.123 | 0.000e+00 | 0.000e+00 | EDE1 | Phenotypic enhancement | yku70 | | YJR075W | -0.9209 | 0.10240 | -8.997 | 0.000e+00 | 0.000e+00 | HOC1 | Phenotypic enhancement | yku70 | | YPL139C | -0.9184 | 0.10240 | -8.972 | 0.000e+00 | 0.000e+00 | UME1 | Phenotypic enhancement | yku70 | | YDR024W | -0.9175 | 0.10240 | -8.963 | 0.000e+00 | 0.000e+00 | FYV1 | Phenotypic enhancement | yku70 | | YDR025W | -0.9168 | 0.10240 | -8.957 | 0.000e+00 | 0.000e+00 | RPS11A | Phenotypic enhancement | yku70 | | YOL061W | -0.9020 | 0.10240 | -8.812 | 0.000e+00 | 0.000e+00 | PRS5 | Phenotypic enhancement | yku70 | | YBR034C | -0.8980 | 0.10240 | -8.773 | 0.000e+00 | 0.000e+00 | HMT1 | Phenotypic enhancement | yku70 | | YAL056W | -0.8971 | 0.10240 | -8.764 | 0.000e+00 | 0.000e+00 | GPB2 | Phenotypic enhancement | yku70 | | YMR216C | -0.8943 | 0.10240 | -8.737 | 0.000e+00 | 0.000e+00 | SKY1 | Phenotypic enhancement | yku70 | | YDR289C | -0.8799 | 0.10240 | -8.596 | 0.000e+00 | 0.000e+00 | RTT103 | Phenotypic enhancement | yku70 | | YDR372C | -0.8749 | 0.10240 | -8.547 | 0.000e+00 | 0.000e+00 | VPS74 | Phenotypic enhancement | yku70 | | YCR034W | -0.8745 | 0.10240 | -8.544 | 0.000e+00 | 0.000e+00 | FEN1 | Phenotypic enhancement | yku70 | | YGL066W | -0.8745 | 0.10240 | -8.544 | 0.000e+00 | 0.000e+00 | SGF73 | Phenotypic enhancement | yku70 | | YNL032W | -0.8725 | 0.10240 | -8.524 | 0.000e+00 | 0.000e+00 | SIW14 | Phenotypic enhancement | yku70 | | YLR055C | -0.8707 | 0.10240 | -8.506 | 0.000e+00 | 0.000e+00 | SPT8 | Phenotypic enhancement | yku70 | | YKR035C | -0.8687 | 0.10240 | -8.487 | 0.000e+00 | 0.000e+00 | OPI8 | Phenotypic enhancement | yku70 | | YNL099C | -0.8537 | 0.10240 | -8.340 | 0.000e+00 | 0.000e+00 | OCA1 | Phenotypic enhancement | yku70 | | YPL100W | -0.8464 | 0.10240 | -8.269 | 2.220e-16 | 1.859e-14 | ATG21 | Phenotypic enhancement | yku70 | | YMR039C | -0.8345 | 0.10240 | -8.152 | 4.441e-16 | 3.575e-14 | SUB1 | Phenotypic enhancement | yku70 | | YBR103W | -0.8271 | 0.10240 | -8.080 | 6.661e-16 | 5.261e-14 | SIF2 | Phenotypic enhancement | yku70 | | YGL163C | -0.8223 | 0.10240 | -8.034 | 8.882e-16 | 6.760e-14 | RAD54 | Phenotypic enhancement | yku70 | | YER161C | -0.8208 | 0.10240 | -8.019 | 1.110e-15 | 8.299e-14 | SPT2 | Phenotypic enhancement | yku70 | | YNL056W | -0.8165 | 0.10240 | -7.977 | 1.554e-15 | 1.141e-13 | OCA2 | Phenotypic enhancement | yku70 | | YPR060C | -0.8144 | 0.10240 | -7.956 | 1.776e-15 | 1.260e-13 | ARO7 | Phenotypic enhancement | yku70 | | YHL029C | -0.8129 | 0.10240 | -7.942 | 1.998e-15 | 1.371e-13 | OCA5 | Phenotypic enhancement | yku70 | | YOR054C | -0.8116 | 0.10240 | -7.929 | 2.220e-15 | 1.499e-13 | VHS3 | Phenotypic enhancement | yku70 | | YAL058C-A | -0.8105 | 0.10240 | -7.919 | 2.442e-15 | 1.623e-13 |  | Phenotypic enhancement | yku70 | | YBR187W | -0.8060 | 0.10240 | -7.874 | 3.553e-15 | 2.288e-13 | GDT1 | Phenotypic enhancement | yku70 | | YBR090C-A | -0.8031 | 0.10240 | -7.846 | 4.441e-15 | 2.734e-13 |  | Phenotypic enhancement | yku70 | | YDR431W | -0.8031 | 0.10240 | -7.846 | 4.441e-15 | 2.734e-13 | \_ | Phenotypic enhancement | yku70 | | YDR486C | -0.8030 | 0.10240 | -7.845 | 4.441e-15 | 2.734e-13 | VPS60 | Phenotypic enhancement | yku70 | | YNL307C | -0.8022 | 0.10240 | -7.837 | 4.663e-15 | 2.829e-13 | MCK1 | Phenotypic enhancement | yku70 | | YOR069W | -0.7962 | 0.10240 | -7.778 | 7.550e-15 | 4.515e-13 | VPS5 | Phenotypic enhancement | yku70 | | YDR090C | -0.7911 | 0.10240 | -7.728 | 1.110e-14 | 6.546e-13 | \_ | Phenotypic enhancement | yku70 | | YPL138C | -0.7895 | 0.10240 | -7.713 | 1.243e-14 | 7.229e-13 | SPP1 | Phenotypic enhancement | yku70 | | YML013C-A | -0.7893 | 0.10240 | -7.711 | 1.266e-14 | 7.258e-13 |  | Phenotypic enhancement | yku70 | | YNL068C | -0.7850 | 0.10240 | -7.669 | 1.754e-14 | 9.662e-13 | FKH2 | Phenotypic enhancement | yku70 | | YNL097C | -0.7839 | 0.10240 | -7.659 | 1.910e-14 | 1.038e-12 | PHO23 | Phenotypic enhancement | yku70 | | YAL011W | -0.7829 | 0.10240 | -7.649 | 2.043e-14 | 1.096e-12 | SWC3 | Phenotypic enhancement | yku70 | | YDR092W | -0.7810 | 0.10240 | -7.630 | 2.376e-14 | 1.255e-12 | UBC13 | Phenotypic enhancement | yku70 | | YLR085C | -0.7809 | 0.10240 | -7.629 | 2.398e-14 | 1.255e-12 | ARP6 | Phenotypic enhancement | yku70 | | YJL062W | -0.7772 | 0.10240 | -7.593 | 3.175e-14 | 1.621e-12 | LAS21 | Phenotypic enhancement | yku70 | | YMR179W | -0.7681 | 0.10240 | -7.504 | 6.262e-14 | 3.158e-12 | SPT21 | Phenotypic enhancement | yku70 | | YBR272C | -0.7629 | 0.10240 | -7.454 | 9.193e-14 | 4.581e-12 | HSM3 | Phenotypic enhancement | yku70 | | YLR233C | -0.7615 | 0.03869 | -19.680 | 0.000e+00 | 0.000e+00 | EST1 | Phenotypic enhancement | yku70 | | YPL057C | -0.7612 | 0.10240 | -7.437 | 1.046e-13 | 5.150e-12 | SUR1 | Phenotypic enhancement | yku70 | | YAL023C | -0.7587 | 0.10240 | -7.412 | 1.259e-13 | 6.128e-12 | PMT2 | Phenotypic enhancement | yku70 | | YDR004W | -0.7558 | 0.10240 | -7.383 | 1.561e-13 | 7.425e-12 | RAD57 | Phenotypic enhancement | yku70 | | YLR015W | -0.7498 | 0.10240 | -7.325 | 2.414e-13 | 1.135e-11 | BRE2 | Phenotypic enhancement | yku70 | | YBR229C | -0.7472 | 0.10240 | -7.299 | 2.922e-13 | 1.345e-11 | ROT2 | Phenotypic enhancement | yku70 | | YBL089W | -0.7471 | 0.10240 | -7.299 | 2.924e-13 | 1.345e-11 | AVT5 | Phenotypic enhancement | yku70 | | YCR071C | -0.7465 | 0.10240 | -7.293 | 3.064e-13 | 1.394e-11 | IMG2 | Phenotypic enhancement | yku70 | | YLR021W | -0.7420 | 0.10240 | -7.249 | 4.254e-13 | 1.882e-11 | IRC25 | Phenotypic enhancement | yku70 | | YBR231C | -0.7412 | 0.10240 | -7.242 | 4.481e-13 | 1.954e-11 | SWC5 | Phenotypic enhancement | yku70 | | YDR120C | -0.7395 | 0.10240 | -7.225 | 5.078e-13 | 2.191e-11 | TRM1 | Phenotypic enhancement | yku70 | | YPL090C | -0.7369 | 0.10240 | -7.199 | 6.142e-13 | 2.623e-11 | RPS6A | Phenotypic enhancement | yku70 | | YJR118C | -0.7338 | 0.10240 | -7.169 | 7.629e-13 | 3.226e-11 | ILM1 | Phenotypic enhancement | yku70 | | YNL286W | -0.7271 | 0.10240 | -7.104 | 1.228e-12 | 5.141e-11 | CUS2 | Phenotypic enhancement | yku70 | | YPL080C | -0.7261 | 0.10240 | -7.094 | 1.317e-12 | 5.457e-11 | \_ | Phenotypic enhancement | yku70 | | YAL020C | -0.7240 | 0.10240 | -7.073 | 1.527e-12 | 6.266e-11 | ATS1 | Phenotypic enhancement | yku70 | | YBR188C | -0.7234 | 0.10240 | -7.068 | 1.593e-12 | 6.472e-11 | NTC20 | Phenotypic enhancement | yku70 | | YDL190C | -0.7230 | 0.10240 | -7.063 | 1.640e-12 | 6.600e-11 | UFD2 | Phenotypic enhancement | yku70 | | YDR392W | -0.7191 | 0.10240 | -7.026 | 2.152e-12 | 8.499e-11 | SPT3 | Phenotypic enhancement | yku70 | | YDR076W | -0.7181 | 0.10240 | -7.015 | 2.320e-12 | 9.077e-11 | RAD55 | Phenotypic enhancement | yku70 | | YDL170W | -0.7176 | 0.10240 | -7.010 | 2.401e-12 | 9.305e-11 | UGA3 | Phenotypic enhancement | yku70 | | YOR027W | -0.7165 | 0.10240 | -7.000 | 2.587e-12 | 9.937e-11 | STI1 | Phenotypic enhancement | yku70 | | YBL025W | -0.7116 | 0.10240 | -6.952 | 3.644e-12 | 1.374e-10 | RRN10 | Phenotypic enhancement | yku70 | | YPL079W | -0.7111 | 0.10240 | -6.947 | 3.753e-12 | 1.403e-10 | RPL21B | Phenotypic enhancement | yku70 | | YER095W | -0.7110 | 0.10240 | -6.946 | 3.791e-12 | 1.404e-10 | RAD51 | Phenotypic enhancement | yku70 | | YLR181C | -0.7104 | 0.10240 | -6.940 | 3.953e-12 | 1.451e-10 | VTA1 | Phenotypic enhancement | yku70 | | YMR263W | -0.7077 | 0.10240 | -6.914 | 4.769e-12 | 1.736e-10 | SAP30 | Phenotypic enhancement | yku70 | | YML041C | -0.7074 | 0.10240 | -6.911 | 4.859e-12 | 1.754e-10 | VPS71 | Phenotypic enhancement | yku70 | | YKL110C | -0.7050 | 0.10240 | -6.887 | 5.733e-12 | 2.034e-10 | KTI12 | Phenotypic enhancement | yku70 | | YKL032C | -0.6966 | 0.10240 | -6.805 | 1.018e-11 | 3.581e-10 | IXR1 | Phenotypic enhancement | yku70 | | YFR040W | -0.6948 | 0.10240 | -6.788 | 1.148e-11 | 4.004e-10 | SAP155 | Phenotypic enhancement | yku70 | | YAL026C | -0.6933 | 0.10240 | -6.773 | 1.272e-11 | 4.400e-10 | DRS2 | Phenotypic enhancement | yku70 | | YDR389W | -0.6918 | 0.10240 | -6.759 | 1.404e-11 | 4.818e-10 | SAC7 | Phenotypic enhancement | yku70 | | YOR070C | -0.6908 | 0.10240 | -6.749 | 1.504e-11 | 5.119e-10 | GYP1 | Phenotypic enhancement | yku70 | | YCR033W | -0.6902 | 0.10240 | -6.743 | 1.569e-11 | 5.298e-10 | SNT1 | Phenotypic enhancement | yku70 | | YBR078W | -0.6895 | 0.10240 | -6.736 | 1.642e-11 | 5.500e-10 | ECM33 | Phenotypic enhancement | yku70 | | YDR388W | -0.6886 | 0.10240 | -6.727 | 1.744e-11 | 5.794e-10 | RVS167 | Phenotypic enhancement | yku70 | | YKR010C | -0.6823 | 0.10240 | -6.666 | 2.646e-11 | 8.721e-10 | TOF2 | Phenotypic enhancement | yku70 | | YKL037W | -0.6803 | 0.10240 | -6.646 | 3.035e-11 | 9.925e-10 | AIM26 | Phenotypic enhancement | yku70 | | YBL049W | -0.6788 | 0.10240 | -6.632 | 3.347e-11 | 1.086e-09 | MOH1 | Phenotypic enhancement | yku70 | | YLR402W | -0.6784 | 0.10240 | -6.628 | 3.441e-11 | 1.108e-09 | \_ | Phenotypic enhancement | yku70 | | YOR132W | -0.6756 | 0.10240 | -6.600 | 4.136e-11 | 1.321e-09 | VPS17 | Phenotypic enhancement | yku70 | | YGR229C | -0.6754 | 0.10240 | -6.598 | 4.198e-11 | 1.331e-09 | SMI1 | Phenotypic enhancement | yku70 | | YNL069C | -0.6694 | 0.10240 | -6.540 | 6.188e-11 | 1.933e-09 | RPL16B | Phenotypic enhancement | yku70 | | YBL104C | -0.6689 | 0.10240 | -6.535 | 6.428e-11 | 1.993e-09 | \_ | Phenotypic enhancement | yku70 | | YCL060C | -0.6684 | 0.10240 | -6.530 | 6.612e-11 | 2.035e-09 |  | Phenotypic enhancement | yku70 | | YIR023W | -0.6678 | 0.10240 | -6.525 | 6.872e-11 | 2.100e-09 | DAL81 | Phenotypic enhancement | yku70 | | YBR194W | -0.6662 | 0.10240 | -6.509 | 7.636e-11 | 2.300e-09 | AIM4 | Phenotypic enhancement | yku70 | | YDL077C | -0.6627 | 0.10240 | -6.475 | 9.572e-11 | 2.862e-09 | VAM6 | Phenotypic enhancement | yku70 | | YBR036C | -0.6590 | 0.10240 | -6.438 | 1.215e-10 | 3.608e-09 | CSG2 | Phenotypic enhancement | yku70 | | YOR123C | -0.6585 | 0.10240 | -6.433 | 1.256e-10 | 3.702e-09 | LEO1 | Phenotypic enhancement | yku70 | | YPL120W | -0.6582 | 0.10240 | -6.430 | 1.285e-10 | 3.760e-09 | VPS30 | Phenotypic enhancement | yku70 | | YOL081W | -0.6578 | 0.10240 | -6.426 | 1.319e-10 | 3.833e-09 | IRA2 | Phenotypic enhancement | yku70 | | YOL086C | -0.6573 | 0.10240 | -6.421 | 1.359e-10 | 3.923e-09 | ADH1 | Phenotypic enhancement | yku70 | | YDR310C | -0.6549 | 0.10240 | -6.398 | 1.580e-10 | 4.469e-09 | SUM1 | Phenotypic enhancement | yku70 | | YKR072C | -0.6533 | 0.10240 | -6.383 | 1.751e-10 | 4.920e-09 | SIS2 | Phenotypic enhancement | yku70 | | YGR282C | -0.6527 | 0.10240 | -6.377 | 1.821e-10 | 5.082e-09 | BGL2 | Phenotypic enhancement | yku70 | | YLR429W | -0.6520 | 0.10240 | -6.370 | 1.905e-10 | 5.282e-09 | CRN1 | Phenotypic enhancement | yku70 | | YKR035W-A | -0.6515 | 0.10240 | -6.365 | 1.961e-10 | 5.401e-09 | DID2 | Phenotypic enhancement | yku70 | | YLR144C | -0.6456 | 0.10240 | -6.307 | 2.859e-10 | 7.821e-09 | ACF2 | Phenotypic enhancement | yku70 | | YDR477W | -0.6436 | 0.10240 | -6.288 | 3.239e-10 | 8.805e-09 | SNF1 | Phenotypic enhancement | yku70 | | YDR075W | -0.6393 | 0.10240 | -6.246 | 4.250e-10 | 1.140e-08 | PPH3 | Phenotypic enhancement | yku70 | | YDR334W | -0.6386 | 0.10240 | -6.239 | 4.439e-10 | 1.184e-08 | SWR1 | Phenotypic enhancement | yku70 | | YMR312W | -0.6364 | 0.10240 | -6.218 | 5.081e-10 | 1.346e-08 | ELP6 | Phenotypic enhancement | yku70 | | YPR006C | -0.6352 | 0.10240 | -6.206 | 5.480e-10 | 1.443e-08 | ICL2 | Phenotypic enhancement | yku70 | | YIL153W | -0.6348 | 0.10240 | -6.202 | 5.610e-10 | 1.468e-08 | RRD1 | Phenotypic enhancement | yku70 | | YLR386W | -0.6342 | 0.10240 | -6.196 | 5.836e-10 | 1.517e-08 | VAC14 | Phenotypic enhancement | yku70 | | YLR449W | -0.6326 | 0.10240 | -6.180 | 6.429e-10 | 1.661e-08 | FPR4 | Phenotypic enhancement | yku70 | | YLR360W | -0.6296 | 0.10240 | -6.151 | 7.737e-10 | 1.983e-08 | VPS38 | Phenotypic enhancement | yku70 | | YGL020C | -0.6296 | 0.10240 | -6.150 | 7.769e-10 | 1.983e-08 | GET1 | Phenotypic enhancement | yku70 | | YAL004W | -0.6286 | 0.10240 | -6.142 | 8.214e-10 | 2.084e-08 | \_ | Phenotypic enhancement | yku70 | | YML097C | -0.6283 | 0.10240 | -6.139 | 8.376e-10 | 2.112e-08 | VPS9 | Phenotypic enhancement | yku70 | | YLR361C | -0.6274 | 0.10240 | -6.129 | 8.894e-10 | 2.229e-08 | DCR2 | Phenotypic enhancement | yku70 | | YDR369C | -0.6260 | 0.10240 | -6.116 | 9.647e-10 | 2.403e-08 | XRS2 | Phenotypic enhancement | yku70 | | YGL042C | -0.6250 | 0.10240 | -6.106 | 1.030e-09 | 2.515e-08 | \_ | Phenotypic enhancement | yku70 | | YDR290W | -0.6249 | 0.10240 | -6.105 | 1.034e-09 | 2.515e-08 | \_ | Phenotypic enhancement | yku70 | | YJR083C | -0.6243 | 0.10240 | -6.099 | 1.072e-09 | 2.594e-08 | ACF4 | Phenotypic enhancement | yku70 | | YNR005C | -0.6234 | 0.10240 | -6.090 | 1.135e-09 | 2.729e-08 | \_ | Phenotypic enhancement | yku70 | | YGL124C | -0.6218 | 0.10240 | -6.075 | 1.248e-09 | 2.969e-08 | MON1 | Phenotypic enhancement | yku70 | | YIL040W | -0.6211 | 0.10240 | -6.068 | 1.302e-09 | 3.080e-08 | APQ12 | Phenotypic enhancement | yku70 | | YEL033W | -0.6209 | 0.10240 | -6.066 | 1.322e-09 | 3.109e-08 | MTC7 | Phenotypic enhancement | yku70 | | YCR032W | -0.6175 | 0.10240 | -6.032 | 1.624e-09 | 3.776e-08 | BPH1 | Phenotypic enhancement | yku70 | | YPL101W | -0.6147 | 0.10240 | -6.006 | 1.916e-09 | 4.382e-08 | ELP4 | Phenotypic enhancement | yku70 | | YKL213C | -0.6111 | 0.10240 | -5.970 | 2.385e-09 | 5.397e-08 | DOA1 | Phenotypic enhancement | yku70 | | YDR074W | -0.6086 | 0.10240 | -5.946 | 2.767e-09 | 6.227e-08 | TPS2 | Phenotypic enhancement | yku70 | | YBR207W | -0.6079 | 0.10240 | -5.939 | 2.875e-09 | 6.436e-08 | FTH1 | Phenotypic enhancement | yku70 | | YPR057W | -0.6031 | 0.10240 | -5.892 | 3.843e-09 | 8.558e-08 | BRR1 | Phenotypic enhancement | yku70 | | YMR166C | -0.5944 | 0.10240 | -5.807 | 6.396e-09 | 1.409e-07 | \_ | Phenotypic enhancement | yku70 | | YPL144W | -0.5941 | 0.10240 | -5.804 | 6.512e-09 | 1.427e-07 | POC4 | Phenotypic enhancement | yku70 | | YMR274C | -0.5915 | 0.10240 | -5.779 | 7.557e-09 | 1.648e-07 | RCE1 | Phenotypic enhancement | yku70 | | YOL004W | -0.5899 | 0.10240 | -5.763 | 8.284e-09 | 1.797e-07 | SIN3 | Phenotypic enhancement | yku70 | | YBL051C | -0.5869 | 0.10240 | -5.734 | 9.846e-09 | 2.114e-07 | PIN4 | Phenotypic enhancement | yku70 | | YGL244W | -0.5867 | 0.10240 | -5.731 | 1.001e-08 | 2.137e-07 | RTF1 | Phenotypic enhancement | yku70 | | YOR291W | -0.5814 | 0.10240 | -5.680 | 1.355e-08 | 2.865e-07 | YPK9 | Phenotypic enhancement | yku70 | | YBL031W | -0.5778 | 0.10240 | -5.645 | 1.662e-08 | 3.495e-07 | SHE1 | Phenotypic enhancement | yku70 | | YHR200W | -0.5775 | 0.10240 | -5.642 | 1.688e-08 | 3.533e-07 | RPN10 | Phenotypic enhancement | yku70 | | YJL148W | -0.5761 | 0.10240 | -5.629 | 1.824e-08 | 3.799e-07 | RPA34 | Phenotypic enhancement | yku70 | | YFR010W | -0.5653 | 0.10240 | -5.522 | 3.357e-08 | 6.888e-07 | UBP6 | Phenotypic enhancement | yku70 | | YPL047W | -0.5648 | 0.10240 | -5.517 | 3.453e-08 | 7.051e-07 | SGF11 | Phenotypic enhancement | yku70 | | YOR106W | -0.5642 | 0.10240 | -5.512 | 3.561e-08 | 7.236e-07 | VAM3 | Phenotypic enhancement | yku70 | | YNL206C | -0.5628 | 0.10240 | -5.499 | 3.838e-08 | 7.761e-07 | RTT106 | Phenotypic enhancement | yku70 | | YAL005C | -0.5622 | 0.10240 | -5.492 | 3.986e-08 | 8.022e-07 | SSA1 | Phenotypic enhancement | yku70 | | YNL162W | -0.5619 | 0.10240 | -5.489 | 4.056e-08 | 8.123e-07 | RPL42A | Phenotypic enhancement | yku70 | | YBR057C | -0.5616 | 0.10240 | -5.487 | 4.109e-08 | 8.191e-07 | MUM2 | Phenotypic enhancement | yku70 | | YHR012W | -0.5596 | 0.10240 | -5.467 | 4.601e-08 | 9.085e-07 | VPS29 | Phenotypic enhancement | yku70 | | YML032C | -0.5581 | 0.03869 | -14.430 | 0.000e+00 | 0.000e+00 | RAD52 | Phenotypic enhancement | yku70 | | YOL050C | -0.5578 | 0.10240 | -5.449 | 5.078e-08 | 9.981e-07 | \_ | Phenotypic enhancement | yku70 | | YIL084C | -0.5562 | 0.10240 | -5.434 | 5.532e-08 | 1.082e-06 | SDS3 | Phenotypic enhancement | yku70 | | YOL116W | -0.5534 | 0.10240 | -5.407 | 6.443e-08 | 1.254e-06 | MSN1 | Phenotypic enhancement | yku70 | | YDR207C | -0.5513 | 0.10240 | -5.386 | 7.240e-08 | 1.403e-06 | UME6 | Phenotypic enhancement | yku70 | | YKL212W | -0.5499 | 0.10240 | -5.372 | 7.796e-08 | 1.504e-06 | SAC1 | Phenotypic enhancement | yku70 | | YPL102C | -0.5477 | 0.10240 | -5.350 | 8.805e-08 | 1.675e-06 | \_ | Phenotypic enhancement | yku70 | | YGR061C | -0.5437 | 0.10240 | -5.312 | 1.087e-07 | 2.059e-06 | ADE6 | Phenotypic enhancement | yku70 | | YML001W | -0.5391 | 0.10240 | -5.267 | 1.389e-07 | 2.616e-06 | YPT7 | Phenotypic enhancement | yku70 | | YHR031C | -0.5391 | 0.10240 | -5.267 | 1.393e-07 | 2.616e-06 | RRM3 | Phenotypic enhancement | yku70 | | YBR227C | -0.5387 | 0.10240 | -5.263 | 1.425e-07 | 2.663e-06 | MCX1 | Phenotypic enhancement | yku70 | | YPR125W | -0.5383 | 0.10240 | -5.259 | 1.454e-07 | 2.704e-06 | YLH47 | Phenotypic enhancement | yku70 | | YOR120W | -0.5380 | 0.10240 | -5.256 | 1.474e-07 | 2.730e-06 | GCY1 | Phenotypic enhancement | yku70 | | YBR134W | -0.5371 | 0.10240 | -5.247 | 1.550e-07 | 2.859e-06 | \_ | Phenotypic enhancement | yku70 | | YOR068C | -0.5361 | 0.10240 | -5.238 | 1.632e-07 | 2.996e-06 | VAM10 | Phenotypic enhancement | yku70 | | YDR162C | -0.5338 | 0.10240 | -5.215 | 1.844e-07 | 3.371e-06 | NBP2 | Phenotypic enhancement | yku70 | | YNL106C | -0.5306 | 0.10240 | -5.184 | 2.179e-07 | 3.966e-06 | INP52 | Phenotypic enhancement | yku70 | | YNL107W | -0.5295 | 0.10240 | -5.173 | 2.307e-07 | 4.181e-06 | YAF9 | Phenotypic enhancement | yku70 | | YBR059C | -0.5290 | 0.10240 | -5.168 | 2.376e-07 | 4.287e-06 | AKL1 | Phenotypic enhancement | yku70 | | YJR145C | -0.5282 | 0.10240 | -5.160 | 2.478e-07 | 4.434e-06 | RPS4A | Phenotypic enhancement | yku70 | | YBR114W | -0.5270 | 0.10240 | -5.148 | 2.634e-07 | 4.692e-06 | RAD16 | Phenotypic enhancement | yku70 | | YGR018C | -0.5247 | 0.10240 | -5.126 | 2.969e-07 | 5.266e-06 | \_ | Phenotypic enhancement | yku70 | | YAL008W | -0.5243 | 0.10240 | -5.122 | 3.032e-07 | 5.356e-06 | FUN14 | Phenotypic enhancement | yku70 | | YPL061W | -0.5229 | 0.10240 | -5.108 | 3.259e-07 | 5.732e-06 | ALD6 | Phenotypic enhancement | yku70 | | YGL168W | -0.5211 | 0.10240 | -5.091 | 3.571e-07 | 6.254e-06 | HUR1 | Phenotypic enhancement | yku70 | | YPL055C | -0.5192 | 0.10240 | -5.072 | 3.944e-07 | 6.823e-06 | LGE1 | Phenotypic enhancement | yku70 | | YML017W | -0.5189 | 0.10240 | -5.069 | 4.007e-07 | 6.875e-06 | PSP2 | Phenotypic enhancement | yku70 | | YEL031W | -0.5187 | 0.10240 | -5.068 | 4.039e-07 | 6.900e-06 | SPF1 | Phenotypic enhancement | yku70 | | YKR094C | -0.5178 | 0.10240 | -5.058 | 4.242e-07 | 7.218e-06 | RPL40B | Phenotypic enhancement | yku70 | | YHR033W | -0.5173 | 0.10240 | -5.053 | 4.352e-07 | 7.375e-06 | \_ | Phenotypic enhancement | yku70 | | YKL176C | -0.5168 | 0.10240 | -5.049 | 4.456e-07 | 7.521e-06 | LST4 | Phenotypic enhancement | yku70 | | YDR482C | -0.5153 | 0.10240 | -5.034 | 4.815e-07 | 8.094e-06 | CWC21 | Phenotypic enhancement | yku70 | | YGL115W | -0.5151 | 0.10240 | -5.032 | 4.866e-07 | 8.147e-06 | SNF4 | Phenotypic enhancement | yku70 | | YDL180W | -0.5138 | 0.10240 | -5.020 | 5.188e-07 | 8.651e-06 | \_ | Phenotypic enhancement | yku70 | | YAR040C | -0.5133 | 0.10240 | -5.014 | 5.337e-07 | 8.861e-06 |  | Phenotypic enhancement | yku70 | | YIL154C | -0.5132 | 0.10240 | -5.014 | 5.355e-07 | 8.861e-06 | IMP2' | Phenotypic enhancement | yku70 | | YER140W | -0.5123 | 0.10240 | -5.005 | 5.601e-07 | 9.231e-06 | \_ | Phenotypic enhancement | yku70 | | YDR043C | -0.5121 | 0.10240 | -5.003 | 5.649e-07 | 9.273e-06 | NRG1 | Phenotypic enhancement | yku70 | | YDR312W | -0.5113 | 0.10240 | -4.995 | 5.894e-07 | 9.637e-06 | SSF2 | Phenotypic enhancement | yku70 | | YBR025C | -0.5100 | 0.10240 | -4.982 | 6.307e-07 | 1.023e-05 | OLA1 | Phenotypic enhancement | yku70 | | YER151C | -0.5068 | 0.10240 | -4.951 | 7.395e-07 | 1.195e-05 | UBP3 | Phenotypic enhancement | yku70 | | YGL043W | -0.5059 | 0.10240 | -4.942 | 7.743e-07 | 1.247e-05 | DST1 | Phenotypic enhancement | yku70 | | YBR290W | -0.5057 | 0.10240 | -4.940 | 7.821e-07 | 1.250e-05 | BSD2 | Phenotypic enhancement | yku70 | | YDR469W | -0.5055 | 0.10240 | -4.938 | 7.894e-07 | 1.257e-05 | SDC1 | Phenotypic enhancement | yku70 | | YLR436C | -0.5052 | 0.10240 | -4.936 | 8.009e-07 | 1.270e-05 | ECM30 | Phenotypic enhancement | yku70 | | YMR272C | -0.5051 | 0.10240 | -4.935 | 8.049e-07 | 1.271e-05 | SCS7 | Phenotypic enhancement | yku70 | | YOR189W | -0.5037 | 0.10240 | -4.921 | 8.631e-07 | 1.357e-05 | IES4 | Phenotypic enhancement | yku70 | | YER111C | -0.5037 | 0.10240 | -4.920 | 8.654e-07 | 1.357e-05 | SWI4 | Phenotypic enhancement | yku70 | | YFL018C | -0.5023 | 0.10240 | -4.907 | 9.275e-07 | 1.443e-05 | LPD1 | Phenotypic enhancement | yku70 | | YLR335W | 0.5286 | 0.10240 | 5.164 | 2.427e-07 | 4.361e-06 | NUP2 | Phenotypic suppression | yku70 | | YLL006W | 0.6185 | 0.10240 | 6.042 | 1.526e-09 | 3.569e-08 | MMM1 | Phenotypic suppression | yku70 | | YPL069C | 0.6255 | 0.10240 | 6.111 | 9.960e-10 | 2.452e-08 | BTS1 | Phenotypic suppression | yku70 | | YJR074W | 0.6259 | 0.10240 | 6.115 | 9.702e-10 | 2.403e-08 | MOG1 | Phenotypic suppression | yku70 | | YML038C | 0.8235 | 0.10240 | 8.045 | 8.882e-16 | 6.760e-14 | YMD8 | Phenotypic suppression | yku70 | |
