# Supplementary material for: Quantitative Fitness Analysis Shows That NMD Proteins and Many Other Protein Complexes Suppress or Enhance Distinct Telomere Cap Defects
Source: PLoS Genet. 2011 Apr 7;7(4):e1001362. doi: 10.1371/journal.pgen.1001362 (PMC3072368; doi:10.1371/journal.pgen.1001362)
Supplement: Table S5 — List of suppressors and enhancers of cdc13-1 defect at 20°C. A list of genes which, when deleted, result in suppression or enhancement of the cdc13-1 phenotype at 20°C. Only included are gene deletions which passed a 5% FDR cutoff and had a GIS of greater than 0.5 (+ or −) in magnitude. http://research.ncl.ac.uk/colonyzer/AddinallQFA/S5_cdc131_20.html. See http://research.ncl.ac.uk/colonyzer/AddinallQFA for a list of all significant interactors, a GIS plot showing interactors and raw data. (0.01 MB HTML) [file pgen.1001362.s009.html]

Genetic interaction hitlist after QFA

cdc13-1 at 20° C

| | ORF | GIS | stderr | tval | pval | qval | genename | interaction | query | | --- | --- | --- | --- | --- | --- | --- | --- | --- | | YGL080W | -0.9401 | 0.05920 | -15.880 | 0 | 0 | FMP37 | Phenotypic enhancement | cdc13-1 | | YBL025W | -0.7705 | 0.05920 | -13.020 | 0 | 0 | RRN10 | Phenotypic enhancement | cdc13-1 | | YBR036C | -0.6221 | 0.05920 | -10.510 | 0 | 0 | CSG2 | Phenotypic enhancement | cdc13-1 | | YGR157W | -0.6094 | 0.06177 | -9.866 | 0 | 0 | CHO2 | Phenotypic enhancement | cdc13-1 | | YDL035C | -0.6072 | 0.05719 | -10.620 | 0 | 0 | GPR1 | Phenotypic enhancement | cdc13-1 | | YOL004W | -0.6068 | 0.05920 | -10.250 | 0 | 0 | SIN3 | Phenotypic enhancement | cdc13-1 | | YIL040W | -0.5311 | 0.05719 | -9.287 | 0 | 0 | APQ12 | Phenotypic enhancement | cdc13-1 | | YDR274C | 0.5552 | 0.05719 | 9.708 | 0 | 0 | \_ | Phenotypic suppression | cdc13-1 | | YDR435C | 0.5642 | 0.05719 | 9.865 | 0 | 0 | PPM1 | Phenotypic suppression | cdc13-1 | | YDR293C | 0.7436 | 0.05719 | 13.000 | 0 | 0 | SSD1 | Phenotypic suppression | cdc13-1 | |
