# Supplementary material for: Quantitative Fitness Analysis Shows That NMD Proteins and Many Other Protein Complexes Suppress or Enhance Distinct Telomere Cap Defects
Source: PLoS Genet. 2011 Apr 7;7(4):e1001362. doi: 10.1371/journal.pgen.1001362 (PMC3072368; doi:10.1371/journal.pgen.1001362)
Supplement: Table S6 — List of suppressors and enhancers of cdc13-1 defect at 27°C. A list of genes which, when deleted, result in suppression or enhancement of the cdc13-1 phenotype at 27°C. Only included are gene deletions which passed a 5% FDR cutoff and had a GIS of greater than 0.5 (+ or −) in magnitude. http://research.ncl.ac.uk/colonyzer/AddinallQFA/S6_cdc131_27.html. See http://research.ncl.ac.uk/colonyzer/AddinallQFA for a list of all significant interactors, a GIS plot showing interactors and raw data. (0.14 MB HTML) [file pgen.1001362.s010.html]

Genetic interaction hitlist after QFA

cdc13-1 at 27° C

| | ORF | GIS | stderr | tval | pval | qval | genename | interaction | query | | --- | --- | --- | --- | --- | --- | --- | --- | --- | | YMR106C | -1.0560 | 0.12950 | -8.148 | 4.441e-16 | 5.437e-14 | YKU80 | Phenotypic enhancement | cdc13-1 | | YKL191W | -1.0160 | 0.11990 | -8.467 | 0.000e+00 | 0.000e+00 | DPH2 | Phenotypic enhancement | cdc13-1 | | YDR260C | -1.0130 | 0.11990 | -8.449 | 0.000e+00 | 0.000e+00 | SWM1 | Phenotypic enhancement | cdc13-1 | | YGL014W | -1.0060 | 0.12410 | -8.106 | 4.441e-16 | 5.437e-14 | PUF4 | Phenotypic enhancement | cdc13-1 | | YBR275C | -0.9982 | 0.12410 | -8.041 | 8.882e-16 | 1.029e-13 | RIF1 | Phenotypic enhancement | cdc13-1 | | YCL060C | -0.9692 | 0.11990 | -8.081 | 6.661e-16 | 7.929e-14 |  | Phenotypic enhancement | cdc13-1 | | YCR020C-A | -0.9561 | 0.11990 | -7.972 | 1.554e-15 | 1.753e-13 | MAK31 | Phenotypic enhancement | cdc13-1 | | YCR079W | -0.9485 | 0.11990 | -7.908 | 2.665e-15 | 2.854e-13 | PTC6 | Phenotypic enhancement | cdc13-1 | | YBR072W | -0.9425 | 0.11990 | -7.858 | 3.997e-15 | 4.177e-13 | HSP26 | Phenotypic enhancement | cdc13-1 | | YOR027W | -0.9295 | 0.11990 | -7.750 | 9.326e-15 | 9.082e-13 | STI1 | Phenotypic enhancement | cdc13-1 | | YEL053C | -0.9221 | 0.11990 | -7.688 | 1.510e-14 | 1.407e-12 | MAK10 | Phenotypic enhancement | cdc13-1 | | YAL024C | -0.8962 | 0.11990 | -7.472 | 7.971e-14 | 6.831e-12 | LTE1 | Phenotypic enhancement | cdc13-1 | | YLR172C | -0.8910 | 0.11990 | -7.429 | 1.108e-13 | 9.130e-12 | DPH5 | Phenotypic enhancement | cdc13-1 | | YML062C | -0.8849 | 0.11990 | -7.378 | 1.630e-13 | 1.293e-11 | MFT1 | Phenotypic enhancement | cdc13-1 | | YBL088C | -0.8816 | 0.11990 | -7.351 | 1.992e-13 | 1.524e-11 | TEL1 | Phenotypic enhancement | cdc13-1 | | YIL103W | -0.8801 | 0.11990 | -7.338 | 2.189e-13 | 1.646e-11 | DPH1 | Phenotypic enhancement | cdc13-1 | | YMR073C | -0.8766 | 0.11990 | -7.309 | 2.731e-13 | 1.984e-11 | IRC21 | Phenotypic enhancement | cdc13-1 | | YPR120C | -0.8755 | 0.11990 | -7.300 | 2.913e-13 | 2.081e-11 | CLB5 | Phenotypic enhancement | cdc13-1 | | YGR123C | -0.8695 | 0.11990 | -7.250 | 4.217e-13 | 2.962e-11 | PPT1 | Phenotypic enhancement | cdc13-1 | | YJL092W | -0.8657 | 0.11990 | -7.218 | 5.338e-13 | 3.689e-11 | SRS2 | Phenotypic enhancement | cdc13-1 | | YCL061C | -0.8602 | 0.11990 | -7.172 | 7.443e-13 | 4.907e-11 | MRC1 | Phenotypic enhancement | cdc13-1 | | YOR322C | -0.8460 | 0.11990 | -7.054 | 1.759e-12 | 1.108e-10 | LDB19 | Phenotypic enhancement | cdc13-1 | | YHR167W | -0.8439 | 0.11990 | -7.036 | 1.995e-12 | 1.230e-10 | THP2 | Phenotypic enhancement | cdc13-1 | | YIL009C-A | -0.8415 | 0.11990 | -7.016 | 2.300e-12 | 1.388e-10 | EST3 | Phenotypic enhancement | cdc13-1 | | YJL124C | -0.8370 | 0.12950 | -6.461 | 1.049e-10 | 4.282e-09 | LSM1 | Phenotypic enhancement | cdc13-1 | | YER155C | -0.8254 | 0.12410 | -6.649 | 2.977e-11 | 1.433e-09 | BEM2 | Phenotypic enhancement | cdc13-1 | | YGR200C | -0.8218 | 0.11990 | -6.852 | 7.361e-12 | 3.993e-10 | ELP2 | Phenotypic enhancement | cdc13-1 | | YLL026W | -0.8204 | 0.11990 | -6.840 | 7.973e-12 | 4.270e-10 | HSP104 | Phenotypic enhancement | cdc13-1 | | YPL086C | -0.8029 | 0.11990 | -6.694 | 2.184e-11 | 1.101e-09 | ELP3 | Phenotypic enhancement | cdc13-1 | | YLR418C | -0.8011 | 0.11990 | -6.680 | 2.417e-11 | 1.204e-09 | CDC73 | Phenotypic enhancement | cdc13-1 | | YLR233C | -0.7960 | 0.04533 | -17.560 | 0.000e+00 | 0.000e+00 | EST1 | Phenotypic enhancement | cdc13-1 | | YDR075W | -0.7950 | 0.11990 | -6.629 | 3.412e-11 | 1.572e-09 | PPH3 | Phenotypic enhancement | cdc13-1 | | YPL179W | -0.7868 | 0.11990 | -6.560 | 5.413e-11 | 2.391e-09 | PPQ1 | Phenotypic enhancement | cdc13-1 | | YJR097W | -0.7838 | 0.11990 | -6.535 | 6.408e-11 | 2.802e-09 | JJJ3 | Phenotypic enhancement | cdc13-1 | | YCL016C | -0.7757 | 0.12410 | -6.248 | 4.176e-10 | 1.543e-08 | DCC1 | Phenotypic enhancement | cdc13-1 | | YGR187C | -0.7666 | 0.11990 | -6.392 | 1.647e-10 | 6.417e-09 | HGH1 | Phenotypic enhancement | cdc13-1 | | YBR034C | -0.7621 | 0.11990 | -6.354 | 2.115e-10 | 8.018e-09 | HMT1 | Phenotypic enhancement | cdc13-1 | | YJR043C | -0.7469 | 0.11990 | -6.227 | 4.775e-10 | 1.734e-08 | POL32 | Phenotypic enhancement | cdc13-1 | | YNL120C | -0.7345 | 0.11990 | -6.124 | 9.178e-10 | 3.198e-08 | \_ | Phenotypic enhancement | cdc13-1 | | YNL171C | -0.7327 | 0.12950 | -5.656 | 1.554e-08 | 4.296e-07 | \_ | Phenotypic enhancement | cdc13-1 | | YMR299C | -0.7054 | 0.11990 | -5.881 | 4.094e-09 | 1.262e-07 | DYN3 | Phenotypic enhancement | cdc13-1 | | YNL153C | -0.7051 | 0.11990 | -5.879 | 4.141e-09 | 1.268e-07 | GIM3 | Phenotypic enhancement | cdc13-1 | | YHL023C | -0.7038 | 0.11990 | -5.868 | 4.436e-09 | 1.348e-07 | NPR3 | Phenotypic enhancement | cdc13-1 | | YDR290W | -0.6887 | 0.12410 | -5.547 | 2.912e-08 | 7.655e-07 | \_ | Phenotypic enhancement | cdc13-1 | | YIL131C | -0.6874 | 0.11990 | -5.731 | 1.001e-08 | 2.839e-07 | FKH1 | Phenotypic enhancement | cdc13-1 | | YLR384C | -0.6873 | 0.11990 | -5.730 | 1.007e-08 | 2.839e-07 | IKI3 | Phenotypic enhancement | cdc13-1 | | YCR077C | -0.6869 | 0.11990 | -5.727 | 1.028e-08 | 2.878e-07 | PAT1 | Phenotypic enhancement | cdc13-1 | | YDL236W | -0.6807 | 0.11990 | -5.675 | 1.391e-08 | 3.870e-07 | PHO13 | Phenotypic enhancement | cdc13-1 | | YDR121W | -0.6748 | 0.12410 | -5.435 | 5.489e-08 | 1.329e-06 | DPB4 | Phenotypic enhancement | cdc13-1 | | YKL056C | -0.6731 | 0.11990 | -5.612 | 2.003e-08 | 5.431e-07 | TMA19 | Phenotypic enhancement | cdc13-1 | | YER089C | -0.6726 | 0.11990 | -5.608 | 2.055e-08 | 5.538e-07 | PTC2 | Phenotypic enhancement | cdc13-1 | | YIL153W | -0.6646 | 0.12410 | -5.353 | 8.676e-08 | 1.967e-06 | RRD1 | Phenotypic enhancement | cdc13-1 | | YDR477W | -0.6621 | 0.12950 | -5.111 | 3.216e-07 | 6.208e-06 | SNF1 | Phenotypic enhancement | cdc13-1 | | YGL127C | -0.6601 | 0.12950 | -5.096 | 3.481e-07 | 6.600e-06 | SOH1 | Phenotypic enhancement | cdc13-1 | | YDL056W | -0.6542 | 0.11990 | -5.454 | 4.934e-08 | 1.208e-06 | MBP1 | Phenotypic enhancement | cdc13-1 | | YPR051W | -0.6510 | 0.11990 | -5.428 | 5.722e-08 | 1.378e-06 | MAK3 | Phenotypic enhancement | cdc13-1 | | YNL140C | -0.6428 | 0.12410 | -5.178 | 2.247e-07 | 4.620e-06 | \_ | Phenotypic enhancement | cdc13-1 | | YHR041C | -0.6425 | 0.11990 | -5.357 | 8.492e-08 | 1.935e-06 | SRB2 | Phenotypic enhancement | cdc13-1 | | YMR074C | -0.6373 | 0.12410 | -5.133 | 2.857e-07 | 5.598e-06 | \_ | Phenotypic enhancement | cdc13-1 | | YKL149C | -0.6348 | 0.11990 | -5.293 | 1.207e-07 | 2.640e-06 | DBR1 | Phenotypic enhancement | cdc13-1 | | YPR040W | -0.6337 | 0.12410 | -5.105 | 3.324e-07 | 6.330e-06 | TIP41 | Phenotypic enhancement | cdc13-1 | | YJL168C | -0.6319 | 0.11990 | -5.269 | 1.378e-07 | 2.967e-06 | SET2 | Phenotypic enhancement | cdc13-1 | | YPL184C | -0.6309 | 0.11990 | -5.260 | 1.445e-07 | 3.096e-06 | MRN1 | Phenotypic enhancement | cdc13-1 | | YOL081W | -0.6275 | 0.12410 | -5.055 | 4.319e-07 | 8.047e-06 | IRA2 | Phenotypic enhancement | cdc13-1 | | YPL101W | -0.6254 | 0.11990 | -5.215 | 1.847e-07 | 3.880e-06 | ELP4 | Phenotypic enhancement | cdc13-1 | | YKR074W | -0.6217 | 0.11990 | -5.183 | 2.187e-07 | 4.549e-06 | AIM29 | Phenotypic enhancement | cdc13-1 | | YOR123C | -0.6210 | 0.11990 | -5.178 | 2.253e-07 | 4.620e-06 | LEO1 | Phenotypic enhancement | cdc13-1 | | YBR093C | -0.6195 | 0.12950 | -4.782 | 1.740e-06 | 2.772e-05 | PHO5 | Phenotypic enhancement | cdc13-1 | | YPR135W | -0.6163 | 0.11990 | -5.138 | 2.781e-07 | 5.562e-06 | CTF4 | Phenotypic enhancement | cdc13-1 | | YJR084W | -0.6159 | 0.11990 | -5.135 | 2.831e-07 | 5.598e-06 | CSN12 | Phenotypic enhancement | cdc13-1 | | YBL025W | -0.6126 | 0.12410 | -4.935 | 8.049e-07 | 1.385e-05 | RRN10 | Phenotypic enhancement | cdc13-1 | | YJL095W | -0.6125 | 0.11990 | -5.107 | 3.278e-07 | 6.270e-06 | BCK1 | Phenotypic enhancement | cdc13-1 | | YLL043W | -0.6093 | 0.11990 | -5.080 | 3.780e-07 | 7.073e-06 | FPS1 | Phenotypic enhancement | cdc13-1 | | YBR059C | -0.6056 | 0.11990 | -5.050 | 4.440e-07 | 8.201e-06 | AKL1 | Phenotypic enhancement | cdc13-1 | | YKR054C | -0.6052 | 0.11990 | -5.046 | 4.518e-07 | 8.280e-06 | DYN1 | Phenotypic enhancement | cdc13-1 | | YHR129C | -0.6051 | 0.12410 | -4.874 | 1.096e-06 | 1.828e-05 | ARP1 | Phenotypic enhancement | cdc13-1 | | YNL068C | -0.6050 | 0.11990 | -5.045 | 4.558e-07 | 8.280e-06 | FKH2 | Phenotypic enhancement | cdc13-1 | | YMR048W | -0.6031 | 0.11990 | -5.029 | 4.948e-07 | 8.908e-06 | CSM3 | Phenotypic enhancement | cdc13-1 | | YHR030C | -0.6027 | 0.11990 | -5.025 | 5.045e-07 | 9.045e-06 | SLT2 | Phenotypic enhancement | cdc13-1 | | YLR102C | -0.6024 | 0.12410 | -4.852 | 1.223e-06 | 2.016e-05 | APC9 | Phenotypic enhancement | cdc13-1 | | YPL102C | -0.5923 | 0.11990 | -4.938 | 7.909e-07 | 1.372e-05 | \_ | Phenotypic enhancement | cdc13-1 | | YPR039W | -0.5921 | 0.11990 | -4.937 | 7.960e-07 | 1.375e-05 | \_ | Phenotypic enhancement | cdc13-1 | | YBR277C | -0.5755 | 0.11990 | -4.798 | 1.604e-06 | 2.584e-05 | \_ | Phenotypic enhancement | cdc13-1 | | YKR082W | -0.5739 | 0.11990 | -4.785 | 1.716e-06 | 2.743e-05 | NUP133 | Phenotypic enhancement | cdc13-1 | | YPR031W | -0.5704 | 0.11990 | -4.756 | 1.979e-06 | 3.084e-05 | NTO1 | Phenotypic enhancement | cdc13-1 | | YGL244W | -0.5625 | 0.12410 | -4.531 | 5.887e-06 | 8.353e-05 | RTF1 | Phenotypic enhancement | cdc13-1 | | YDR369C | -0.5555 | 0.11990 | -4.632 | 3.629e-06 | 5.381e-05 | XRS2 | Phenotypic enhancement | cdc13-1 | | YMR055C | -0.5551 | 0.11990 | -4.628 | 3.698e-06 | 5.464e-05 | BUB2 | Phenotypic enhancement | cdc13-1 | | YOR144C | -0.5534 | 0.11990 | -4.614 | 3.954e-06 | 5.823e-05 | ELG1 | Phenotypic enhancement | cdc13-1 | | YGR157W | -0.5488 | 0.12950 | -4.236 | 2.279e-05 | 2.819e-04 | CHO2 | Phenotypic enhancement | cdc13-1 | | YNL280C | -0.5479 | 0.11990 | -4.568 | 4.936e-06 | 7.146e-05 | ERG24 | Phenotypic enhancement | cdc13-1 | | YPR023C | -0.5416 | 0.11990 | -4.515 | 6.328e-06 | 8.891e-05 | EAF3 | Phenotypic enhancement | cdc13-1 | | YDR378C | -0.5278 | 0.13670 | -3.859 | 1.138e-04 | 1.169e-03 | LSM6 | Phenotypic enhancement | cdc13-1 | | YPR050C | -0.5212 | 0.11990 | -4.346 | 1.390e-05 | 1.844e-04 | \_ | Phenotypic enhancement | cdc13-1 | | YHL034C | -0.5205 | 0.11990 | -4.340 | 1.430e-05 | 1.886e-04 | SBP1 | Phenotypic enhancement | cdc13-1 | | YCR053W | -0.5193 | 0.11990 | -4.330 | 1.495e-05 | 1.947e-04 | THR4 | Phenotypic enhancement | cdc13-1 | | YGL094C | -0.5187 | 0.11990 | -4.325 | 1.529e-05 | 1.985e-04 | PAN2 | Phenotypic enhancement | cdc13-1 | | YNL164C | -0.5181 | 0.11990 | -4.320 | 1.566e-05 | 2.021e-04 | IBD2 | Phenotypic enhancement | cdc13-1 | | YLR143W | -0.5174 | 0.12410 | -4.167 | 3.084e-05 | 3.671e-04 | \_ | Phenotypic enhancement | cdc13-1 | | YIL154C | -0.5151 | 0.11990 | -4.294 | 1.754e-05 | 2.224e-04 | IMP2' | Phenotypic enhancement | cdc13-1 | | YBR278W | -0.5128 | 0.11990 | -4.275 | 1.912e-05 | 2.396e-04 | DPB3 | Phenotypic enhancement | cdc13-1 | | YBL071C | -0.5080 | 0.11990 | -4.236 | 2.283e-05 | 2.819e-04 | \_ | Phenotypic enhancement | cdc13-1 | | YPR098C | -0.5051 | 0.11990 | -4.211 | 2.541e-05 | 3.129e-04 | \_ | Phenotypic enhancement | cdc13-1 | | YBR025C | -0.5048 | 0.11990 | -4.209 | 2.571e-05 | 3.156e-04 | OLA1 | Phenotypic enhancement | cdc13-1 | | YMR078C | -0.5013 | 0.11990 | -4.180 | 2.919e-05 | 3.513e-04 | CTF18 | Phenotypic enhancement | cdc13-1 | | YGR081C | -0.5006 | 0.11990 | -4.174 | 2.997e-05 | 3.597e-04 | SLX9 | Phenotypic enhancement | cdc13-1 | | YCR065W | 0.5004 | 0.11990 | 4.172 | 3.022e-05 | 3.617e-04 | HCM1 | Phenotypic suppression | cdc13-1 | | YOL049W | 0.5018 | 0.12410 | 4.042 | 5.302e-05 | 5.917e-04 | GSH2 | Phenotypic suppression | cdc13-1 | | YNL032W | 0.5019 | 0.12410 | 4.043 | 5.287e-05 | 5.915e-04 | SIW14 | Phenotypic suppression | cdc13-1 | | YJL004C | 0.5027 | 0.11990 | 4.192 | 2.773e-05 | 3.347e-04 | SYS1 | Phenotypic suppression | cdc13-1 | | YMR256C | 0.5029 | 0.11990 | 4.193 | 2.759e-05 | 3.340e-04 | COX7 | Phenotypic suppression | cdc13-1 | | YNL238W | 0.5029 | 0.11990 | 4.193 | 2.752e-05 | 3.340e-04 | KEX2 | Phenotypic suppression | cdc13-1 | | YDL118W | 0.5045 | 0.11990 | 4.207 | 2.594e-05 | 3.166e-04 | \_ | Phenotypic suppression | cdc13-1 | | YBL089W | 0.5046 | 0.11990 | 4.207 | 2.588e-05 | 3.166e-04 | AVT5 | Phenotypic suppression | cdc13-1 | | YLR398C | 0.5073 | 0.12410 | 4.087 | 4.384e-05 | 4.996e-04 | SKI2 | Phenotypic suppression | cdc13-1 | | YIL034C | 0.5131 | 0.11990 | 4.278 | 1.891e-05 | 2.376e-04 | CAP2 | Phenotypic suppression | cdc13-1 | | YJR070C | 0.5135 | 0.11990 | 4.281 | 1.862e-05 | 2.347e-04 | LIA1 | Phenotypic suppression | cdc13-1 | | YDR007W | 0.5144 | 0.11990 | 4.289 | 1.800e-05 | 2.275e-04 | TRP1 | Phenotypic suppression | cdc13-1 | | YMR193C-A | 0.5152 | 0.11990 | 4.296 | 1.744e-05 | 2.218e-04 | \_ | Phenotypic suppression | cdc13-1 | | YDL006W | 0.5163 | 0.11990 | 4.305 | 1.673e-05 | 2.146e-04 | PTC1 | Phenotypic suppression | cdc13-1 | | YEL037C | 0.5178 | 0.11990 | 4.318 | 1.579e-05 | 2.032e-04 | RAD23 | Phenotypic suppression | cdc13-1 | | YLR062C | 0.5182 | 0.11990 | 4.320 | 1.560e-05 | 2.019e-04 | BUD28 | Phenotypic suppression | cdc13-1 | | YER056C | 0.5195 | 0.11990 | 4.331 | 1.485e-05 | 1.940e-04 | FCY2 | Phenotypic suppression | cdc13-1 | | YDL142C | 0.5196 | 0.11990 | 4.332 | 1.479e-05 | 1.938e-04 | CRD1 | Phenotypic suppression | cdc13-1 | | YGR183C | 0.5213 | 0.11990 | 4.347 | 1.384e-05 | 1.842e-04 | QCR9 | Phenotypic suppression | cdc13-1 | | YOL009C | 0.5246 | 0.11990 | 4.374 | 1.221e-05 | 1.640e-04 | MDM12 | Phenotypic suppression | cdc13-1 | | YJL206C | 0.5271 | 0.12410 | 4.246 | 2.182e-05 | 2.710e-04 | \_ | Phenotypic suppression | cdc13-1 | | YMR067C | 0.5288 | 0.11990 | 4.409 | 1.041e-05 | 1.407e-04 | UBX4 | Phenotypic suppression | cdc13-1 | | YKR035W-A | 0.5338 | 0.12950 | 4.120 | 3.790e-05 | 4.425e-04 | DID2 | Phenotypic suppression | cdc13-1 | | YBL016W | 0.5342 | 0.11990 | 4.454 | 8.444e-06 | 1.145e-04 | FUS3 | Phenotypic suppression | cdc13-1 | | YBR291C | 0.5351 | 0.11990 | 4.462 | 8.144e-06 | 1.108e-04 | CTP1 | Phenotypic suppression | cdc13-1 | | YLR038C | 0.5357 | 0.11990 | 4.467 | 7.950e-06 | 1.085e-04 | COX12 | Phenotypic suppression | cdc13-1 | | YDR067C | 0.5380 | 0.12410 | 4.334 | 1.469e-05 | 1.931e-04 | OCA6 | Phenotypic suppression | cdc13-1 | | YGR174C | 0.5387 | 0.11990 | 4.492 | 7.071e-06 | 9.743e-05 | CBP4 | Phenotypic suppression | cdc13-1 | | YGR227W | 0.5391 | 0.12410 | 4.342 | 1.412e-05 | 1.868e-04 | DIE2 | Phenotypic suppression | cdc13-1 | | YKR042W | 0.5407 | 0.12410 | 4.355 | 1.331e-05 | 1.777e-04 | UTH1 | Phenotypic suppression | cdc13-1 | | YKL010C | 0.5411 | 0.11990 | 4.512 | 6.448e-06 | 8.995e-05 | UFD4 | Phenotypic suppression | cdc13-1 | | YGL213C | 0.5413 | 0.12410 | 4.360 | 1.300e-05 | 1.741e-04 | SKI8 | Phenotypic suppression | cdc13-1 | | YIL161W | 0.5419 | 0.11990 | 4.518 | 6.246e-06 | 8.804e-05 | \_ | Phenotypic suppression | cdc13-1 | | YER153C | 0.5435 | 0.12410 | 4.378 | 1.198e-05 | 1.615e-04 | PET122 | Phenotypic suppression | cdc13-1 | | YIL159W | 0.5444 | 0.11990 | 4.539 | 5.668e-06 | 8.068e-05 | BNR1 | Phenotypic suppression | cdc13-1 | | YGR148C | 0.5469 | 0.11990 | 4.560 | 5.119e-06 | 7.360e-05 | RPL24B | Phenotypic suppression | cdc13-1 | | YIL124W | 0.5471 | 0.11990 | 4.562 | 5.086e-06 | 7.338e-05 | AYR1 | Phenotypic suppression | cdc13-1 | | YGL124C | 0.5517 | 0.11990 | 4.600 | 4.235e-06 | 6.172e-05 | MON1 | Phenotypic suppression | cdc13-1 | | YHR146W | 0.5526 | 0.11990 | 4.608 | 4.082e-06 | 5.970e-05 | CRP1 | Phenotypic suppression | cdc13-1 | | YMR194W | 0.5564 | 0.11990 | 4.639 | 3.506e-06 | 5.216e-05 | RPL36A | Phenotypic suppression | cdc13-1 | | YDL232W | 0.5565 | 0.12950 | 4.296 | 1.744e-05 | 2.218e-04 | OST4 | Phenotypic suppression | cdc13-1 | | YHR178W | 0.5566 | 0.12410 | 4.483 | 7.370e-06 | 1.009e-04 | STB5 | Phenotypic suppression | cdc13-1 | | YPL079W | 0.5566 | 0.11990 | 4.641 | 3.476e-06 | 5.190e-05 | RPL21B | Phenotypic suppression | cdc13-1 | | YGL084C | 0.5568 | 0.12410 | 4.485 | 7.311e-06 | 1.004e-04 | GUP1 | Phenotypic suppression | cdc13-1 | | YLR289W | 0.5585 | 0.11990 | 4.656 | 3.223e-06 | 4.829e-05 | GUF1 | Phenotypic suppression | cdc13-1 | | YML001W | 0.5586 | 0.12410 | 4.500 | 6.816e-06 | 9.422e-05 | YPT7 | Phenotypic suppression | cdc13-1 | | YKL041W | 0.5604 | 0.12410 | 4.514 | 6.382e-06 | 8.936e-05 | VPS24 | Phenotypic suppression | cdc13-1 | | YIL053W | 0.5617 | 0.11990 | 4.683 | 2.833e-06 | 4.274e-05 | RHR2 | Phenotypic suppression | cdc13-1 | | YBR227C | 0.5620 | 0.12410 | 4.527 | 5.984e-06 | 8.462e-05 | MCX1 | Phenotypic suppression | cdc13-1 | | YJL101C | 0.5637 | 0.12410 | 4.541 | 5.616e-06 | 8.022e-05 | GSH1 | Phenotypic suppression | cdc13-1 | | YOR209C | 0.5642 | 0.11990 | 4.705 | 2.549e-06 | 3.873e-05 | NPT1 | Phenotypic suppression | cdc13-1 | | YJR094W-A | 0.5643 | 0.11990 | 4.705 | 2.545e-06 | 3.873e-05 | RPL43B | Phenotypic suppression | cdc13-1 | | YDL106C | 0.5658 | 0.11990 | 4.718 | 2.389e-06 | 3.670e-05 | PHO2 | Phenotypic suppression | cdc13-1 | | YAL026C | 0.5687 | 0.11990 | 4.741 | 2.128e-06 | 3.291e-05 | DRS2 | Phenotypic suppression | cdc13-1 | | YPL080C | 0.5696 | 0.11990 | 4.749 | 2.044e-06 | 3.174e-05 | \_ | Phenotypic suppression | cdc13-1 | | YIL123W | 0.5698 | 0.12410 | 4.590 | 4.439e-06 | 6.448e-05 | SIM1 | Phenotypic suppression | cdc13-1 | | YGR092W | 0.5707 | 0.11990 | 4.758 | 1.956e-06 | 3.059e-05 | DBF2 | Phenotypic suppression | cdc13-1 | | YLR335W | 0.5708 | 0.11990 | 4.759 | 1.945e-06 | 3.053e-05 | NUP2 | Phenotypic suppression | cdc13-1 | | YGL219C | 0.5727 | 0.12410 | 4.613 | 3.971e-06 | 5.828e-05 | MDM34 | Phenotypic suppression | cdc13-1 | | YML060W | 0.5732 | 0.11990 | 4.779 | 1.764e-06 | 2.790e-05 | OGG1 | Phenotypic suppression | cdc13-1 | | YKL184W | 0.5763 | 0.11990 | 4.805 | 1.548e-06 | 2.504e-05 | SPE1 | Phenotypic suppression | cdc13-1 | | YFR032C-A | 0.5770 | 0.11990 | 4.811 | 1.508e-06 | 2.448e-05 | RPL29 | Phenotypic suppression | cdc13-1 | | YHR116W | 0.5772 | 0.11990 | 4.813 | 1.493e-06 | 2.436e-05 | COX23 | Phenotypic suppression | cdc13-1 | | YMR057C | 0.5775 | 0.11990 | 4.815 | 1.474e-06 | 2.420e-05 | \_ | Phenotypic suppression | cdc13-1 | | YIL057C | 0.5806 | 0.12410 | 4.677 | 2.923e-06 | 4.395e-05 | \_ | Phenotypic suppression | cdc13-1 | | YDL190C | 0.5824 | 0.13670 | 4.259 | 2.055e-05 | 2.560e-04 | UFD2 | Phenotypic suppression | cdc13-1 | | YIL079C | 0.5878 | 0.12410 | 4.735 | 2.195e-06 | 3.384e-05 | AIR1 | Phenotypic suppression | cdc13-1 | | YCR071C | 0.5885 | 0.11990 | 4.907 | 9.268e-07 | 1.557e-05 | IMG2 | Phenotypic suppression | cdc13-1 | | YJR040W | 0.5887 | 0.11990 | 4.908 | 9.211e-07 | 1.554e-05 | GEF1 | Phenotypic suppression | cdc13-1 | | YOL052C | 0.5890 | 0.11990 | 4.911 | 9.082e-07 | 1.538e-05 | SPE2 | Phenotypic suppression | cdc13-1 | | YER020W | 0.5900 | 0.11990 | 4.919 | 8.717e-07 | 1.482e-05 | GPA2 | Phenotypic suppression | cdc13-1 | | YLR354C | 0.5907 | 0.11990 | 4.925 | 8.453e-07 | 1.443e-05 | TAL1 | Phenotypic suppression | cdc13-1 | | YJL036W | 0.5911 | 0.11990 | 4.928 | 8.307e-07 | 1.424e-05 | SNX4 | Phenotypic suppression | cdc13-1 | | YLR193C | 0.5922 | 0.12410 | 4.770 | 1.847e-06 | 2.909e-05 | UPS1 | Phenotypic suppression | cdc13-1 | | YAR018C | 0.5925 | 0.11990 | 4.940 | 7.837e-07 | 1.365e-05 | KIN3 | Phenotypic suppression | cdc13-1 | | YGL202W | 0.5977 | 0.11990 | 4.983 | 6.261e-07 | 1.099e-05 | ARO8 | Phenotypic suppression | cdc13-1 | | YNL162W | 0.6002 | 0.11990 | 5.004 | 5.617e-07 | 9.946e-06 | RPL42A | Phenotypic suppression | cdc13-1 | | YER145C | 0.6016 | 0.11990 | 5.016 | 5.296e-07 | 9.455e-06 | FTR1 | Phenotypic suppression | cdc13-1 | | YDL117W | 0.6036 | 0.11990 | 5.033 | 4.845e-07 | 8.759e-06 | CYK3 | Phenotypic suppression | cdc13-1 | | YIL017C | 0.6044 | 0.12410 | 4.869 | 1.126e-06 | 1.871e-05 | VID28 | Phenotypic suppression | cdc13-1 | | YLR402W | 0.6050 | 0.11990 | 5.044 | 4.560e-07 | 8.280e-06 | \_ | Phenotypic suppression | cdc13-1 | | YIL160C | 0.6051 | 0.11990 | 5.045 | 4.548e-07 | 8.280e-06 | POT1 | Phenotypic suppression | cdc13-1 | | YMR058W | 0.6061 | 0.11990 | 5.054 | 4.345e-07 | 8.059e-06 | FET3 | Phenotypic suppression | cdc13-1 | | YFL025C | 0.6128 | 0.11990 | 5.109 | 3.240e-07 | 6.226e-06 | BST1 | Phenotypic suppression | cdc13-1 | | YBL047C | 0.6137 | 0.11990 | 5.117 | 3.118e-07 | 6.046e-06 | EDE1 | Phenotypic suppression | cdc13-1 | | YBR066C | 0.6152 | 0.11990 | 5.129 | 2.917e-07 | 5.682e-06 | NRG2 | Phenotypic suppression | cdc13-1 | | YLR184W | 0.6156 | 0.11990 | 5.133 | 2.861e-07 | 5.598e-06 | \_ | Phenotypic suppression | cdc13-1 | | YBR274W | 0.6158 | 0.04575 | 13.460 | 0.000e+00 | 0.000e+00 | CHK1 | Phenotypic suppression | cdc13-1 | | YNL080C | 0.6158 | 0.11990 | 5.134 | 2.840e-07 | 5.598e-06 | EOS1 | Phenotypic suppression | cdc13-1 | | YDR127W | 0.6167 | 0.13670 | 4.510 | 6.504e-06 | 9.019e-05 | ARO1 | Phenotypic suppression | cdc13-1 | | YCR034W | 0.6173 | 0.11990 | 5.147 | 2.651e-07 | 5.333e-06 | FEN1 | Phenotypic suppression | cdc13-1 | | YNL227C | 0.6192 | 0.12950 | 4.780 | 1.761e-06 | 2.790e-05 | JJJ1 | Phenotypic suppression | cdc13-1 | | YGL023C | 0.6192 | 0.11990 | 5.163 | 2.438e-07 | 4.950e-06 | PIB2 | Phenotypic suppression | cdc13-1 | | YGL218W | 0.6203 | 0.12950 | 4.789 | 1.684e-06 | 2.702e-05 | \_ | Phenotypic suppression | cdc13-1 | | YIL133C | 0.6212 | 0.11990 | 5.180 | 2.228e-07 | 4.612e-06 | RPL16A | Phenotypic suppression | cdc13-1 | | YNL241C | 0.6222 | 0.11990 | 5.188 | 2.132e-07 | 4.456e-06 | ZWF1 | Phenotypic suppression | cdc13-1 | | YGL031C | 0.6288 | 0.11990 | 5.243 | 1.584e-07 | 3.359e-06 | RPL24A | Phenotypic suppression | cdc13-1 | | YDR206W | 0.6314 | 0.12410 | 5.086 | 3.667e-07 | 6.892e-06 | EBS1 | Phenotypic suppression | cdc13-1 | | YBL104C | 0.6340 | 0.12950 | 4.894 | 9.919e-07 | 1.660e-05 | \_ | Phenotypic suppression | cdc13-1 | | YAR014C | 0.6366 | 0.11990 | 5.308 | 1.114e-07 | 2.460e-06 | BUD14 | Phenotypic suppression | cdc13-1 | | YPR045C | 0.6391 | 0.12410 | 5.148 | 2.641e-07 | 5.333e-06 | \_ | Phenotypic suppression | cdc13-1 | | YIL055C | 0.6394 | 0.11990 | 5.331 | 9.785e-08 | 2.207e-06 | \_ | Phenotypic suppression | cdc13-1 | | YDR217C | 0.6408 | 0.04533 | 14.140 | 0.000e+00 | 0.000e+00 | RAD9 | Phenotypic suppression | cdc13-1 | | YDR388W | 0.6458 | 0.11990 | 5.385 | 7.289e-08 | 1.679e-06 | RVS167 | Phenotypic suppression | cdc13-1 | | YBL007C | 0.6475 | 0.11990 | 5.398 | 6.751e-08 | 1.572e-06 | SLA1 | Phenotypic suppression | cdc13-1 | | YLR110C | 0.6481 | 0.12410 | 5.221 | 1.789e-07 | 3.777e-06 | CCW12 | Phenotypic suppression | cdc13-1 | | YLR111W | 0.6486 | 0.11990 | 5.407 | 6.414e-08 | 1.510e-06 | \_ | Phenotypic suppression | cdc13-1 | | YIL107C | 0.6495 | 0.11990 | 5.416 | 6.132e-08 | 1.468e-06 | PFK26 | Phenotypic suppression | cdc13-1 | | YLL045C | 0.6539 | 0.11990 | 5.452 | 4.990e-08 | 1.215e-06 | RPL8B | Phenotypic suppression | cdc13-1 | | YML010C-B | 0.6545 | 0.12410 | 5.272 | 1.355e-07 | 2.932e-06 |  | Phenotypic suppression | cdc13-1 | | YCR028C-A | 0.6545 | 0.11990 | 5.457 | 4.856e-08 | 1.196e-06 | RIM1 | Phenotypic suppression | cdc13-1 | | YDR471W | 0.6548 | 0.11990 | 5.459 | 4.799e-08 | 1.189e-06 | RPL27B | Phenotypic suppression | cdc13-1 | | YLR453C | 0.6557 | 0.11990 | 5.467 | 4.592e-08 | 1.144e-06 | RIF2 | Phenotypic suppression | cdc13-1 | | YBR057C | 0.6561 | 0.11990 | 5.470 | 4.514e-08 | 1.131e-06 | MUM2 | Phenotypic suppression | cdc13-1 | | YMR039C | 0.6583 | 0.11990 | 5.488 | 4.072e-08 | 1.026e-06 | SUB1 | Phenotypic suppression | cdc13-1 | | YLR338W | 0.6583 | 0.11990 | 5.488 | 4.072e-08 | 1.026e-06 | OPI9 | Phenotypic suppression | cdc13-1 | | YGR085C | 0.6591 | 0.12410 | 5.309 | 1.106e-07 | 2.456e-06 | RPL11B | Phenotypic suppression | cdc13-1 | | YBR267W | 0.6595 | 0.12410 | 5.312 | 1.087e-07 | 2.425e-06 | REI1 | Phenotypic suppression | cdc13-1 | | YPL069C | 0.6656 | 0.12950 | 5.138 | 2.791e-07 | 5.562e-06 | BTS1 | Phenotypic suppression | cdc13-1 | | YLR372W | 0.6671 | 0.11990 | 5.562 | 2.672e-08 | 7.067e-07 | SUR4 | Phenotypic suppression | cdc13-1 | | YGL024W | 0.6681 | 0.11990 | 5.570 | 2.550e-08 | 6.787e-07 | \_ | Phenotypic suppression | cdc13-1 | | YFL036W | 0.6706 | 0.12950 | 5.177 | 2.264e-07 | 4.621e-06 | RPO41 | Phenotypic suppression | cdc13-1 | | YLR337C | 0.6707 | 0.12410 | 5.403 | 6.589e-08 | 1.543e-06 | VRP1 | Phenotypic suppression | cdc13-1 | | YLR448W | 0.6752 | 0.11990 | 5.630 | 1.811e-08 | 4.975e-07 | RPL6B | Phenotypic suppression | cdc13-1 | | YGR101W | 0.6907 | 0.11990 | 5.759 | 8.524e-09 | 2.435e-07 | PCP1 | Phenotypic suppression | cdc13-1 | | YKR020W | 0.6986 | 0.11990 | 5.824 | 5.756e-09 | 1.655e-07 | VPS51 | Phenotypic suppression | cdc13-1 | | YNL322C | 0.6989 | 0.11990 | 5.827 | 5.658e-09 | 1.648e-07 | KRE1 | Phenotypic suppression | cdc13-1 | | YPR044C | 0.7013 | 0.12950 | 5.414 | 6.195e-08 | 1.475e-06 | OPI11 | Phenotypic suppression | cdc13-1 | | YOR068C | 0.7016 | 0.11990 | 5.850 | 4.947e-09 | 1.472e-07 | VAM10 | Phenotypic suppression | cdc13-1 | | YIL128W | 0.7028 | 0.11990 | 5.860 | 4.648e-09 | 1.393e-07 | MET18 | Phenotypic suppression | cdc13-1 | | YOR039W | 0.7078 | 0.11990 | 5.901 | 3.628e-09 | 1.127e-07 | CKB2 | Phenotypic suppression | cdc13-1 | | YLR262C | 0.7111 | 0.11990 | 5.929 | 3.070e-09 | 9.603e-08 | YPT6 | Phenotypic suppression | cdc13-1 | | YKL009W | 0.7114 | 0.11990 | 5.931 | 3.022e-09 | 9.520e-08 | MRT4 | Phenotypic suppression | cdc13-1 | | YLR261C | 0.7147 | 0.11990 | 5.959 | 2.557e-09 | 8.238e-08 | VPS63 | Phenotypic suppression | cdc13-1 | | YER002W | 0.7205 | 0.11990 | 6.007 | 1.897e-09 | 6.205e-08 | NOP16 | Phenotypic suppression | cdc13-1 | | YOL138C | 0.7215 | 0.11990 | 6.016 | 1.801e-09 | 5.983e-08 | RTC1 | Phenotypic suppression | cdc13-1 | | YDL191W | 0.7227 | 0.11990 | 6.026 | 1.692e-09 | 5.665e-08 | RPL35A | Phenotypic suppression | cdc13-1 | | YIL097W | 0.7238 | 0.12410 | 5.830 | 5.567e-09 | 1.634e-07 | FYV10 | Phenotypic suppression | cdc13-1 | | YPR043W | 0.7258 | 0.12950 | 5.603 | 2.119e-08 | 5.675e-07 | RPL43A | Phenotypic suppression | cdc13-1 | | YNR005C | 0.7276 | 0.12410 | 5.861 | 4.637e-09 | 1.393e-07 | \_ | Phenotypic suppression | cdc13-1 | | YLR061W | 0.7289 | 0.11990 | 6.077 | 1.229e-09 | 4.213e-08 | RPL22A | Phenotypic suppression | cdc13-1 | | YFR010W | 0.7309 | 0.11990 | 6.094 | 1.109e-09 | 3.832e-08 | UBP6 | Phenotypic suppression | cdc13-1 | | YLR039C | 0.7403 | 0.11990 | 6.172 | 6.771e-10 | 2.378e-08 | RIC1 | Phenotypic suppression | cdc13-1 | | YNL226W | 0.7426 | 0.12410 | 5.982 | 2.218e-09 | 7.200e-08 | \_ | Phenotypic suppression | cdc13-1 | | YLR185W | 0.7482 | 0.12410 | 6.027 | 1.683e-09 | 5.665e-08 | RPL37A | Phenotypic suppression | cdc13-1 | | YLR119W | 0.7490 | 0.11990 | 6.245 | 4.258e-10 | 1.560e-08 | SRN2 | Phenotypic suppression | cdc13-1 | | YDR382W | 0.7516 | 0.12410 | 6.054 | 1.420e-09 | 4.829e-08 | RPP2B | Phenotypic suppression | cdc13-1 | | YAL002W | 0.7634 | 0.11990 | 6.365 | 1.968e-10 | 7.530e-09 | VPS8 | Phenotypic suppression | cdc13-1 | | YOR035C | 0.7671 | 0.11990 | 6.396 | 1.603e-10 | 6.301e-09 | SHE4 | Phenotypic suppression | cdc13-1 | | YIL036W | 0.7706 | 0.11990 | 6.425 | 1.327e-10 | 5.364e-09 | CST6 | Phenotypic suppression | cdc13-1 | | YDL082W | 0.7725 | 0.12410 | 6.222 | 4.925e-10 | 1.774e-08 | RPL13A | Phenotypic suppression | cdc13-1 | | YIL110W | 0.7763 | 0.11990 | 6.472 | 9.720e-11 | 4.005e-09 | MNI1 | Phenotypic suppression | cdc13-1 | | YHR081W | 0.7788 | 0.12950 | 6.012 | 1.847e-09 | 6.089e-08 | LRP1 | Phenotypic suppression | cdc13-1 | | YDL136W | 0.7810 | 0.11990 | 6.512 | 7.490e-11 | 3.178e-09 | RPL35B | Phenotypic suppression | cdc13-1 | | YHR066W | 0.7835 | 0.11990 | 6.532 | 6.523e-11 | 2.823e-09 | SSF1 | Phenotypic suppression | cdc13-1 | | YGL078C | 0.7840 | 0.12410 | 6.315 | 2.720e-10 | 1.013e-08 | DBP3 | Phenotypic suppression | cdc13-1 | | YFR031C-A | 0.7869 | 0.11990 | 6.561 | 5.375e-11 | 2.391e-09 | RPL2A | Phenotypic suppression | cdc13-1 | | YPR173C | 0.7933 | 0.12410 | 6.390 | 1.672e-10 | 6.453e-09 | VPS4 | Phenotypic suppression | cdc13-1 | | YDR500C | 0.7935 | 0.11990 | 6.616 | 3.723e-11 | 1.697e-09 | RPL37B | Phenotypic suppression | cdc13-1 | | YGL212W | 0.7955 | 0.11990 | 6.633 | 3.316e-11 | 1.545e-09 | VAM7 | Phenotypic suppression | cdc13-1 | | YPR074C | 0.7968 | 0.11990 | 6.644 | 3.080e-11 | 1.450e-09 | TKL1 | Phenotypic suppression | cdc13-1 | | YIR002C | 0.7972 | 0.11990 | 6.647 | 3.013e-11 | 1.435e-09 | MPH1 | Phenotypic suppression | cdc13-1 | | YER007C-A | 0.7997 | 0.11990 | 6.668 | 2.614e-11 | 1.288e-09 | TMA20 | Phenotypic suppression | cdc13-1 | | YKR099W | 0.8076 | 0.11990 | 6.734 | 1.665e-11 | 8.491e-10 | BAS1 | Phenotypic suppression | cdc13-1 | | YML097C | 0.8078 | 0.11990 | 6.735 | 1.651e-11 | 8.491e-10 | VPS9 | Phenotypic suppression | cdc13-1 | | YGL147C | 0.8085 | 0.12410 | 6.513 | 7.429e-11 | 3.178e-09 | RPL9A | Phenotypic suppression | cdc13-1 | | YKL213C | 0.8219 | 0.11990 | 6.853 | 7.307e-12 | 3.993e-10 | DOA1 | Phenotypic suppression | cdc13-1 | | YKL048C | 0.8252 | 0.11990 | 6.880 | 6.025e-12 | 3.353e-10 | ELM1 | Phenotypic suppression | cdc13-1 | | YDL119C | 0.8257 | 0.11990 | 6.885 | 5.851e-12 | 3.299e-10 | \_ | Phenotypic suppression | cdc13-1 | | YER119C-A | 0.8304 | 0.11990 | 6.924 | 4.441e-12 | 2.572e-10 | \_ | Phenotypic suppression | cdc13-1 | | YJR014W | 0.8347 | 0.11990 | 6.959 | 3.456e-12 | 2.029e-10 | TMA22 | Phenotypic suppression | cdc13-1 | | YLR056W | 0.8385 | 0.11990 | 6.991 | 2.750e-12 | 1.637e-10 | ERG3 | Phenotypic suppression | cdc13-1 | | YGL148W | 0.8438 | 0.11990 | 7.035 | 2.010e-12 | 1.230e-10 | ARO2 | Phenotypic suppression | cdc13-1 | | YDR389W | 0.8453 | 0.12410 | 6.809 | 9.921e-12 | 5.248e-10 | SAC7 | Phenotypic suppression | cdc13-1 | | YNL307C | 0.8524 | 0.11990 | 7.107 | 1.196e-12 | 7.650e-11 | MCK1 | Phenotypic suppression | cdc13-1 | | YHL033C | 0.8560 | 0.12410 | 6.896 | 5.417e-12 | 3.095e-10 | RPL8A | Phenotypic suppression | cdc13-1 | | YDL130W | 0.8605 | 0.11990 | 7.175 | 7.327e-13 | 4.906e-11 | RPP1B | Phenotypic suppression | cdc13-1 | | YBR266C | 0.8624 | 0.11990 | 7.191 | 6.510e-13 | 4.428e-11 | SLM6 | Phenotypic suppression | cdc13-1 | | YDR101C | 0.8887 | 0.11990 | 7.410 | 1.279e-13 | 1.034e-11 | ARX1 | Phenotypic suppression | cdc13-1 | | YFL023W | 0.9123 | 0.11990 | 7.606 | 2.864e-14 | 2.557e-12 | BUD27 | Phenotypic suppression | cdc13-1 | | YKR007W | 0.9216 | 0.11990 | 7.684 | 1.554e-14 | 1.417e-12 | MEH1 | Phenotypic suppression | cdc13-1 | | YOR061W | 0.9415 | 0.11990 | 7.850 | 4.219e-15 | 4.304e-13 | CKA2 | Phenotypic suppression | cdc13-1 | | YBR082C | 0.9445 | 0.08481 | 11.140 | 0.000e+00 | 0.000e+00 | UBC4 | Phenotypic suppression | cdc13-1 | | YER173W | 0.9470 | 0.04575 | 20.700 | 0.000e+00 | 0.000e+00 | RAD24 | Phenotypic suppression | cdc13-1 | | YHR077C | 0.9744 | 0.04554 | 21.400 | 0.000e+00 | 0.000e+00 | UPF2 | Phenotypic suppression | cdc13-1 | | YNL069C | 0.9763 | 0.11990 | 8.140 | 4.441e-16 | 5.437e-14 | RPL16B | Phenotypic suppression | cdc13-1 | | YOR368W | 0.9835 | 0.04533 | 21.690 | 0.000e+00 | 0.000e+00 | RAD17 | Phenotypic suppression | cdc13-1 | | YML121W | 0.9841 | 0.12410 | 7.927 | 2.220e-15 | 2.440e-13 | GTR1 | Phenotypic suppression | cdc13-1 | | YLR410W | 1.0040 | 0.11990 | 8.369 | 0.000e+00 | 0.000e+00 | VIP1 | Phenotypic suppression | cdc13-1 | | YER120W | 1.0100 | 0.11990 | 8.420 | 0.000e+00 | 0.000e+00 | SCS2 | Phenotypic suppression | cdc13-1 | | YOR033C | 1.0320 | 0.04533 | 22.770 | 0.000e+00 | 0.000e+00 | EXO1 | Phenotypic suppression | cdc13-1 | | YMR080C | 1.0370 | 0.11990 | 8.644 | 0.000e+00 | 0.000e+00 | UPF1 | Phenotypic suppression | cdc13-1 | | YGR072W | 1.0370 | 0.11990 | 8.648 | 0.000e+00 | 0.000e+00 | UPF3 | Phenotypic suppression | cdc13-1 | | YDR128W | 1.0370 | 0.11990 | 8.648 | 0.000e+00 | 0.000e+00 | MTC5 | Phenotypic suppression | cdc13-1 | | YPL194W | 1.0710 | 0.11990 | 8.931 | 0.000e+00 | 0.000e+00 | DDC1 | Phenotypic suppression | cdc13-1 | | YER177W | 1.0880 | 0.11990 | 9.073 | 0.000e+00 | 0.000e+00 | BMH1 | Phenotypic suppression | cdc13-1 | | YDL020C | 1.1000 | 0.11990 | 9.172 | 0.000e+00 | 0.000e+00 | RPN4 | Phenotypic suppression | cdc13-1 | | YDR143C | 1.1110 | 0.11990 | 9.260 | 0.000e+00 | 0.000e+00 | SAN1 | Phenotypic suppression | cdc13-1 | | YKL176C | 1.1970 | 0.12410 | 9.642 | 0.000e+00 | 0.000e+00 | LST4 | Phenotypic suppression | cdc13-1 | | YDR315C | 1.2410 | 0.11990 | 10.350 | 0.000e+00 | 0.000e+00 | IPK1 | Phenotypic suppression | cdc13-1 | |
