# Supplementary material for: Quantitative Fitness Analysis Shows That NMD Proteins and Many Other Protein Complexes Suppress or Enhance Distinct Telomere Cap Defects
Source: PLoS Genet. 2011 Apr 7;7(4):e1001362. doi: 10.1371/journal.pgen.1001362 (PMC3072368; doi:10.1371/journal.pgen.1001362)
Supplement: Table S9 — List of suppressors and enhancers of temperature-induced fitness defect at 37°C. A list of genes which, when deleted, result in significantly better or worse growth at 37°C compared to 20°C. Only included are gene deletions which passed a 5% FDR cutoff and had a GIS of greater than 0.5 (+ or −) in magnitude. http://research.ncl.ac.uk/colonyzer/AddinallQFA/S9_cSGA_37_20.html. See http://research.ncl.ac.uk/colonyzer/AddinallQFA for a list of all significant interactors, a GIS plot of these data and raw data. (0.09 MB HTML) [file pgen.1001362.s013.html]

Genetic interaction hitlist after QFA

Temperature Sensitivity cSGA

| | ORF | GIS | stderr | tval | pval | qval | genename | interaction | query | | --- | --- | --- | --- | --- | --- | --- | --- | --- | | YBR171W | -1.1184 | 0.06146 | -18.199 | 0.000e+00 | 0.000e+00 | SEC66 | Phenotypic enhancement | high temperature | | YFL054C | -1.0895 | 0.06146 | -17.728 | 0.000e+00 | 0.000e+00 | \_ | Phenotypic enhancement | high temperature | | YBR019C | -1.0879 | 0.06146 | -17.703 | 0.000e+00 | 0.000e+00 | GAL10 | Phenotypic enhancement | high temperature | | YGL110C | -1.0862 | 0.06146 | -17.675 | 0.000e+00 | 0.000e+00 | CUE3 | Phenotypic enhancement | high temperature | | YCR089W | -1.0776 | 0.06146 | -17.534 | 0.000e+00 | 0.000e+00 | FIG2 | Phenotypic enhancement | high temperature | | YER149C | -1.0759 | 0.06146 | -17.507 | 0.000e+00 | 0.000e+00 | PEA2 | Phenotypic enhancement | high temperature | | YGL081W | -1.0742 | 0.06146 | -17.479 | 0.000e+00 | 0.000e+00 | \_ | Phenotypic enhancement | high temperature | | YDR248C | -1.0737 | 0.06146 | -17.472 | 0.000e+00 | 0.000e+00 | \_ | Phenotypic enhancement | high temperature | | YDL155W | -1.0715 | 0.06146 | -17.435 | 0.000e+00 | 0.000e+00 | CLB3 | Phenotypic enhancement | high temperature | | YDL243C | -1.0618 | 0.06146 | -17.278 | 0.000e+00 | 0.000e+00 | AAD4 | Phenotypic enhancement | high temperature | | YGL196W | -1.0590 | 0.06146 | -17.232 | 0.000e+00 | 0.000e+00 | DSD1 | Phenotypic enhancement | high temperature | | YDR358W | -1.0582 | 0.06146 | -17.220 | 0.000e+00 | 0.000e+00 | GGA1 | Phenotypic enhancement | high temperature | | YNL087W | -1.0574 | 0.06146 | -17.205 | 0.000e+00 | 0.000e+00 | TCB2 | Phenotypic enhancement | high temperature | | YPL192C | -1.0572 | 0.06146 | -17.204 | 0.000e+00 | 0.000e+00 | PRM3 | Phenotypic enhancement | high temperature | | YPL244C | -1.0541 | 0.06146 | -17.152 | 0.000e+00 | 0.000e+00 | HUT1 | Phenotypic enhancement | high temperature | | YBR064W | -1.0496 | 0.06146 | -17.080 | 0.000e+00 | 0.000e+00 | \_ | Phenotypic enhancement | high temperature | | YGL101W | -1.0495 | 0.06146 | -17.078 | 0.000e+00 | 0.000e+00 | \_ | Phenotypic enhancement | high temperature | | YBL053W | -1.0475 | 0.06146 | -17.044 | 0.000e+00 | 0.000e+00 | \_ | Phenotypic enhancement | high temperature | | YBR111C | -1.0444 | 0.06146 | -16.994 | 0.000e+00 | 0.000e+00 | YSA1 | Phenotypic enhancement | high temperature | | YLR046C | -1.0432 | 0.06146 | -16.975 | 0.000e+00 | 0.000e+00 | \_ | Phenotypic enhancement | high temperature | | YPL103C | -1.0421 | 0.06146 | -16.957 | 0.000e+00 | 0.000e+00 | FMP30 | Phenotypic enhancement | high temperature | | YMR003W | -1.0412 | 0.06146 | -16.942 | 0.000e+00 | 0.000e+00 | AIM34 | Phenotypic enhancement | high temperature | | YOL054W | -1.0399 | 0.06146 | -16.922 | 0.000e+00 | 0.000e+00 | PSH1 | Phenotypic enhancement | high temperature | | YDR233C | -1.0358 | 0.06146 | -16.855 | 0.000e+00 | 0.000e+00 | RTN1 | Phenotypic enhancement | high temperature | | YDR260C | -1.0352 | 0.06146 | -16.845 | 0.000e+00 | 0.000e+00 | SWM1 | Phenotypic enhancement | high temperature | | YFR045W | -1.0329 | 0.06146 | -16.808 | 0.000e+00 | 0.000e+00 | \_ | Phenotypic enhancement | high temperature | | YDR440W | -1.0280 | 0.06146 | -16.729 | 0.000e+00 | 0.000e+00 | DOT1 | Phenotypic enhancement | high temperature | | YLR428C | -1.0257 | 0.06146 | -16.691 | 0.000e+00 | 0.000e+00 | \_ | Phenotypic enhancement | high temperature | | YDR133C | -1.0206 | 0.06146 | -16.607 | 0.000e+00 | 0.000e+00 | \_ | Phenotypic enhancement | high temperature | | YJL007C | -1.0197 | 0.06146 | -16.592 | 0.000e+00 | 0.000e+00 | \_ | Phenotypic enhancement | high temperature | | YPR053C | -1.0182 | 0.06146 | -16.568 | 0.000e+00 | 0.000e+00 | \_ | Phenotypic enhancement | high temperature | | YPR030W | -1.0176 | 0.06146 | -16.559 | 0.000e+00 | 0.000e+00 | CSR2 | Phenotypic enhancement | high temperature | | YBR030W | -1.0167 | 0.06146 | -16.543 | 0.000e+00 | 0.000e+00 | RKM3 | Phenotypic enhancement | high temperature | | YDL184C | -1.0142 | 0.06146 | -16.502 | 0.000e+00 | 0.000e+00 | RPL41A | Phenotypic enhancement | high temperature | | YAL053W | -1.0122 | 0.06146 | -16.470 | 0.000e+00 | 0.000e+00 | FLC2 | Phenotypic enhancement | high temperature | | YDR276C | -1.0059 | 0.06146 | -16.369 | 0.000e+00 | 0.000e+00 | PMP3 | Phenotypic enhancement | high temperature | | YIL076W | -1.0044 | 0.06146 | -16.344 | 0.000e+00 | 0.000e+00 | SEC28 | Phenotypic enhancement | high temperature | | YPL041C | -1.0000 | 0.06146 | -16.272 | 0.000e+00 | 0.000e+00 | \_ | Phenotypic enhancement | high temperature | | YOR084W | -1.0000 | 0.06146 | -16.272 | 0.000e+00 | 0.000e+00 | LPX1 | Phenotypic enhancement | high temperature | | YBL085W | -0.9961 | 0.06146 | -16.209 | 0.000e+00 | 0.000e+00 | BOI1 | Phenotypic enhancement | high temperature | | YDR314C | -0.9946 | 0.06146 | -16.185 | 0.000e+00 | 0.000e+00 | RAD34 | Phenotypic enhancement | high temperature | | YLR315W | -0.9918 | 0.06146 | -16.138 | 0.000e+00 | 0.000e+00 | NKP2 | Phenotypic enhancement | high temperature | | YGL109W | -0.9811 | 0.06146 | -15.965 | 0.000e+00 | 0.000e+00 | \_ | Phenotypic enhancement | high temperature | | YOR275C | -0.9789 | 0.06146 | -15.930 | 0.000e+00 | 0.000e+00 | RIM20 | Phenotypic enhancement | high temperature | | YKL066W | -0.9766 | 0.06146 | -15.892 | 0.000e+00 | 0.000e+00 | \_ | Phenotypic enhancement | high temperature | | YGL256W | -0.9719 | 0.06146 | -15.815 | 0.000e+00 | 0.000e+00 | ADH4 | Phenotypic enhancement | high temperature | | YOR251C | -0.9683 | 0.06146 | -15.757 | 0.000e+00 | 0.000e+00 | TUM1 | Phenotypic enhancement | high temperature | | YPR096C | -0.9672 | 0.06146 | -15.738 | 0.000e+00 | 0.000e+00 | \_ | Phenotypic enhancement | high temperature | | YPL256C | -0.9643 | 0.06146 | -15.691 | 0.000e+00 | 0.000e+00 | CLN2 | Phenotypic enhancement | high temperature | | YNR071C | -0.9593 | 0.06146 | -15.610 | 0.000e+00 | 0.000e+00 | \_ | Phenotypic enhancement | high temperature | | YKL177W | -0.9587 | 0.06146 | -15.600 | 0.000e+00 | 0.000e+00 | \_ | Phenotypic enhancement | high temperature | | YMR004W | -0.9570 | 0.06146 | -15.573 | 0.000e+00 | 0.000e+00 | MVP1 | Phenotypic enhancement | high temperature | | YJR035W | -0.9560 | 0.06146 | -15.556 | 0.000e+00 | 0.000e+00 | RAD26 | Phenotypic enhancement | high temperature | | YOR296W | -0.9537 | 0.06146 | -15.519 | 0.000e+00 | 0.000e+00 | \_ | Phenotypic enhancement | high temperature | | YDR508C | -0.9530 | 0.06146 | -15.507 | 0.000e+00 | 0.000e+00 | GNP1 | Phenotypic enhancement | high temperature | | YLR329W | -0.9509 | 0.06146 | -15.473 | 0.000e+00 | 0.000e+00 | REC102 | Phenotypic enhancement | high temperature | | YOR043W | -0.9503 | 0.06146 | -15.463 | 0.000e+00 | 0.000e+00 | WHI2 | Phenotypic enhancement | high temperature | | YDR385W | -0.9496 | 0.06146 | -15.453 | 0.000e+00 | 0.000e+00 | EFT2 | Phenotypic enhancement | high temperature | | YLR039C | -0.9477 | 0.06146 | -15.421 | 0.000e+00 | 0.000e+00 | RIC1 | Phenotypic enhancement | high temperature | | YMR052C-A | -0.9462 | 0.06146 | -15.397 | 0.000e+00 | 0.000e+00 | \_ | Phenotypic enhancement | high temperature | | YLR261C | -0.9440 | 0.06146 | -15.362 | 0.000e+00 | 0.000e+00 | VPS63 | Phenotypic enhancement | high temperature | | YIL039W | -0.9400 | 0.06146 | -15.296 | 0.000e+00 | 0.000e+00 | TED1 | Phenotypic enhancement | high temperature | | YBR121C | -0.9265 | 0.06146 | -15.076 | 0.000e+00 | 0.000e+00 | GRS1 | Phenotypic enhancement | high temperature | | YLR262C | -0.9244 | 0.06146 | -15.042 | 0.000e+00 | 0.000e+00 | YPT6 | Phenotypic enhancement | high temperature | | YGR135W | -0.9239 | 0.06146 | -15.034 | 0.000e+00 | 0.000e+00 | PRE9 | Phenotypic enhancement | high temperature | | YPL261C | -0.9199 | 0.06146 | -14.969 | 0.000e+00 | 0.000e+00 | \_ | Phenotypic enhancement | high temperature | | YMR316C-A | -0.9196 | 0.06146 | -14.965 | 0.000e+00 | 0.000e+00 | \_ | Phenotypic enhancement | high temperature | | YDR057W | -0.9171 | 0.06146 | -14.923 | 0.000e+00 | 0.000e+00 | YOS9 | Phenotypic enhancement | high temperature | | YJR108W | -0.9115 | 0.06146 | -14.832 | 0.000e+00 | 0.000e+00 | ABM1 | Phenotypic enhancement | high temperature | | YJL030W | -0.9089 | 0.06146 | -14.790 | 0.000e+00 | 0.000e+00 | MAD2 | Phenotypic enhancement | high temperature | | YCL036W | -0.9050 | 0.06146 | -14.725 | 0.000e+00 | 0.000e+00 | GFD2 | Phenotypic enhancement | high temperature | | YDR335W | -0.8968 | 0.06146 | -14.593 | 0.000e+00 | 0.000e+00 | MSN5 | Phenotypic enhancement | high temperature | | YLR418C | -0.8929 | 0.06146 | -14.529 | 0.000e+00 | 0.000e+00 | CDC73 | Phenotypic enhancement | high temperature | | YMR154C | -0.8914 | 0.06146 | -14.505 | 0.000e+00 | 0.000e+00 | RIM13 | Phenotypic enhancement | high temperature | | YER119C-A | -0.8874 | 0.06146 | -14.440 | 0.000e+00 | 0.000e+00 | \_ | Phenotypic enhancement | high temperature | | YPR097W | -0.8848 | 0.06146 | -14.398 | 0.000e+00 | 0.000e+00 | \_ | Phenotypic enhancement | high temperature | | YOR030W | -0.8741 | 0.06146 | -14.223 | 0.000e+00 | 0.000e+00 | DFG16 | Phenotypic enhancement | high temperature | | YOR001W | -0.8720 | 0.06146 | -14.190 | 0.000e+00 | 0.000e+00 | RRP6 | Phenotypic enhancement | high temperature | | YKR082W | -0.8714 | 0.06146 | -14.180 | 0.000e+00 | 0.000e+00 | NUP133 | Phenotypic enhancement | high temperature | | YBL007C | -0.8706 | 0.06146 | -14.166 | 0.000e+00 | 0.000e+00 | SLA1 | Phenotypic enhancement | high temperature | | YAL013W | -0.8697 | 0.06146 | -14.152 | 0.000e+00 | 0.000e+00 | DEP1 | Phenotypic enhancement | high temperature | | YGR122W | -0.8693 | 0.06146 | -14.146 | 0.000e+00 | 0.000e+00 | \_ | Phenotypic enhancement | high temperature | | YIL098C | -0.8672 | 0.06146 | -14.111 | 0.000e+00 | 0.000e+00 | FMC1 | Phenotypic enhancement | high temperature | | YHL027W | -0.8668 | 0.06146 | -14.104 | 0.000e+00 | 0.000e+00 | RIM101 | Phenotypic enhancement | high temperature | | YCR009C | -0.8321 | 0.06146 | -13.539 | 0.000e+00 | 0.000e+00 | RVS161 | Phenotypic enhancement | high temperature | | YDL142C | -0.8283 | 0.06146 | -13.477 | 0.000e+00 | 0.000e+00 | CRD1 | Phenotypic enhancement | high temperature | | YER178W | -0.8275 | 0.06146 | -13.466 | 0.000e+00 | 0.000e+00 | PDA1 | Phenotypic enhancement | high temperature | | YOR035C | -0.8215 | 0.06146 | -13.367 | 0.000e+00 | 0.000e+00 | SHE4 | Phenotypic enhancement | high temperature | | YGL045W | -0.8141 | 0.06146 | -13.247 | 0.000e+00 | 0.000e+00 | RIM8 | Phenotypic enhancement | high temperature | | YIL100W | -0.8137 | 0.06146 | -13.240 | 0.000e+00 | 0.000e+00 | \_ | Phenotypic enhancement | high temperature | | YOR216C | -0.8131 | 0.06146 | -13.230 | 0.000e+00 | 0.000e+00 | RUD3 | Phenotypic enhancement | high temperature | | YOR125C | -0.8108 | 0.06146 | -13.194 | 0.000e+00 | 0.000e+00 | CAT5 | Phenotypic enhancement | high temperature | | YJL214W | -0.8038 | 0.06146 | -13.080 | 0.000e+00 | 0.000e+00 | HXT8 | Phenotypic enhancement | high temperature | | YNL294C | -0.7977 | 0.06146 | -12.981 | 0.000e+00 | 0.000e+00 | RIM21 | Phenotypic enhancement | high temperature | | YFL023W | -0.7947 | 0.06146 | -12.931 | 0.000e+00 | 0.000e+00 | BUD27 | Phenotypic enhancement | high temperature | | YKR009C | -0.7918 | 0.06146 | -12.884 | 0.000e+00 | 0.000e+00 | FOX2 | Phenotypic enhancement | high temperature | | YDL006W | -0.7894 | 0.06146 | -12.846 | 0.000e+00 | 0.000e+00 | PTC1 | Phenotypic enhancement | high temperature | | YNL199C | -0.7885 | 0.06146 | -12.830 | 0.000e+00 | 0.000e+00 | GCR2 | Phenotypic enhancement | high temperature | | YMR063W | -0.7851 | 0.06146 | -12.775 | 0.000e+00 | 0.000e+00 | RIM9 | Phenotypic enhancement | high temperature | | YML062C | -0.7848 | 0.06146 | -12.770 | 0.000e+00 | 0.000e+00 | MFT1 | Phenotypic enhancement | high temperature | | YGR092W | -0.7824 | 0.06146 | -12.731 | 0.000e+00 | 0.000e+00 | DBF2 | Phenotypic enhancement | high temperature | | YOR221C | -0.7766 | 0.06146 | -12.638 | 0.000e+00 | 0.000e+00 | MCT1 | Phenotypic enhancement | high temperature | | YKL001C | -0.7757 | 0.06146 | -12.623 | 0.000e+00 | 0.000e+00 | MET14 | Phenotypic enhancement | high temperature | | YDR493W | -0.7750 | 0.06146 | -12.610 | 0.000e+00 | 0.000e+00 | AIM8 | Phenotypic enhancement | high temperature | | YLR370C | -0.7727 | 0.06146 | -12.574 | 0.000e+00 | 0.000e+00 | ARC18 | Phenotypic enhancement | high temperature | | YLL006W | -0.7725 | 0.06146 | -12.571 | 0.000e+00 | 0.000e+00 | MMM1 | Phenotypic enhancement | high temperature | | YBR082C | -0.7710 | 0.04346 | -17.743 | 0.000e+00 | 0.000e+00 | UBC4 | Phenotypic enhancement | high temperature | | YKR089C | -0.7681 | 0.06146 | -12.499 | 0.000e+00 | 0.000e+00 | TGL4 | Phenotypic enhancement | high temperature | | YPL161C | -0.7672 | 0.06146 | -12.485 | 0.000e+00 | 0.000e+00 | BEM4 | Phenotypic enhancement | high temperature | | YAR031W | -0.7595 | 0.06146 | -12.358 | 0.000e+00 | 0.000e+00 | PRM9 | Phenotypic enhancement | high temperature | | YLR044C | -0.7581 | 0.06146 | -12.336 | 0.000e+00 | 0.000e+00 | PDC1 | Phenotypic enhancement | high temperature | | YNL215W | -0.7552 | 0.06146 | -12.290 | 0.000e+00 | 0.000e+00 | IES2 | Phenotypic enhancement | high temperature | | YGL007W | -0.7529 | 0.06146 | -12.251 | 0.000e+00 | 0.000e+00 | BRP1 | Phenotypic enhancement | high temperature | | YLR242C | -0.7522 | 0.06146 | -12.240 | 0.000e+00 | 0.000e+00 | ARV1 | Phenotypic enhancement | high temperature | | YKR020W | -0.7502 | 0.06146 | -12.207 | 0.000e+00 | 0.000e+00 | VPS51 | Phenotypic enhancement | high temperature | | YBR077C | -0.7300 | 0.06146 | -11.878 | 0.000e+00 | 0.000e+00 | SLM4 | Phenotypic enhancement | high temperature | | YNL198C | -0.7286 | 0.06146 | -11.856 | 0.000e+00 | 0.000e+00 | \_ | Phenotypic enhancement | high temperature | | YGL046W | -0.7282 | 0.06146 | -11.849 | 0.000e+00 | 0.000e+00 |  | Phenotypic enhancement | high temperature | | YLR338W | -0.7274 | 0.06146 | -11.837 | 0.000e+00 | 0.000e+00 | OPI9 | Phenotypic enhancement | high temperature | | YGL219C | -0.7256 | 0.06146 | -11.806 | 0.000e+00 | 0.000e+00 | MDM34 | Phenotypic enhancement | high temperature | | YMR060C | -0.7231 | 0.06146 | -11.766 | 0.000e+00 | 0.000e+00 | SAM37 | Phenotypic enhancement | high temperature | | YKL069W | -0.7224 | 0.06146 | -11.756 | 0.000e+00 | 0.000e+00 | \_ | Phenotypic enhancement | high temperature | | YBR026C | -0.7217 | 0.06146 | -11.743 | 0.000e+00 | 0.000e+00 | ETR1 | Phenotypic enhancement | high temperature | | YIL128W | -0.7183 | 0.06146 | -11.688 | 0.000e+00 | 0.000e+00 | MET18 | Phenotypic enhancement | high temperature | | YHR167W | -0.7099 | 0.06146 | -11.552 | 0.000e+00 | 0.000e+00 | THP2 | Phenotypic enhancement | high temperature | | YER155C | -0.7093 | 0.06146 | -11.542 | 0.000e+00 | 0.000e+00 | BEM2 | Phenotypic enhancement | high temperature | | YKL113C | -0.7077 | 0.06146 | -11.515 | 0.000e+00 | 0.000e+00 | RAD27 | Phenotypic enhancement | high temperature | | YKL048C | -0.7036 | 0.06146 | -11.449 | 0.000e+00 | 0.000e+00 | ELM1 | Phenotypic enhancement | high temperature | | YGL162W | -0.7019 | 0.06146 | -11.421 | 0.000e+00 | 0.000e+00 | SUT1 | Phenotypic enhancement | high temperature | | YAL002W | -0.7002 | 0.06146 | -11.393 | 0.000e+00 | 0.000e+00 | VPS8 | Phenotypic enhancement | high temperature | | YBR221C | -0.7002 | 0.06146 | -11.393 | 0.000e+00 | 0.000e+00 | PDB1 | Phenotypic enhancement | high temperature | | YPR155C | -0.6988 | 0.06146 | -11.371 | 0.000e+00 | 0.000e+00 | NCA2 | Phenotypic enhancement | high temperature | | YOR089C | -0.6953 | 0.06146 | -11.314 | 0.000e+00 | 0.000e+00 | VPS21 | Phenotypic enhancement | high temperature | | YHL031C | -0.6898 | 0.06146 | -11.225 | 0.000e+00 | 0.000e+00 | GOS1 | Phenotypic enhancement | high temperature | | YBR283C | -0.6870 | 0.06146 | -11.179 | 0.000e+00 | 0.000e+00 | SSH1 | Phenotypic enhancement | high temperature | | YKR065C | -0.6840 | 0.06146 | -11.130 | 0.000e+00 | 0.000e+00 | PAM17 | Phenotypic enhancement | high temperature | | YHR067W | -0.6807 | 0.06146 | -11.076 | 0.000e+00 | 0.000e+00 | HTD2 | Phenotypic enhancement | high temperature | | YBR209W | -0.6766 | 0.06146 | -11.010 | 0.000e+00 | 0.000e+00 | \_ | Phenotypic enhancement | high temperature | | YIL110W | -0.6656 | 0.06146 | -10.831 | 0.000e+00 | 0.000e+00 | MNI1 | Phenotypic enhancement | high temperature | | YDR363W-A | -0.6636 | 0.06146 | -10.798 | 0.000e+00 | 0.000e+00 | SEM1 | Phenotypic enhancement | high temperature | | YDL095W | -0.6630 | 0.06146 | -10.788 | 0.000e+00 | 0.000e+00 | PMT1 | Phenotypic enhancement | high temperature | | YMR036C | -0.6608 | 0.06146 | -10.752 | 0.000e+00 | 0.000e+00 | MIH1 | Phenotypic enhancement | high temperature | | YBR215W | -0.6601 | 0.06146 | -10.741 | 0.000e+00 | 0.000e+00 | HPC2 | Phenotypic enhancement | high temperature | | YLR268W | -0.6570 | 0.06146 | -10.691 | 0.000e+00 | 0.000e+00 | SEC22 | Phenotypic enhancement | high temperature | | YCR008W | -0.6560 | 0.06146 | -10.675 | 0.000e+00 | 0.000e+00 | SAT4 | Phenotypic enhancement | high temperature | | YNL171C | -0.6551 | 0.06146 | -10.660 | 0.000e+00 | 0.000e+00 | \_ | Phenotypic enhancement | high temperature | | YER061C | -0.6508 | 0.06146 | -10.590 | 0.000e+00 | 0.000e+00 | CEM1 | Phenotypic enhancement | high temperature | | YAR044W | -0.6496 | 0.06146 | -10.570 | 0.000e+00 | 0.000e+00 |  | Phenotypic enhancement | high temperature | | YGR054W | -0.6396 | 0.06146 | -10.407 | 0.000e+00 | 0.000e+00 | \_ | Phenotypic enhancement | high temperature | | YMR119W | -0.6375 | 0.04346 | -14.670 | 0.000e+00 | 0.000e+00 | ASI1 | Phenotypic enhancement | high temperature | | YOR026W | -0.6366 | 0.06146 | -10.359 | 0.000e+00 | 0.000e+00 | BUB3 | Phenotypic enhancement | high temperature | | YCR044C | -0.6335 | 0.06146 | -10.308 | 0.000e+00 | 0.000e+00 | PER1 | Phenotypic enhancement | high temperature | | YDL109C | -0.6320 | 0.02323 | -27.211 | 0.000e+00 | 0.000e+00 | \_ | Phenotypic enhancement | high temperature | | YMR224C | -0.6275 | 0.02323 | -27.014 | 0.000e+00 | 0.000e+00 | MRE11 | Phenotypic enhancement | high temperature | | YNL169C | -0.6239 | 0.06146 | -10.153 | 0.000e+00 | 0.000e+00 | PSD1 | Phenotypic enhancement | high temperature | | YGR188C | -0.6236 | 0.06146 | -10.147 | 0.000e+00 | 0.000e+00 | BUB1 | Phenotypic enhancement | high temperature | | YBR071W | -0.6205 | 0.06146 | -10.096 | 0.000e+00 | 0.000e+00 | \_ | Phenotypic enhancement | high temperature | | YBR126C | -0.6199 | 0.06146 | -10.086 | 0.000e+00 | 0.000e+00 | TPS1 | Phenotypic enhancement | high temperature | | YER135C | -0.6186 | 0.06146 | -10.066 | 0.000e+00 | 0.000e+00 | \_ | Phenotypic enhancement | high temperature | | YML028W | -0.6174 | 0.02323 | -26.579 | 0.000e+00 | 0.000e+00 | TSA1 | Phenotypic enhancement | high temperature | | YPL213W | -0.6084 | 0.06146 | -9.900 | 0.000e+00 | 0.000e+00 | LEA1 | Phenotypic enhancement | high temperature | | YKL055C | -0.6068 | 0.06146 | -9.873 | 0.000e+00 | 0.000e+00 | OAR1 | Phenotypic enhancement | high temperature | | YDR110W | -0.6063 | 0.06146 | -9.867 | 0.000e+00 | 0.000e+00 | FOB1 | Phenotypic enhancement | high temperature | | YDL136W | -0.6035 | 0.06146 | -9.820 | 0.000e+00 | 0.000e+00 | RPL35B | Phenotypic enhancement | high temperature | | YBL008W | -0.6021 | 0.06146 | -9.797 | 0.000e+00 | 0.000e+00 | HIR1 | Phenotypic enhancement | high temperature | | YCL022C | -0.5918 | 0.06146 | -9.630 | 0.000e+00 | 0.000e+00 | \_ | Phenotypic enhancement | high temperature | | YIL132C | -0.5829 | 0.06146 | -9.486 | 0.000e+00 | 0.000e+00 | CSM2 | Phenotypic enhancement | high temperature | | YMR119W-A | -0.5759 | 0.06146 | -9.372 | 0.000e+00 | 0.000e+00 | \_ | Phenotypic enhancement | high temperature | | YHR081W | -0.5573 | 0.06146 | -9.069 | 0.000e+00 | 0.000e+00 | LRP1 | Phenotypic enhancement | high temperature | | YPR024W | -0.5556 | 0.06146 | -9.040 | 0.000e+00 | 0.000e+00 | YME1 | Phenotypic enhancement | high temperature | | YBR015C | -0.5515 | 0.06146 | -8.975 | 0.000e+00 | 0.000e+00 | MNN2 | Phenotypic enhancement | high temperature | | YDL232W | -0.5491 | 0.06146 | -8.934 | 0.000e+00 | 0.000e+00 | OST4 | Phenotypic enhancement | high temperature | | YNR074C | -0.5487 | 0.06146 | -8.929 | 0.000e+00 | 0.000e+00 | AIF1 | Phenotypic enhancement | high temperature | | YJL213W | -0.5467 | 0.06146 | -8.896 | 0.000e+00 | 0.000e+00 | \_ | Phenotypic enhancement | high temperature | | YER139C | -0.5454 | 0.06146 | -8.874 | 0.000e+00 | 0.000e+00 | RTR1 | Phenotypic enhancement | high temperature | | YDL011C | -0.5400 | 0.06146 | -8.787 | 0.000e+00 | 0.000e+00 | \_ | Phenotypic enhancement | high temperature | | YNL071W | -0.5376 | 0.06146 | -8.748 | 0.000e+00 | 0.000e+00 | LAT1 | Phenotypic enhancement | high temperature | | YHR163W | -0.5368 | 0.06146 | -8.735 | 0.000e+00 | 0.000e+00 | SOL3 | Phenotypic enhancement | high temperature | | YPR173C | -0.5361 | 0.06146 | -8.724 | 0.000e+00 | 0.000e+00 | VPS4 | Phenotypic enhancement | high temperature | | YDR512C | -0.5314 | 0.06146 | -8.648 | 0.000e+00 | 0.000e+00 | EMI1 | Phenotypic enhancement | high temperature | | YOR196C | -0.5268 | 0.06146 | -8.573 | 0.000e+00 | 0.000e+00 | LIP5 | Phenotypic enhancement | high temperature | | YOR120W | -0.5215 | 0.06146 | -8.486 | 0.000e+00 | 0.000e+00 | GCY1 | Phenotypic enhancement | high temperature | | YLR110C | -0.5197 | 0.06146 | -8.456 | 0.000e+00 | 0.000e+00 | CCW12 | Phenotypic enhancement | high temperature | | YLR337C | -0.5191 | 0.06146 | -8.447 | 0.000e+00 | 0.000e+00 | VRP1 | Phenotypic enhancement | high temperature | | YER140W | -0.5189 | 0.06146 | -8.444 | 0.000e+00 | 0.000e+00 | \_ | Phenotypic enhancement | high temperature | | YNL064C | -0.5169 | 0.06146 | -8.410 | 0.000e+00 | 0.000e+00 | YDJ1 | Phenotypic enhancement | high temperature | | YIR005W | -0.5101 | 0.06146 | -8.301 | 0.000e+00 | 0.000e+00 | IST3 | Phenotypic enhancement | high temperature | | YNR020C | -0.5089 | 0.06146 | -8.281 | 2.220e-16 | 4.599e-15 | ATP23 | Phenotypic enhancement | high temperature | | YHR033W | -0.5048 | 0.06146 | -8.214 | 2.220e-16 | 4.599e-15 | \_ | Phenotypic enhancement | high temperature | | YLR119W | -0.5047 | 0.06146 | -8.212 | 2.220e-16 | 4.599e-15 | SRN2 | Phenotypic enhancement | high temperature | | YDL100C | -0.5035 | 0.06146 | -8.193 | 2.220e-16 | 4.599e-15 | GET3 | Phenotypic enhancement | high temperature | | YDR043C | -0.5018 | 0.06146 | -8.165 | 2.220e-16 | 4.599e-15 | NRG1 | Phenotypic enhancement | high temperature | | YGL174W | -0.5002 | 0.06146 | -8.139 | 4.441e-16 | 9.154e-15 | BUD13 | Phenotypic enhancement | high temperature | | YNL227C | 0.5115 | 0.06146 | 8.323 | 0.000e+00 | 0.000e+00 | JJJ1 | Phenotypic suppression | high temperature | | YJL121C | 0.5196 | 0.06146 | 8.456 | 0.000e+00 | 0.000e+00 | RPE1 | Phenotypic suppression | high temperature | | YNL226W | 0.5651 | 0.06146 | 9.195 | 0.000e+00 | 0.000e+00 | \_ | Phenotypic suppression | high temperature | | YJL120W | 0.5658 | 0.06146 | 9.206 | 0.000e+00 | 0.000e+00 | \_ | Phenotypic suppression | high temperature | | YPR057W | 0.5938 | 0.06146 | 9.662 | 0.000e+00 | 0.000e+00 | BRR1 | Phenotypic suppression | high temperature | |
