# Supplementary material for: Quantitative Fitness Analysis Shows That NMD Proteins and Many Other Protein Complexes Suppress or Enhance Distinct Telomere Cap Defects
Source: PLoS Genet. 2011 Apr 7;7(4):e1001362. doi: 10.1371/journal.pgen.1001362 (PMC3072368; doi:10.1371/journal.pgen.1001362)
Supplement: Text S1 — Supplemental experimental procedures, strains and strain collections list, supplemental references. (0.12 MB PDF) [file pgen.1001362.s014.pdf]

Supplemental Data for Addinall *et al*: Quantitative Fitness Analysis shows that NMD proteins and many other protein complexes suppress or enhance distinct telomere cap defects in budding yeast

Supplementary Material Contents

Note that further information, including raw data files can be downloaded from our supporting information data files website:
http://research.ncl.ac.uk/colonyzer/AddinallQFA/

Figure S1. Fitness plots for *cdc13-1* vs. *ura3Δ* strains at 20°C and 27°C and for *ura3Δ* strains at 20 °C vs. 37°C

Figure S2. W303 Spot tests

Figure S3. Genetic interaction strength (GIS) analyses of ribosomal, telomere length maintenance, and *cdc13-1* suppressor genes

Figure S4. Effects of Nonsense Mediated Decay genes on telomere capping mutants

Table S1. List of suppressors and enhancers of *yku70Δ* defect at 23°C

Table S2. List of suppressors and enhancers of *yku70Δ* defect at 30°C

Table S3. List of suppressors and enhancers of *yku70Δ* defect at 37°C

Table S4. List of suppressors and enhancers of *yku70Δ* defect at 37.5°C

Table S5. List of suppressors and enhancers of *cdc13-1* defect at 20°C

Table S6. List of suppressors and enhancers of *cdc13-1* defect at 27°C

Table S7. Complete ROD output (labelled cell-density measurements for all experiments)

Table S8. Logistic data file (estimated logistic model parameters for all experiments)

Table S9. List of suppressors and enhancers of temperature-induced fitness defect at 37°C

Supplemental Experimental Procedures

Strains and strain collections list

Supplemental References

Supplemental Experimental Procedures

Strains, strain collections, oligonucleotide primers and plasmids.

Strain	Genetic Background	Genotype	Alternative Name / Construction	Reference / Source
Y2454	S288C	MAT*α ura3 leu2 his3 lys2 mfa::MFA1pr-HIS3 can1*	BY4742 *mfa::MFA1pr-HIS3 can1*	Charles Boone
DLY640	W303	MATa *ade2-1 trp1-1 can1-100 leu2-3,112 his3-11,15 ura3 GAL+ psi+ ssd1-d2 RAD5*		Rodney Rothstein
DLY659	W303	MATa *rad24::TRP1 ade2-1 trp1-1 can1-100 leu2-3,112 his3-11,15 ura3 GAL+ psi+ ssd1-d2 RAD5*	DLY652 x DLY640	
DLY1108	W303	MATa *cdc13-1 int ade2-1 trp1-1 can1-100 leu2-3,112 his3-11,15 ura3 GAL+ psi+ ssd1-d2 RAD5*		(Zubko et al., 2004)
DLY1258	W303	MAT*α cdc13-1 int rad24::TRP1 ade2-1 trp1-1 can1-100 leu2-3,112 his3-11,15 ura3 GAL+ psi+ ssd1-d2 RAD5*	641 x 685	
DLY1273	W303	MAT*α exo1::LEU2 ade2-1 trp1-1 can1-100 leu2-3,112 his3-11,15 ura3 GAL+ psi+ ssd1-d2 RAD5*	641 x 685	
DLY1284	W303	MATa *yku70::HIS3 rad24::TRP1ade2-1 trp1-1 can1-100 leu2-3,112 his3-11,15 ura3 GAL+ psi+ ssd1-d2 RAD5*	DLY978 x DLY1258	(Maringele et al., 2002)
DLY1296	W303	MATa *cdc13-1 int exo1::LEU2 ade2-1 trp1-1 can1-100 leu2-3,112 his3-11,15 ura3 GAL+ psi+ ssd1-d2 RAD5*	DLY1272 x DLY1230	
DLY1409	W303	MAT*α exo1::LEU2 yku70::HIS3 ade2-1 trp1-1 can1-100 leu2-3,112 his3-11,15 ura3 GAL+ psi+ ssd1-d2 RAD5*	DLY1273 x DLY1364	(Maringele et al., 2002)
DLY1412	W303	MATa *yku70::HIS3 ade2-1 trp1-1 can1-100 leu2-3,112 his3-11,15 ura3 GAL+ psi+ ssd1-d2 RAD5*	DLY1273 x DLY1364	(Maringele et al., 2002)
DLY1622	S288C	Y2454 *cdc13-1* int		(Downey et al., 2006)
DLY2198	S288C	DLY1622 *cdc13-1* int::URA3		(Downey et al., 2006)
DLY2413	W303	MATa *yku70::TRP1 mlp1::KANMX5 PDS1-HA-URA3 ade2-1 trp1-1 can1-100 leu2-3,112 his3-11,15 ura3 GAL+ psi+ ssd1-d2 rad5-535*	JCY245-4A	Joseph Campbell
DLY2415	W303	MATa *yku70::TRP1 mlp2::HIS5 PDS1-HA-URA3 ade2-1 trp1-1 can1-100 leu2-3,112 his3-11,15 ura3 GAL+ psi+ ssd1-d2 rad5-535*	JCY245-28D	
DLY2764	W303	MATa *ebs1::URA3 ade2-1 trp1-1 can1-100 leu2-3,112 his3-11,15 ura3 GAL+ psi+ ssd1-d2 RAD5*	DLY2824 transformed with pDL1079	This work
DLY2787	W303	MATα *yku70Δ::LEU2 ade2-1 trp1-1 can1-100 leu2-3,112 his3-11,15 ura3 GAL+ psi+ ssd1-d2 RAD5*	DLY1264 x<br>DLY2385	This work
DLY2801	S288C	MAT*a ura3 leu2 his3 met15 yku70Δ::KANMX*	*BY4741 yku70Δ::KANMX*	Ben Chang
DLY2804	S288C	MAT*α ura3 leu2 his3 lys2 isw2::KANMX*	*BY4742 isw2::KANMX*	Ben Chang
DLY2824	W303	MAT*a ebs1::KANMX ade2-1 trp1-1 can1-100 leu2-3,112 his3-11,15 ura3 GAL+ psi+ ssd1-d2 RAD5*	DLY1264 x<br>DLY2400	This work

DLY2889	W303	MAT*a yku70*Δ*::LEU2 ebs1::KANMX ade2-1 trp1-1 can1-100 leu2-3,112 his3-11,15 ura3 GAL+ psi+ ssd1-d2 RAD5*	DLY2787 x DLY2824	This work
DLY2890	W303	MAT*a yku70::LEu2 ebs1::KANMX ade2-1 trp1-1 can1-100 leu2-3,112 his3-11,15 ura3 GAL+ psi+ ssd1-d2 RAD5*	DLY2824 x DLY2787	This work
DLY3001	W303	MAT*a ade2-1 trp1-1 can1-100 leu2-3,112 his3-11,15 ura3 GAL+ psi+ ssd1-d2 RAD5*		Rodney Rothstein
DLY3336	S288C	*MATa yku70*Δ*::URA3*	DLY2801 marker swap using pDL1079	This work.
DLY3337	S288C	MAT*α isw2::LEU2*	DLY2801 marker swap using pDL1077	This work.
DLY3340	S288C	MATa *ebs1::KANMX*	from SGAv2 (see below)	Charles Boone
DLY3341	S288C	MAT*α yku70*Δ*::URA3*	DLY3336 x DLY3337	This work.
DLY3342	S288C	MATa *ebs1::KANMX*	DLY3340 failed marker swap using pDL1077	This work.
DLY3362	S288C	MATa *yku70*Δ*::URA3 ebs1::KANMX*	DLY3341 x DLY3342	This work.
DLY3412	S288C	MAT*α can1Δ::MFA1pr-HIS3 lyp1Δ his3Δ1 leu2Δ0 ura3Δ0 met15Δ0 LYS2+*	Y5563	Charles Boone
DLY3541	S288C	MAT*α yku70*Δ*::URA3 can1Δ::MFA1pr-HIS3 lyp1Δ his3Δ1 leu2Δ0 ura3Δ0 met15Δ0 LYS2+*	DLY3362 x DLY3412	This work.
DLY3622	W303	MAT*α oca1::KANMX ade2-1 trp1-1 can1-100 leu2-3,112 his3-11,15 ura3 GAL+ psi+ ssd1-d2 RAD5*	DDY145	This work
DLY3624	W303	MAT*a cdc13-1 int oca1::KANMX ade2-1 trp1-1 can1-100 leu2-3,112 his3-11,15 ura3 GAL+ psi+ ssd1-d2 RAD5*	DDY145	This work
DLY3653	W303	MAT*α oca2::KANMX ade2-1 trp1-1 can1-100 leu2-3,112 his3-11,15 ura3 GAL+ psi+ ssd1-d2 RAD5*	DDY145	This work
DLY3655	W303	MAT*α cdc13-1 int oca2::KANMX ade2-1 trp1-1 can1-100 leu2-3,112 his3-11,15 ura3 GAL+ psi+ ssd1-d2 RAD5*	DDY145	This work
DLY4228	S288C	MATα *ura3*::*NATMX can1::MFA1pr-HIS3 lyp1*Δ *cyh2,* his3Δ, *leu2*Δ*0 met15*Δ*0 LYS2+*	Y7221	Charles Boone
DLY4290	W303	MATa *yku70::LEU2 oca1::KANMX ade2-1 trp1-1 can1-100 leu2-3,112 his3-11,15 ura3 GAL+ psi+ ssd1-d2 RAD5*	DLY3622 x DLY3223	This work
DLY4296	W303	MAT*α yku70::LEU2 oca2::KANMX ade2-1 trp1-1 can1-100 leu2-3,112 his3-11,15 ura3 GAL+ psi+ ssd1-d2 RAD5*	DLY3653 x DLY3223	This work
DLY4309	W303	MATa *yku70::LEU2 ade2-1 trp1-1 can1-100 leu2-3,112 his3-11,15 ura3 GAL+ psi+ ssd1-d2 RAD5*	DLY3882 x DLY3223	This work
DLY4557	W303	MAT*a ade2-1 trp1-1 can1-100 leu2-3,112 his3-11,15 ura3 GAL+ psi+ ssd1-d2 RAD5 cdc13-1-int*		This work
DLY4576	W303	MAT*a cdc13-1 ebs1::KANMX ade2-1 trp1-1 can1-100 leu2-3,112 his3-11,15 ura3 GAL+ psi+ ssd1-d2 RAD5*	DLY2826 x DLY4202	This work
DLY4577	W303	MAT*a ebs1::KANMX cdc13-1-int ade2-1 trp1-1 can1-100 leu2-3,112 his3-11,15 ura3 GAL+ psi+ ssd1-d2 RAD5*	DLY4577 x DLY4202	This work

DLY4624	W303	MAT*a cdc13-1 nmd2::HIS3 ade2-1 trp1-1 can1-100 leu2-3,112 his3-11,15 ura3 GAL+ psi+ ssd1-d2 RAD5*	DLY2826 x DLY4528	This work
DLY4625	W303	MATa *nmd2::HIS3 cdc13-1-int ade2-1 trp1-1 can1-100 leu2-3,112 his3-11,15 ura3 GAL+ psi+ ssd1-d2 RAD5*	DLY4625 x DLY4528	This work
DLY4626	W303	MAT*α cdc13-1 ebs1::KANMX nmd2::HIS3 ade2-1 trp1-1 can1-100 leu2-3,112 his3-11,15 ura3 GAL+ psi+ ssd1-d2 RAD5*	DLY2826 x DLY4528	This work
DLY4763	W303	MAT*a ade2-1 trp1-1 can1-100 leu2-3,112 his3-11,15 ura3 GAL+ psi+ ssd1-d2 RAD5*	DLY4528 x DLY4674	This work
DLY4764	W303	MATa *ebs1::KanMX ade2-1 trp1-1 can1-100 leu2-3,112 his3-11,15 ura3 GAL+ psi+ ssd1-d2 RAD5*	DLY4528 x DLY4674	This work
DLY4765	W303	MAT*a nmd2::HIS ade2-1 trp1-1 can1-100 leu2-3,112 his3-11,15 ura3 GAL+ psi+ ssd1-d2 RAD5*	DLY4528 x DLY4674	This work
DLY4766	W303	MAT*α nmd2::HIS ade2-1 trp1-1 can1-100 leu2-3,112 his3-11,15 ura3 GAL+ psi+ ssd1-d2 RAD5*	DLY4528 x DLY4674	This work
DLY4780	W303	MAT*a ade2-1 trp1-1 can1-100 leu2-3,112 his3-11,15 ura3 GAL+ psi+ ssd1-d2 RAD5*	DLY4528 x DLY4674	This work
DLY4781	W303	MAT*ç ebs1::KanMX ade2-1 trp1-1 can1-100 leu2-3,112 his3-11,15 ura3 GAL+ psi+ ssd1-d2 RAD5*	DLY4528 x DLY4674	This work
DLY4782	W303	MATa *ebs1::KanMX ade2-1 trp1-1 can1-100 leu2-3,112 his3-11,15 ura3 GAL+ psi+ ssd1-d2 RAD5*	DLY4528 x DLY4674	This work
DLY4783	W303	MATa *nmd2::HIS ade2-1 trp1-1 can1-100 leu2-3,112 his3-11,15 ura3 GAL+ psi+ ssd1-d2 RAD5*	DLY4528 x DLY4674	This work
DLY4784	W303	MATa *nmd2::HIS ade2-1 trp1-1 can1-100 leu2-3,112 his3-11,15 ura3 GAL+ psi+ ssd1-d2 RAD5*	DLY4528 x DLY4674	This work
DLY5007	W303	MAT*a yku70::LEU2 nmd2::HIS3 ade2-1 trp1-1 can1-100 leu2-3,112 his3-11,15 ura3 GAL+ psi+ ssd1-d2 RAD5*	DLY2787 x DLY4765	This work
DLY5008	W303	MATa *yku70::LEU2 nmd2::HIS3 ade2-1 trp1-1 can1-100 leu2-3,112 his3-11,15 ura3 GAL+ psi+ ssd1-d2 RAD5*	DLY2787 x DLY4765	This work
DLY5107	W303	MAT*α nmd2::HIS3 cdc13-1-int ade2-1 trp1-1 can1-100 leu2-3,112 his3-11,15 ura3 GAL+ psi+ ssd1-d2 RAD5*	DLY4557 x DLY4766	This work
DLY5238	W303	MAT*α ebs1::KANMX nmd2::HIS3 cdc13-1-int ade2-1 trp1-1 can1-100 leu2-3,112 his3-11,15 ura3 GAL+ psi+ ssd1-d2 RAD5*	DLY1696 x DLY4627	This work
DLY5239	W303	MAT*α ebs1::KANMX nmd2::HIS3 cdc13-1-int ade2-1 trp1-1 can1-100 leu2-3,112 his3-11,15 ura3 GAL+ psi+ ssd1-d2 RAD5*	DLY1696 x DLY4627	This work
DLY5242	W303	MAT*α yku70Δ::LEU2 ebs1::KANMX nmd2::HIS3 ade2-1 trp1-1 can1-100 leu2-3,112 his3-11,15 ura3 GAL+ psi+ ssd1-d2 RAD5*	DLY2889 x DLY4766	This work
DLY5251	W303	MATa *yku70::LEU2 nmd2::HIS3 ebs1::KANMX ade2-1 trp1-1 can1-100 leu2-3,112 his3-11,15 ura3 GAL+ psi+ ssd1-d2 RAD5*	DLY2889 x DLY4766	This work
DLY5688	S288C	MATα *LEU2::cdc13-1 int::HPHMX lyp1Δ can1::MFA1pr-HIS3 ura3 leu2 his3 LYS2+*	DLY5382 x DLY3704	This work.
DLY5757	W303	*STN1-C-MYC::TRP1 yku70Δ::LEU2*	DLY2704 x DLY5251	This work

DLY5758	W303	MATa *STN1-C-MYC::TRP1 ebs1::KANMX*	DLY5756 x DLY5757	This work
DLY5759	W303	MATa *STN1-C-MYC::TRP1 nmd2::HIS3*	DLY5756 x DLY5757	This work
DLY5760	W303	MATα *STN1-C-MYC::TRP1 cdc13-1*	DLY5756 x DLY5757	This work
DLY5761	W303	MATa *STN1-C-MYC::TRP1*	DLY5756 x DLY5757	This work
DLY5763	W303	MATa *STN1-C-MYC::TRP1 ebs1::KANMX cdc13-1*	DLY5756 x DLY5757	This work
DLY5764	W303	MATα *STN1-C-MYC::TRP1 ebs1::KANMX yku70Δ::LEU2*	DLY5756 x DLY5757	This work
DLY5765	W303	MATa *STN1-C-MYC::TRP1 nmd2::HIS3 yku70Δ::LEU2*	DLY5756 x DLY5757	This work
DLY5766	W303	MATα *STN1-C-MYC::TRP1 nmd2::HIS3 cdc13-1*	DLY5756 x DLY5757	This work
DLY6332	W303	MATα *pph3::HPHMX ade2-1 trp1-1 can1-100 leu2-3,112 his3-11,15 ura3 GAL+ psi+ ssd1-d2 RAD5*	DDY461	This work
DLY6396	W303	MATa *cdc13-1 int pph3::HPHMX ade2-1 trp1-1 can1-100 leu2-3,112 his3-11,15 ura3 GAL+ psi+ ssd1-d2 RAD5*	DLY6332 x DLY5085	This work
DLY6614	W303	MATa *cdc13-1 int elp6::KANMX ade2-1 trp1-1 can1-100 leu2-3,112 his3-11,15 ura3 GAL+ psi+ ssd1-d2 RAD5*	DLY6613 x DLY1255	This work
DLY6620	W303	MATa *elp6::KANMX ade2-1 trp1-1 can1-100 leu2-3,112 his3-11,15 ura3 GAL+ psi+ ssd1-d2 RAD5*	DLY6613 x DLY1255	This work
DLY6628	W303	MATα *yku70::LEU2 elp6::KANMX ade2-1 trp1-1 can1-100 leu2-3,112 his3-11,15 ura3 GAL+ psi+ ssd1-d2 RAD5*	DLY6613 x DLY1215	This work
DLY6656	W303	MATa *nam7::KANMX ade2-1 trp1-1 can1-100 leu2-3,112 his3-11,15 ura3 GAL+ psi+ ssd1-d2 RAD5*	DLY1696 x DLY4627	This work
DLY6692	W303	MATα *rrd1::NATMX ade2-1 trp1-1 can1-100 leu2-3,112 his3-11,15 ura3 GAL+ psi+ ssd1-d2 RAD5*	DDY502	This work
DLY6694	W303	MATa *cdc13-1 int rrd1::NATMX ade2-1 trp1-1 can1-100 leu2-3,112 his3-11,15 ura3 GAL+ psi+ ssd1-d2 RAD5*	DDY502	This work
DLY6763	W303	MATa *yku70::HIS3 pph3::HPHMX ade2-1 trp1-1 can1-100 leu2-3,112 his3-11,15 ura3 GAL+ psi+ ssd1-d2 RAD5*	DLY1408 x DLY6332	This work
DLY6776	W303	MATa *yku70::HIS3 rrd1::NATMX ade2-1 trp1-1 can1-100 leu2-3,112 his3-11,15 ura3 GAL+ psi+ ssd1-d2 RAD5*	DLY6696 x DLY6761	This work
DLY6808	W303	MATα *yku70::LEU2 nam7::KANMX ade2-1 trp1-1 can1-100 leu2-3,112 his3-11,15 ura3 GAL+ psi+ ssd1-d2 RAD5*	DLY6656 x DLY2984	This work
DLY6810	W303	MATα *cdc13-1 int nam7::KANMX ade2-1 trp1-1 can1-100 leu2-3,112 his3-11,15 ura3 GAL+ psi+ ssd1-d2 RAD5*	DLY6656 x DLY1256	This work
DLY6811	W303	MATa *upf3::KANMX ade2-1 trp1-1 can1-100 leu2-3,112 his3-11,15 ura3 GAL+ psi+ ssd1-d2 RAD5*	DDY227	This work

DLY6812	W303	MATα *yku70::LEU2 upf3::KANMX ade2-1 trp1-1 can1-100 leu2-3,112 his3-11,15 ura3 GAL+ psi+ ssd1-d2 RAD5*	DLY6811 x DLY2984	This work
DLY6814	W303	MATa *cdc13-1 int upf3::KANMX ade2-1 trp1-1 can1-100 leu2-3,112 his3-11,15 ura3 GAL+ psi+ ssd1-d2 RAD5*	DLY6811 x DLY1256	This work
DLY6860	W303	MATα *mlp1::KANMX ade2-1 trp1-1 can1-100 leu2-3,112 his3-11,15 ura3 GAL+ psi+ ssd1-d2 RAD5*	DDY227	This work
DLY6862	W303	MATa *cdc13-1 int mlp1::KANMX ade2-1 trp1-1 can1-100 leu2-3,112 his3-11,15 ura3 GAL+ psi+ ssd1-d2 RAD5*	DLY2412 x DLY1256	This work
DLY6865	W303	MATα *cdc13-1 int mak31::KANMX ade2-1 trp1-1 can1-100 leu2-3,112 his3-11,15 ura3 GAL+ psi+ ssd1-d2 RAD5*	DDY227	This work
DLY6867	W303	MATa nam7::KANMX nmd2::HIS3 *cdc13-1 int ade2-1 trp1-1 can1-100 leu2-3,112 his3-11,15 ura3 GAL+ psi+ ssd1-d2 RAD5*	DLY6810 x DLY6866	This work
DLY6868	W303	MATα nam7::KANMX nmd2::HIS3 *cdc13-1 int ade2-1 trp1-1 can1-100 leu2-3,112 his3-11,15 ura3 GAL+ psi+ ssd1-d2 RAD5*	DLY6810 x DLY6866	This work
DLY6927	W303	MATa *mlp2::HIS5 ade2-1 trp1-1 can1-100 leu2-3,112 his3-11,15 ura3 GAL+ psi+ ssd1-d2 RAD5*	DLY2414 x DLY4685	This work
DLY6928	W303	MATα *cdc13-1 mlp2::HIS5 ade2-1 trp1-1 can1-100 leu2-3,112 his3-11,15 ura3 GAL+ psi+ ssd1-d2 RAD5*	DLY2414 x DLY4685	This work
DLY6951	W303	MATα *mak31::KANMX ade2-1 trp1-1 can1-100 leu2-3,112 his3-11,15 ura3 GAL+ psi+ ssd1-d2 RAD5*	DDY227	This work
DLY6968	W303	MATα yku70::HIS3 *mak31::KANMX ade2-1 trp1-1 can1-100 leu2-3,112 his3-11,15 ura3 GAL+ psi+ ssd1-d2 RAD5*	DLY6951 x DLY1412	This work
DLY6963	W303	MATa *dph1::KANMX ade2-1 trp1-1 can1-100 leu2-3,112 his3-11,15 ura3 GAL+ psi+ ssd1-d2 RAD5*	DDY227	This work
DLY6967	W303	MATa *cdc13-1 int dph1::KANMX ade2-1 trp1-1 can1-100 leu2-3,112 his3-11,15 ura3 GAL+ psi+ ssd1-d2 RAD5*	DDY227	This work
DLY6971	W303	MATa *yku70::HIS3 dph1::KANMX ade2-1 trp1-1 can1-100 leu2-3,112 his3-11,15 ura3 GAL+ psi+ ssd1-d2 RAD5*	DLY6963 x DLY1366	This work
DLY6974	W303	MATα *nmd2::HIS3 nam7::KANMX yku70::LEU2 ade2-1 trp1-1 can1-100 leu2-3,112 his3-11,15 ura3 GAL+ psi+ ssd1-d2 RAD5*	DLY5007 x DLY6656	This work
DLY6975	W303	MATa *nmd2::HIS3 nam7::KANMX yku70::LEU2 ade2-1 trp1-1 can1-100 leu2-3,112 his3-11,15 ura3 GAL+ psi+ ssd1-d2 RAD5*	DLY5007 x DLY6656	This work
DLY6976	W303	MATa *nmd2::HIS3 nam7::KANMX ade2-1 trp1-1 can1-100 leu2-3,112 his3-11,15 ura3 GAL+ psi+ ssd1-d2 RAD5*	DLY5007 x DLY6656	This work
DLY6977	W303	MATa *Est2-13Myc::HIS3 ade2-1 trp1-1 can1-100 leu2-3,112 his3-11,15 ura3 GAL+ psi+ ssd1-d2 RAD5*	DLY6737 x DLY6741	This work
DLY6978	W303	MATα *nmd2::HIS3 Est2-13Myc::HIS3 ade2-1 trp1-1 can1-100 leu2-3,112 his3-11,15 ura3 GAL+ psi+ ssd1-d2 RAD5*	DLY6737 x DLY6741	This work
DLY6979	W303	MATa *nmd2::HIS3 yku70::LEU2 Est2-13Myc::HIS3 ade2-1 trp1-1 can1-100 leu2-3,112 his3-11,15 ura3 GAL+ psi+ ssd1-d2 RAD5*	DLY6737 x DLY6741	This work

DLY6980	W303	MATa *yku70::LEU2 Est2-13Myc::HIS3 ade2-1 trp1-1 can1-100 leu2-3,112 his3-11,15 ura3 GAL+ psi+ ssd1-d2 RAD5*	DLY6737 x DLY6741	This work

**Strain Collection**	**Genetic Background**	**Construction**	**Reference / Source**
SGAv2p15	S288C	MATa *ura3 leu2 his3 met15 orfX::KANMX*	Charles Boone
SGAv3	S288C	SGAv2 re-arrayed (see methods)	This work.
SGAv2p15 *cdc13-1 DLY5385*	S288C	DLY5385 x SGAv2p15	This work.
SGAv3 *cdc13-1 DLY5385*	S288C	DLY5385 x SGAv3	This work.
SGAv2 *yku70Δ::URA3* DLY3541	S288C	DLY3541 x SGAv2p15	This work.
SGAv3 *yku70Δ::URA3* DLY3541	S288C	DLY3541 x SGAv3	This work.

Oligonucleotide	Sequence	Target
BUD6F	CAGACCGAACTCGGTGATTT	*BUD6*
BUD6R	TTTTAGCGGGCTGAGACCTA	*BUD6*
STN1F	TCGAGCAACTGCAAGAAGAA	*STN1*
STN1R	CGAAATGACAAGGAATGCAC	*STN1*
EST2F	AATTTGACGCTGCAAAAGCTA	*EST2*
EST2R	AGTCCAATACGGTCCCTTCC	*EST2*
TEN1F	ATACACCAAAGTCCGCCAAT	*TEN1*
TEN1R	CACCAAGTGGTGATTTGACA	*TEN1*
CDC13F	AAGAGCCTGAGTGTCCTCCA	*CDC13*
CDC13R	ACGAATTGCACGGGAACTAT	*CDC13*
PDI1F	AAACTCCGCAAGCACCAAGT	*PDI1*
PDI1R	CTGAAGACTCCGCTGTCGTTAA	*PDI1*
VI-RL	CGTATGCTAAAGTATATATTACTTCACTCCATT	VI-R telomere
VI-RR	TCCGAACTCAGTTACTATTGATGGAA	VI-R telomere
XV-LF	AACCCTGTCCAACCTGTCTCC	XV-L telomere
XV-LR	ATCGTGGTTCGCTGTGGTAT	XV-L telomere

Plasmid	Centrometic (CEN) or multicopy (2µ)	Details	Source / Reference
pVL1045	CEN	expresses native *STN1*	Connie Nugent (Petreaca et al., 2007)
pCN284	CEN	expresses native *TEN1*	Connie Nugent (Petreaca et al., 2007)
Ycplac111	CEN	vector only control for pVL1045	Connie Nugent (Petreaca et al., 2007)
Ycplac22	CEN	vector only control for pCN284	Connie Nugent (Petreaca et al., 2007)
pVL1066	2µ	expresses native *STN1*	Connie Nugent (Petreaca et al., 2007)
pPC4	2µ	expresses native *TEN1*	Connie Nugent (Petreaca et al., 2007)
YEplac181	2µ	vector only control for pVL1066	Connie Nugent (Petreaca et al., 2007)
YEplac195	2µ	vector only control for pPC4	Connie Nugent (Petreaca et al., 2007)

Supplemental References

Addinall, S.G., Downey, M., Yu, M., Zubko, M.K., Dewar, J., Leake, A., Hallinan, J., Shaw, O., James, K., Wilkinson, D.J., *et al.* (2008). A genomewide suppressor and enhancer analysis of cdc13-1 reveals varied cellular processes influencing telomere capping in Saccharomyces cerevisiae. Genetics *180*, 2251-2266.

Askree, S.H., Yehuda, T., Smolikov, S., Gurevich, R., Hawk, J., Coker, C., Krauskopf, A., Kupiec, M., and McEachern, M.J. (2004). A genome-wide screen for *Saccharomyces cerevisiae* deletion mutants that affect telomere length. Proc Natl Acad Sci U S A *101*, 8658-8663.

Downey, M., Houlsworth, R., Maringele, L., Rollie, A., Brehme, M., Galicia, S., Guillard, S., Partington, M., Zubko, M.K., Krogan, N.J., *et al.* (2006). A genome-wide screen identifies the evolutionarily conserved KEOPS complex as a telomere regulator. Cell *124*, 1155-1168.

Gatbonton, T., Imbesi, M., Nelson, M., Akey, J.M., Ruderfer, D.M., Kruglyak, L., Simon, J.A., and Bedalov, A. (2006). Telomere length as a quantitative trait: genome-wide survey and genetic mapping of telomere length-control genes in yeast. PLoS Genet *2*, e35.

Petreaca, R.C., Chiu, H.C., and Nugent, C.I. (2007). The role of Stn1p in Saccharomyces cerevisiae telomere capping can be separated from its interaction with Cdc13p. Genetics *177*, 1459-1474.

SGD (2008). Saccharomyces Genome Database.
